# A novel assay to screen siRNA libraries identifies protein kinases required for chromosome transmission

**DOI:** 10.1101/gr.254276.119

**Published:** 2019-10

**Authors:** Mikhail Liskovykh, Nikolay V. Goncharov, Nikolai Petrov, Vasilisa Aksenova, Gianluca Pegoraro, Laurent L. Ozbun, William C. Reinhold, Sudhir Varma, Mary Dasso, Vadim Kumeiko, Hiroshi Masumoto, William C. Earnshaw, Vladimir Larionov, Natalay Kouprina

**Affiliations:** 1 Developmental Therapeutics Branch, National Cancer Institute, National Institutes of Health, Bethesda, Maryland 20892, USA;; 2 School of Biomedicine, Far Eastern Federal University, A.V. Zhirmunsky National Scientific Center of Marine Biology, Far Eastern Branch of Russian Academy of Sciences, Vladivostok, 690000, Russia;; 3 Division of Molecular and Cellular Biology, National Institute for Child Health and Human Development, National Institutes of Health, Bethesda, Maryland 20892, USA;; 4 High-Throughput Imaging Facility, National Cancer Institute, National Institutes of Health, Bethesda, Maryland 20892, USA;; 5 Laboratory of Chromosome Engineering, Department of Frontier Research and Development, Kazusa DNA Research Institute, Kisarazu, Chiba 292-0818d, Japan;; 6 Wellcome Centre for Cell Biology, University of Edinburgh, Edinburgh EH9 3JR, United Kingdom

## Abstract

One of the hallmarks of cancer is chromosome instability (CIN), which leads to aneuploidy, translocations, and other chromosome aberrations. However, in the vast majority of human tumors the molecular basis of CIN remains unknown, partly because not all genes controlling chromosome transmission have yet been identified. To address this question, we developed an experimental high-throughput imaging (HTI) siRNA assay that allows the identification of novel CIN genes. Our method uses a human artificial chromosome (HAC) expressing the *GFP* transgene. When this assay was applied to screen an siRNA library of protein kinases, we identified *PINK1*, *TRIO*, *IRAK1*, *PNCK*, and *TAOK1* as potential novel genes whose knockdown induces various mitotic abnormalities and results in chromosome loss. The HAC-based assay can be applied for screening different siRNA libraries (cell cycle regulation, DNA damage response, epigenetics, and transcription factors) to identify additional genes involved in CIN. Identification of the complete spectrum of CIN genes will reveal new insights into mechanisms of chromosome segregation and may expedite the development of novel therapeutic strategies to target the CIN phenotype in cancer cells.

Chromosome instability (CIN), involving the unequal distribution of chromosomes to daughter cells during mitosis, is observed in the majority of solid tumors (Thompson et al. 2010). CIN may be caused by mutations in or misregulation of a specific set of genes. These so-called CIN genes encode components that control DNA replication, the mitotic checkpoint, and chromosome segregation. Mutations in CIN genes are thought to often be an early event in tumor development, predisposing cells to the accumulation of genetic changes promoting the transition to a cancerous state (Thompson et al. 2010). Conversely, recent findings indicate that because cancer cells often lack protective pathways, CIN may also be a barrier to tumor growth and, therefore, can be exploited therapeutically (Janssen et al. 2009; Swanton et al. 2009).

Currently, approximately 400 human genes are annotated with Gene Ontology (GO) terms associated with proper chromosomal transmission, and systematic CIN gene screens in the yeast *Saccharomyces cerevisiae* have revealed 692 genes (Stirling et al. 2011, 2012). Recently, 245 additional genes whose individual overexpression causes CIN were identified in yeast. These genes were referred to as dosage CIN (dCIN) genes (Duffy et al. 2016). Thus, the combined catalog of yeast genes contributing to chromosome instability consists of 937 genes. The published yeast CIN gene list suggests that many biological processes are involved in the protection of genome integrity. A large proportion of CIN genes function in expected pathways such as in mitosis, DNA replication, and repair, but some act in biological pathways with unknown connections to chromosome segregation (e.g., tRNA synthesis, GPI anchors, and secretion) (Yuen et al. 2007; Stirling et al. 2011, 2012). Because >60% of baker's yeast genes are clearly conserved across diverse organisms including humans, approximately 400 CIN genes in yeast have orthologs in the human genome (Stirling et al. 2012; Duffy et al. 2016). Characterization of these genes in human cells may offer a first step toward completing the annotation of genetic loci controlling chromosome transmission.

Genome-wide siRNA screens have been used to interrogate a variety of molecular mechanisms related to increased sensitivity to ionizing radiation or spontaneous gamma H2AX (phosphorylated histone H2AX at serine 139) (Paulsen et al. 2009; Hurov et al. 2010). In a landmark study, the MitoCheck consortium performed a genome-wide phenotypic siRNA screen against roughly 21,000 human genes using live imaging of fluorescently labeled chromosomes (Hutchins et al. 2010; Neumann et al. 2010). However, a systematic siRNA screen of yeast orthologs in the human genome to identify novel CIN genes has not yet been performed. This may be in part because unequal distribution of chromosomes to daughter cells is currently monitored only through laborious assays, involving karyotype analysis or fluorescent in situ hybridization (FISH).

In the current study, we developed a novel high-throughput imaging (HTI) siRNA assay to identify unknown human CIN genes. This assay is based on a nonessential human artificial chromosome (HAC) expressing a short half-life green fluorescent protein (GFP). This HAC, like other HACs, follows the rules of mitosis and chromosome segregation just like the natural chromosomes during the cell cycle progression (Nakano et al. 2008; Bergmann et al. 2012; Ohzeki et al. 2015; Molina et al. 2017). It is worth noting that the use of yeast artificial chromosomes (YACs) was critical for the discovery and systematic analysis of CIN genes in *S. cerevisiae* (Maine et al. 1984; Spencer et al. 1990; Kouprina et al. 1993; Roberts et al. 1994). We used this novel HAC-based HTI assay to screen a siRNA library targeting human kinases and known yeast CIN orthologs and identified several genes, knockdown of which induces chromosome instability. The discovery of a comprehensive list of CIN genes will shed light on the mechanisms of chromosome transmission and should expedite the development of novel therapeutic strategies to target the CIN phenotype in cancer cells.

## Results

### Experimental system to identify novel human genes controlling proper chromosome transmission

To identify CIN genes, we developed a novel HTI assay that is based on the use of an alphoid^tetO^-HAC (Nakano et al. 2008) carrying a dual cassette simultaneously expressing two destabilized versions of the *GFP* transgene. This HAC, which was assembled from centromeric repeats, contains a functional centromere that allows its relatively stable inheritance as a nonessential chromosome. The HAC loss rate is roughly 10-fold higher when compared with the native chromosomes (Nakano et al. 2008), making the assay very sensitized and allowing a statistically significant number of events in a realistic sample size when studying the CIN phenotype in human cells. Previously, the HAC was used for low-throughput identification of drugs that elevate chromosome instability (CIN) in cancer cells (Lee et al. 2013a, 2016; Kim et al. 2016), as a gene delivery vector for the efficient and regulated expression of exogenous full-length genes in mammalian cells (Iida et al. 2010; Kim et al. 2011; Kouprina et al. 2012, 2013, 2014; Kononenko et al. 2014; Liskovykh et al. 2015; Lee et al. 2018), and for studies of the epigenetic regulation of human kinetochores (Bergmann et al. 2012; Ohzeki et al. 2015; Molina et al. 2017).

**Figure 1.**
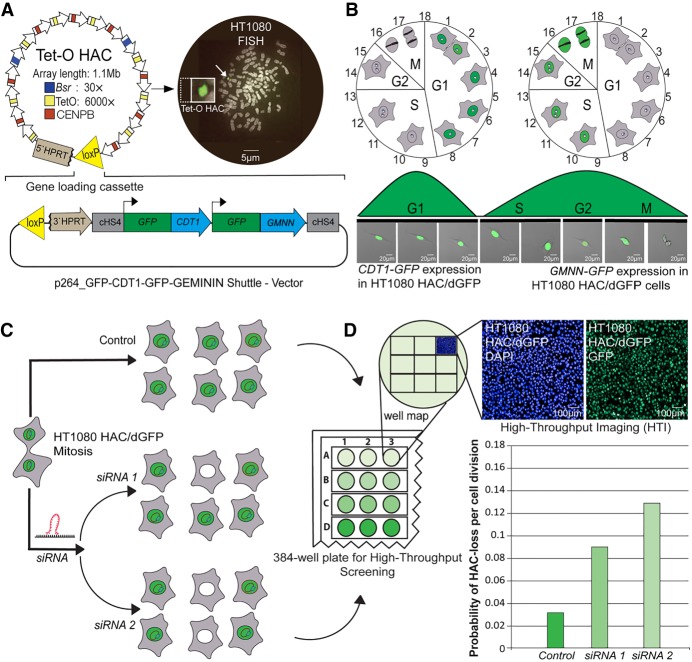
Experimental design of a high-throughput imaging (HTI) human artificial chromosome (HAC)-based assay to identify novel CIN genes via siRNA screening. (*A*) The assay is based on the use of alphoid^tetO^-HAC (Tet-O HAC) (Nakano et al. 2008) expressing a dual short half-life green fluorescent protein GFP-CDT1-GFP-GMNN. Such HAC was named as HAC/dGFP. DNA in situ fluorescence hybridization (FISH) on a metaphase spread of HT1080 cells carrying HAC/dGFP using a probe against the HAC (Methods) and a schematic representation of the HAC/dGFP are shown. (*B*) A schematic representation of the dGFP reporter stability during different phases of the cell cycle in HT1080 cells. Cells that express cell cycle sensors CDT1 and GMNN fused with GFP display green fluorescence during the entire cell cycle. (*C*,*D*) Schematics of siRNA screening using HAC/dGFP-HTI assay to identify CIN genes. HT1080 cells carrying HAC/dGFP are transfected with a nontargeting control siRNA or with siRNA against a gene of interest, knockdown of which induces HAC/dGFP loss (indicated as siRNA1 or siRNA2), in a 384-well imaging plate. Cells are fixed, and the nuclear fluorescence in the GFP channel is measured using HTI. Cells transfected with siRNA against CIN genes display an increase of HAC/dGFP loss compared to the negative (nontargeting) control siRNA treatment.

In the current study, we hypothesized that siRNA-dependent knockdown of the genes that are essential for proper transmission of natural human chromosomes would induce HAC loss. To develop the assay, the plasmid p264-GFP-CDT1-GFP-GEMININ was constructed (Supplemental Fig. S1) containing two modified short half-life green fluorescent transgenes. More precisely, the plasmid encodes two fusions of GFP: GFP-fused with a 30–120 amino acid domain of CDT1, and GFP-fused with a 1–110 amino acid domain of geminin DNA replication inhibitor (GMNN). CDT1 and GMNN are the marker-proteins for different cell cycle stages (Supplemental Movie S1; Sakaue-Sawano et al. 2008). The GFP-CDT1 fusion will cause the HAC-containing cells to be green in the G1 phase of the cell cycle. The GFP-GMNN fusion causes the HAC-containing cells to be green in the S-G2-M phases of the cell cycle (Fig. 1A,B). Thus, the cells carrying the HAC/dGFP should show a robust fluorescent signal in the GFP channel throughout the cell cycle (Supplemental Movie S2) and lose the GFP signal within hours after HAC loss. We called this class of GFP-fusions “destabilized GFP” (dGFP). The p264-GFP-CDT1-GFP-GEMININ plasmid was inserted into the single *loxP* site of an alphoid^tetO^-HAC in hamster CHO cells by Cre-lox-mediated recombination producing HAC/dGFP, which was then transferred via microcell-mediated chromosome transfer (MMCT) to the human HT1080 fibrosarcoma cell line (Fig. 1A; Supplemental Fig. S2).

We predicted two outcomes after siRNA treatment: (1) no change in the percentage of GFP-expressing cells (no effect on HAC stability), or (2) an increase in the percentage of GFP-negative cells if siRNAs induce chromosome segregation errors (Fig. 1C). Control untreated cells containing HAC/dGFP should show uniform green fluorescence. Rapid loss of the GFP signal is critical because loss of fluorescence in the GFP channel after mitosis will allow detection of HAC loss within 72–96 h after siRNA treatment or 9 h after HAC loss. The number of cells without the HAC can be measured using HTI screening (Fig. 1D). In comparison, the use of the standard *GFP* transgene for the same purpose is not applicable owing to the protein's long half-life (Lee et al. 2016). In this case the cells remain green although a target gene is knocked down and HAC is lost, which makes it impossible to use a high-throughput imaging approach. We next set out to test whether the HAC/dGFP-HTI assay can be applied to screen libraries of siRNAs to identify human genes whose knockdown results in chromosome instability.

### Effect of siRNA-mediated knockdown of human genes known to be essential for chromosomal transmission on mitotic stability of HAC/dGFP

To identify an appropriate positive control for our assay, we performed experiments to assess the behavior of the HAC/dGFP-HTI assay following the knockdown of genes essential for kinetochore function. We chose six genes, that is, *CENPA* encoding the centromere-specific histone H3 variant CENPA (Fukagawa and Earnshaw 2014), *CENPN* that participate in the centromeric nucleosome recognition (Carroll et al. 2009), *CENPE* encoding the mitotic centromeric kinesin that participates in microtubule capture (Sardar and Gilbert 2012), *AURKB* encoding the chromosome segregation kinase that forms the chromosomal passenger complex (CPC) (Carmena et al. 2012), *OIP5* encoding the CENPA deposition factor that regulates recruitment of the OIP5 complex to centromeres (Stellfox et al. 2016), and *SKA3* encoding an outer kinetochore protein implicated in microtubule binding (Sivakumar et al. 2014).

**Figure 2.**
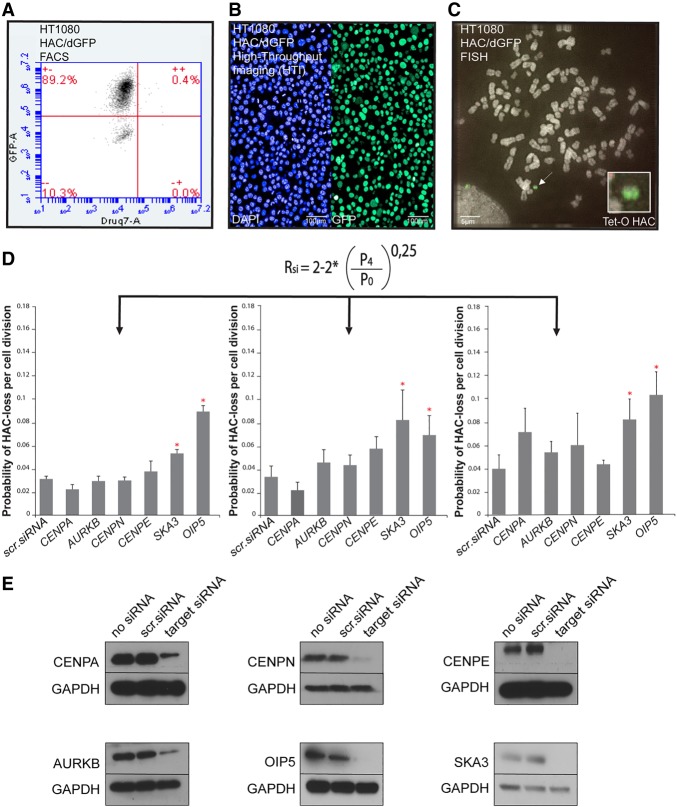
Validation of the HAC/dGFP-HTI assay. Measurement of the proportion of GFP-positive and GFP-negative cells in HT1080 human cells carrying HAC/dGFP treated with a set of siRNAs designed to knockdown known genes essential for chromosomal transmission using flow cytometry (FACS) (*A*), HTI (*B*), and fluorescence in situ hybridization (FISH) (*C*). (*D*) The formula used to determine the rate of HAC loss per generation after siRNA treatment (Methods). (*A–C*) The rate of HAC/dGFP loss after treatment with a set of siRNAs against *CENPA*, *AURKB*, *CENPN*, *SKA3*, and *OIP5* as measured by FACS (*A*), HTI (*B*), and FISH (*C*). The error bars indicate standard deviation. Knockdown of *SKA3* and *OIP5* shows the highest effect on HAC/dGFP loss. Red asterisks indicate siRNA treatment that results in statistically significant difference (*P* < 0.05; *t*-test) when compared to a negative control (scr. siRNA or nontargeting siRNA). (*E*) Western blot analysis confirming silencing efficiency of CENPA, CENPN, CENPE, AURKB, OIP5, and SKA3 proteins (Supplemental Table S8) after siRNA-mediated knockdown of the genes.

Mitotic stability of the HAC/dGFP in HT1080 human cells transfected with siRNAs against either *CENPA*, *CENPN*, *CENPE*, *AURKB*, *OIP5,* or *SKA3* was measured by three independent techniques: flow cytometry (FACS), HTI, and FISH (Fig. 2A–D). The level of each protein reduction was monitored by western blot analysis (Fig. 2E; Supplemental Table S8). Knockdown of *OIP5* and *SKA3* showed the strongest effect, with significant HAC/dGFP loss at 96 h after siRNA transfection. Following these experiments, siRNAs against *SKA3* and *OIP5* were used as positive controls; more specifically, *SKA3* for siRNA screening (HTI) and *OIP5* for FACS experiments.

### A pilot siRNA screen of human orthologs of yeast CIN genes identifies a gene encoding protein kinase PRKCE

In large-scale screens for chromosome instability in yeast, 937 CIN genes were identified (Stirling et al. 2011, 2012; Duffy et al. 2016). Most of these genes function in biological pathways whose mechanism of action on chromosome transmission is as yet unknown. A large number of these CIN genes have human orthologs (Stirling et al. 2012; Duffy et al. 2016), making them good candidates for discovery of new pathways controlling human genome stability.

Among known yeast CIN genes, we chose 28 human orthologs belonging to different functional categories and for which siRNA-mediated knockdown in human cells have been previously reported (Supplemental Table S1). This allowed us to use the verified siRNAs for cell treatment. The following gene orthologs were selected for the analysis: *CNOT6*, *NAT10*, *PIGB*, *TANGO6*, *PIGU*, *PIGS*, *GPN2*, *PRC1*, *IPO11*, *CIAO2B*, *NPEPPS*, *RTN2*, *UAP1*, *MSI1*, *AP2B1*, *PPIP5K1*, *WDR76*, *C12orf10*, *PLCD3*, *MUC4*, *NF1*, *RAB1A*, *MEMO1*, *SMARCAD1*, *RPL13*, *XAB2*, *MYO5B*, and *PRKCE*. They are orthologs of yeast proteins whose down-regulation in yeast leads to chromosomal instability (Stirling et al. 2011, 2012).

**Figure 3.**
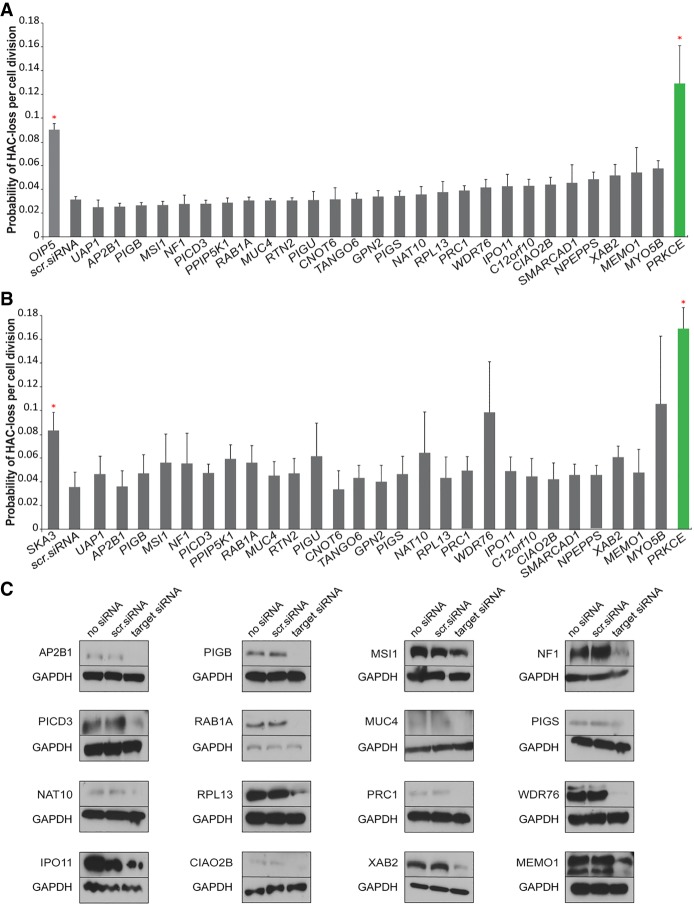
Mitotic stability of the HAC/dGFP in human HT1080 cells treated with a set of siRNAs against 28 human orthologs of yeast CIN genes. A list of gene orthologs selected for the analysis includes *CNOT6*, *NAT10*, *PIGB*, *TANGO6*, *PIGU*, *PIGs*, *GPN2*, *PRC1*, *IPO11*, *CIAO2B*, *NPEPPS*, *RTN2*, *UAP1*, *MSI1*, *AP2B1*, *PPIP5K1*, *WDR76*, *C12orf10*, *PLCD3*, *MUC4*, *NF1*, *RAB1A*, *MEMO1*, *SMARCAD1*, *RPL13*, *XAB2*, *MYO5B*, and *PRKCE* genes. Mitotic stability of HAC/dGFP after knockdown of a target gene was measured by flow cytometry (FACS) (*A*) and HTI (*B*). Among 28 genes analyzed, the strongest effect on HAC/dGFP stability was revealed after cell treatment by siRNA against *PRKCE* (green color and red asterisk). siRNAs against *OIP5* and *SKA3* were used as positive controls for FACS and HTI experiments, correspondingly, and scrambled siRNA (nontargeting siRNA) was used as a negative control. Red asterisks indicate statistical significance (*P* < 0.05; *t*-test) when compared to a negative control. (*C*) Western blot analysis monitoring silencing efficiency of NAT10, PIGB, PIGS, PRC1, IPO11, CIAO2B, MSI1, AP2B1, WDR76, PICD3, MUC4, NF1, RAB1A, MEMO1, RPL13, XAB2, and PRKCE proteins (Supplemental Table S8) after siRNA-mediated knockdown.

Figure 3 shows the rate of HAC/dGFP loss per generation in response to the siRNA knockdown of the aforementioned human genes. Mitotic stability of HAC/dGFP was measured by FACS (Fig. 3A) and HTI (Fig. 3B). Silencing efficiency of the proteins was monitored by western blot analysis (Fig. 3C; Supplemental Table S8). Among 28 siRNA knockdowns analyzed, only the knockdown of *PRKCE* induced a significant increase in HAC/dGFP loss. *PRKCE* is an ortholog of yeast *PKC1*, which is required for yeast cell growth and division (Levin 2005). A failure to detect HAC/dGFP loss after siRNA-mediated knockdown of other genes does not exclude that some of them may be involved in CIN. This can be explained by either (1) a high cytotoxic effect of these siRNAs, that is, the treated cells die faster than they can show any effect on HAC loss, (2) the extreme stability of the target proteins, or (3) insufficient knockdown of a protein to the level that causes hypermorphic or loss of function for the assay being tested.

The human *PRKCE* gene encodes protein kinase C epsilon, which has a variety of functions in different cell types (Scruggs et al. 2016). Recently, involvement of PRKCE in mitotic spindle organization was shown (Brownlow et al. 2014; Martini et al. 2018). More specifically, PRKCE is involved in the control of prophase-to-metaphase progression by coordinating centrosome migration and mitotic spindle assembly (Martini et al. 2018). Because the role of most protein kinases in chromosome transmission is poorly investigated, we chose a siRNA library of human protein kinases for further experiments, using *PRKCE* siRNA as an internal positive control.

### Screening of an siRNA library reveals nine human protein kinases potentially involved in accurate chromosome transmission

**Figure 4.**
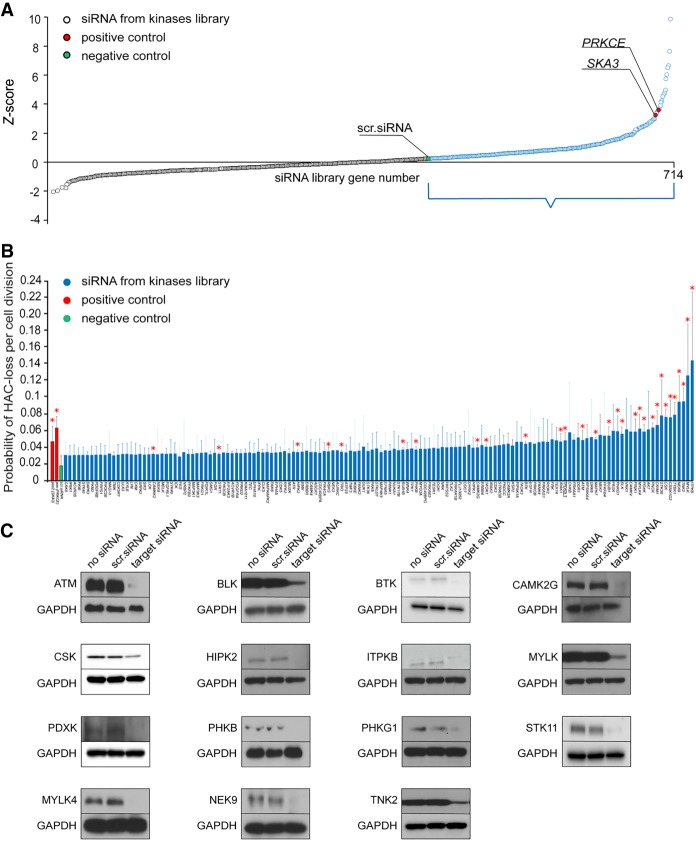
Mitotic stability of HAC/dGFP in human HT1080 cells treated with siRNA library against protein kinases. (*A*) Scatter plot showing a distribution of siRNAs against protein kinases based on *Z*-score. The genes marked in blue and positioned between a negative control (scr. siRNA in green) and the highest score are suitable for the next step of the analysis. *SKA3* and P*RKCE* were used as positive controls (red). (*B*) Mitotic stability of HAC/dGFP in human HT1080 cells treated with siRNA library against protein kinases. siRNAs against *SKA3* and *PRKCE* were used as positive controls, and a scrambled siRNA (scr. siRNA) as a negative control. Among 714 genes analyzed, the strongest effect on HAC loss was shown by siRNAs against *ITPKB*, *IRAK1*, *MYLK*, *TNK2*, *STK38*, *BLK*, *MAPK7*, *FRK*, *TRIO*, *STK11*, *CRIM1*, *CSK*, *PDXK*, *PHKG1*, *KSR2*, *CAMK2G*, *PHKB*, *CSNK1G2*, *MYLK4*, *TPD52L3*, *RBKS*, *TTBK1*, *PNCK*, *PINK1*, *BTK*, *HIPK2*, *BUB1*, *ATM*, *BUB1B*, *TAOK1,* and *NEK9*. Red asterisks indicate statistical significance (*P* < 0.05; *t*-test) when compared to a negative control. (*C*) Western blot analysis confirming silencing efficiency of ATM, BLK, BTK, CAMK2G, CSK, HIPK2, ITPKB, MYLK, PDXK, PHKB, PHKG1, STK11, MYLK4, NEK9, and TNK2 proteins (Supplemental Table S8) after siRNA-mediated knockdown.

A siRNA library against 714 genes previously annotated as either kinases or phosphatases was used for analysis (Supplemental Fig. S3). In this arrayed library, each well contained a pool of four independent siRNAs targeting the same gene. We optimized the transfection conditions of the library to maximize cell viability while still maintaining efficient siRNA knockdown (i.e., gene down-regulation should not lead to cell death and the number of cells should be enough to permit statistically significant calculations) (Methods; Supplemental Fig. S4). In these experiments, *SKA3* siRNA knockdown was used as a positive control. Figure 4A illustrates the distribution of siRNAs against protein kinases based on their *Z*-score (the absolute value of *Z* represents the distance between the raw score and the population mean in units of the standard deviation) (Supplemental Fig. S4). In further analysis we focused on siRNAs that did not show high cytotoxic effects (Fig. 4B). Figure 4B shows the percentage of HAC/dGFP loss per cell division scored based on the proportion of GFP-negative cells (Methods). Red asterisks indicate statistical significance (*P* < 0.05) when compared to the negative control. Among 714 genes analyzed, the strongest effect on HAC/dGFP stability was detected after siRNA-mediated knockdown of the *ITPKB*, *IRAK1*, *MYLK*, *TNK2*, *STK38*, *BLK*, *MAPK7*, *FRK*, *TRIO*, *STK11*, *CRIM1*, *CSK*, *PDXK*, *PHKG1*, *KSR2*, *CAMK2G*, *PHKB*, *CSNK1G2*, *TAOK1*, *MYLK4*, *NYD-SP25*, *RBKS*, *TTBK1*, *PNCK*, *PINK1*, *BTK*, *HIPK2*, *BUB1*, *ATM*, *BUB1B*, *PRKCE*, *TAOK1*, and *NEK9* genes. Silencing efficiency of the proteins was monitored by western blot analysis (Fig. 4C; Supplemental Table S8).

**Figure 5.**
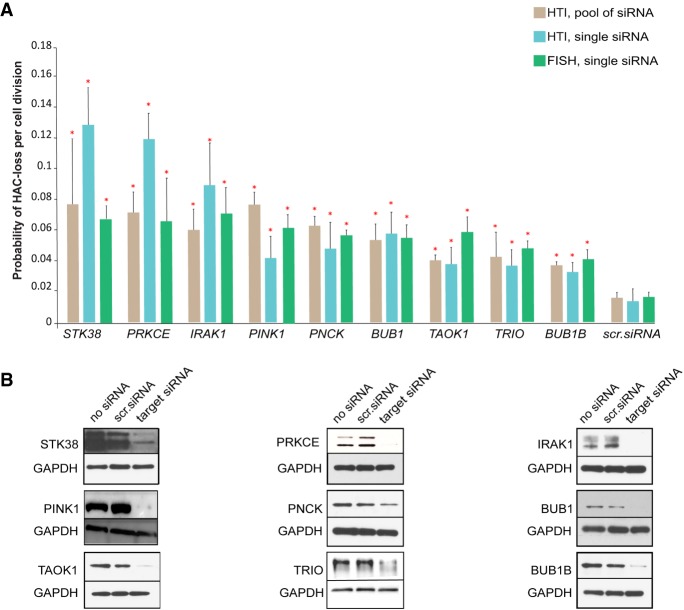
The reconfirmed final list of the CIN gene candidates. (*A*) The rate of HAC/dGFP loss after knockdown of gene candidates was measured by three independent approaches: (1) after siRNA-mediated knockdown using a pool of siRNAs (brown; the rate was measured by HTI as a proportion of nonfluorescent cells); (2) after knockdown of the target gene using one single siRNA sequence (blue; the rate was measured by HTI); and (3) after knockdown of a target gene using one independent siRNA sequence (green; the rate was measured by FISH). The red asterisks indicate statistical significance (*P* < 0.05; *t*-test) when compared to a negative control (scr. siRNA or nontargeting siRNA). (*B*) Silencing efficiency of each protein was monitored by western blot analysis (Supplemental Table S8).

Those 33 primary candidates were reanalyzed using independent siRNAs either found in the literature or made by the company (Supplemental Table S1). Figure 5A shows the comparison of the rates of HAC/dGFP loss for nine reconfirmed CIN candidate genes using three independent approaches: (1) after siRNA-mediated knockdown using a pool of siRNAs from the library of human protein kinases (brown); (2) after knockdown of each target gene using one independent siRNA (blue); and (3) the rate of HAC/dGFP loss was verified by FISH analysis (green). The level of each protein reduction was monitored by western blot analysis (Fig. 5B; Supplemental Table S8). After these experiments, a final list of the CIN candidates included the following genes: *PINK1*, *STK38*, *TRIO*, *IRAK1*, *PNCK*, *TAOK1*, *BUB1*, *BUB1B*, and *PRKCE* (Supplemental Fig. S3; Supplemental Table S2). It is worth noting that the human *BUB1* and *BUB1B* genes are known to promote the spindle assembly checkpoint, which is important for proper chromosome transmission (Chan et al. 1999; Vleugel et al. 2015; Jia et al. 2016), and the *STK38* gene is required for proper centrosome duplication, precise alignment of mitotic chromosomes, and ensures proper spindle orientation in mitosis (Hergovich et al. 2007; Chiba et al. 2009; Yan et al. 2015). Identification of these three genes along with *PRKCE* in the library supports the conclusion that the HAC/dGFP-HTI assay works adequately.

### Knockdown of *PINK1*, *STK38*, *TRIO*, *IRAK1*, *PNCK*, *TAOK1*, *BUB1*, *BUB1B*, and *PRKCE* genes leads to natural chromosome instability and an increased number of double-stranded breaks

**Figure 6.**
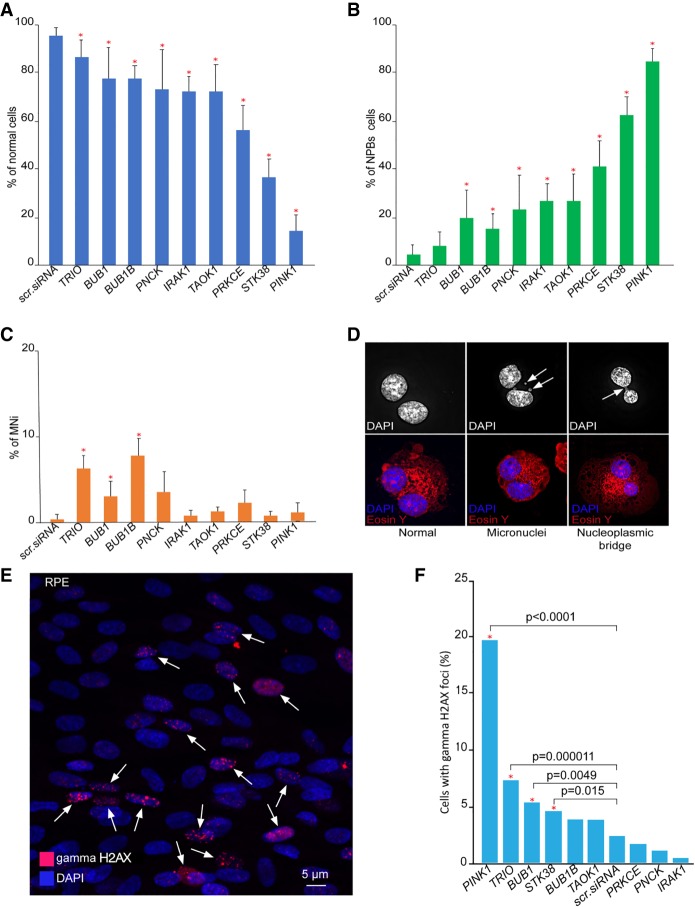
(*A–D*) Micronuclei (MNi) and nucleoplasmic bridges (NPBs) formation in RPE cells after knockdown of one of the following genes: *TRIO*, *BUB1*, *BUB1B*, *PNCK*, *IRAK1*, *TAOK1*, *PRKCE*, *STK38*, and *PINK1*. (*A*) The percentage of the binucleated cells without abnormalities. (*B*) The percentage of NPBs. (*C*) The percentage of MNi. Scrambled siRNA (nontargeting siRNA) was used as a negative control. Error bars correspond to a SD of four replicates. Red asterisks indicate statistical significance when compared to the control (calculated by Fisher's exact test with Bonferroni correction for multiple testing, *P* < 0.0011). (*D*) A normal binucleated cell; a binucleated cell containing three MNi; a cell containing one NPB. White arrows point to MNi and NPBs. The cells were stained with DAPI and Eosin Y. (*E*,*F*) Immunostaining of double-stranded breaks (DSBs) with an antibody against gamma H2AX in interphase of RPE cells after knockdown of *PINK1*, *TRIO*, *BUB1*, *STK38*, *BUB1B*, *TAOK1*, *PRKCE*, *PNCK*, and *IRAK1* genes. (*E*) Examples of immunostaining of the cells. Red signals show gamma H2AX staining as a marker for DSBs. Accumulation of gamma H2AX foci occurred at day 3 in all cases. White arrows point to the cell nuclei with gamma H2AX signals. (*F*) A statistical effect of gamma H2AX foci after knockdown of a target gene. A statistical effect was determined at day 3. For *PINK1*, *TRIO*, *BUB1*, and *STK38* genes, statistically significant (Fisher's exact test: *P*-value; two-tailed) results when compared to a negative control (scr. siRNA or nontargeting siRNA) are indicated with square brackets and red asterisks.

Micronucleus formation assays (MNi) have been extensively used to evaluate chromosome instability (Kirsch-Volders et al. 1997). In addition, formation of nucleoplasmic bridges (NPBs) is a sensitive measure of chromosome damage leading to chromosomal instability (Thomas et al. 2003). To investigate whether the knockdown of *STK38*, *IRAK1*, *PINK1*, *PNCK*, *TAOK1*, *TRIO*, *PRKCE*, *BUB1*, and *BUB1B* genes lead to instability of the natural chromosomes, we performed MNi and NPBs assays in nontransformed retinal pigmented epithelial (RPE) cells. This assay revealed a significant difference in NPB formation between cells treated with scrambled siRNA (nontargeting) and the cells depleted for these genes (Fig. 6A,B,D). The percentage of NPBs after knockdown of *STK38*, *IRAK1*, *PINK1*, *PNCK*, *TRIO*, *TAOK1*, *PRKCE*, *BUB1*, and *BUB1B* genes was elevated compared to the negative control. The highest effect was observed for *PINK1*, *STK38,* and *PRKCE* genes (19-, 14-, and ninefold elevation, respectively) (Supplemental Table S3). Indeed, inhibition of *PRKCE* has previously been shown to result in chromosome bridging (Brownlow et al. 2014). In our experiments, we also measured the formation of MNi. The highest effect was observed for *BUB1B*, *TRIO*, *PNCK*, and *BUB1* genes (30-, 25-, 14-, and 12-fold elevation, respectively) (Fig. 6A,C,D; Supplemental Table S3). When the same experiments were performed in human fibrosarcoma HT1080 cells, the percentage of NPBs formation was elevated after knockdown of all nine of these proteins compared to the control (Supplemental Fig. S5A,C; Supplemental Table S4). The percentage of MNi formation was also elevated after knockdown of these genes except for *TRIO* compared to the control (Supplemental Fig. S5A,B; Supplemental Table S4).

The elevated frequencies of binucleated cells with MNi and NPBs (measures of genome damage and chromosomal instability) support the hypothesis that *PINK1*, *TRIO*, *IRAK1*, *TAOK1*, and *PNCK* gene products are required for accurate chromosome transmission. Identification of *BUB1*, *BUB1B*, *STK38*, and *PRKCE* genes, which were previously known to be involved in proper chromosome transmission (Chan et al. 1999; Hergovich et al. 2007; Chiba et al. 2009; Vleugel et al. 2015; Jia et al. 2016; Martini et al. 2018), in these experiments strongly supports the utility of the HAC/dGFP-HTI assay for screening new CIN genes.

To determine whether the observed chromosome instability was accompanied by an increased number of double-stranded breaks (DSBs), we stained RPE cells after knockdown of *PINK1*, *STK38*, *IRAK1*, *PNCK*, *TAOK1*, *TRIO*, *PRKCE*, *BUB1*, and *BUB1B* genes with an antibody against phosphorylated histone gamma H2AX. A statistically significant increase of gamma H2AX foci in interphase was observed after knockdown of the four genes, *PINK1*, *TRIO*, *STK38*, and *BUB1* (Fig. 6E,F). The strongest effect was observed after knockdown of *PINK1* (20% of cells) compared to control levels of DNA damage in RPE cells (<3%). Thus, in RPE cells chromosome instability after knockdown of *PINK1*, *TRIO*, *STK38*, *BUB1*, and *BUB1B* genes is accompanied by induction of DSBs. On the contrary, the number of H2AX foci in HT1080 cells changed little after siRNA knockdown of the same genes, possibly because of the high endogenous level of DNA damage in these cells. The negative control in HT1080 cells showed ∼8% of spontaneous DNA damage, masking possible effects of siRNA treatment (Supplemental Fig. S6).

### Knockdown of *PINK1*, *STK38*, *TRIO*, *IRAK1*, *TAOK1*, and *PNCK* genes disrupts mitotic progression

To explore the mechanism(s) by which knockdown of the newly identified CIN genes results in chromosome loss, we performed an additional set of siRNA-mediated knockdown experiments. To rule out cancer cell line–specific phenotypes in HT1080, all experiments were also performed in nontransformed RPE cells. Changes affecting only HT1080 cells were considered specific for this cell line.

We first measured the mitotic index in RPE and HT1080 cells after siRNA knockdown of *PINK1*, *STK38*, *TRIO*, *PNCK*, *IRAK1*, *TAOK1*, *BUB1*, or *BUB1B*. In RPE cells, no statistically significant increase in the mitotic index was observed (Supplemental Fig. S7A), although we could observe an increased number of prophases following *STK38* knockdown (Supplemental Fig. S7B). In HT1080 cells, we observed a statistically significant increase of the mitotic index, but no statistically significant change in the distribution of mitotic phases following knockdown of *PINK1* and *IRAK1* (Supplemental Fig. S8A–E). Knockdown of *PINK1*, *STK38*, *TRIO*, *TAOK1,* and *PRKCE* in RPE cells led to an increased number of mitotic abnormalities (Supplemental Fig. S9A); whereas in HT1080 cells, this phenotype was observed only after knockdown of *STK38* and *TAOK1* (Supplemental Fig. S9B). The results of these experiments suggest that *PINK1*, *STK38*, *TRIO*, *TAOK1*, and *PRKCE* genes may be necessary for mitotic progression and for maintenance of the cell cycle. Earlier it was shown that the *STK38* gene regulates essential processes, such as centrosome duplication (Hergovich et al. 2007) and cell cycle/mitotic progression (Emoto et al. 2006) and *PRKCE* is involved in mitotic spindle organization (Brownlow et al. 2014; Martini et al. 2018) that supports the utility of our assay for screening new CIN genes.

**Figure 7.**
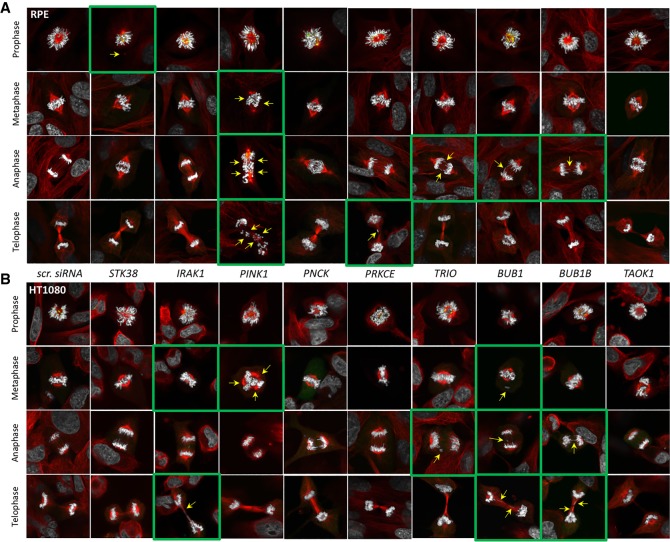
Analysis of localization of tubulin alpha and MAD1L1 at the different stages of mitosis after knockdown of *STK38*, *IRAK1*, *PINK1*, *PNCK*, *PRKCE*, *TRIO*, *BUB1*, *BUB1B*, and *TAOK1* genes in RPE (*A*) and HT1080 (*B*) cells. (scr. siRNA) negative control. Staining by antibodies against tubulin alpha is marked in red; against MAD1L1, in white. Green squares and yellow arrows point to the observed mitotic abnormalities.

We next characterized in more detail the mitotic defects at the different stages of mitosis observed after siRNA knockdown of CIN gene candidates in RPE and HT1080 cells. The cells were stained with antibodies against tubulin alpha to visualize the mitotic spindles (Romaniello et al. 2018) and against mitotic arrest deficient 1 like 1 (MAD1L1), an evolutionarily conserved core mitotic checkpoint protein that monitors kinetochore-microtubule attachment (Luo et al. 2018). In RPE cells, knockdown of several genes revealed a range of mitotic abnormalities (Fig. 7A). Knockdown of *STK38* caused problems with mitotic spindle formation (absence of the connection to one spindle pole) in prophase. Knockdown of *PINK1* led to severe problems with chromosome alignment in metaphase, anaphase, as well as lagging chromosomes in early telophase. Knockdown of *TRIO*, *BUB1*, and *BUB1B* resulted in formation of chromatin bridges in anaphase. All these phenotypes can lead to aneuploidy, chromosome damage, and micronucleus formation. In HT1080 cells, we also observed a wide range of mitotic abnormalities in siRNA knockdown cells (Fig. 7B). Similar to the phenotypes observed in RPE cells, knockdown of *PINK1* indicated severe problems with spindle formation in metaphase (multiple polarity). Knockdown of *TRIO* correlated with chromosomal loss at anaphase possibly caused by kinetochore attachment problems. Knockdown of *BUB1* and *BUB1B* showed lagging and bridging chromosomes at anaphase.

In control siRNA rescue experiments, we attempted to exclude that off-target effects might account for the most unexpected mitotic abnormalities induced by siRNA-mediated knockdown of these genes. We therefore ectopically expressed siRNA-resistant cDNAs encoding *PINK1* and *TRIO* in RPE cells and tested their ability to rescue the knockdown phenotypes. In both cases, mitotic abnormalities caused by knockdown of these genes were rescued by expression of the corresponding cDNAs (Supplemental Figs. S10, S11). In addition, to evaluate the observed phenotypes, we performed live imaging analyses of *PINK1*, *TRIO*, *IRAK1*, *PNCK*, *TAOK1*, and *STK38* genes (Supplemental Methods). The analyses confirmed the mitotic defects observed after siRNA knockdown of these genes (Supplemental Movies S3–S9). Live imaging analyses of *PINK1*, *IRAK1*, *TRIO*, and *STK38* showed that formation of lagging chromosomes leads to micronuclei formation.

**Figure 8.**
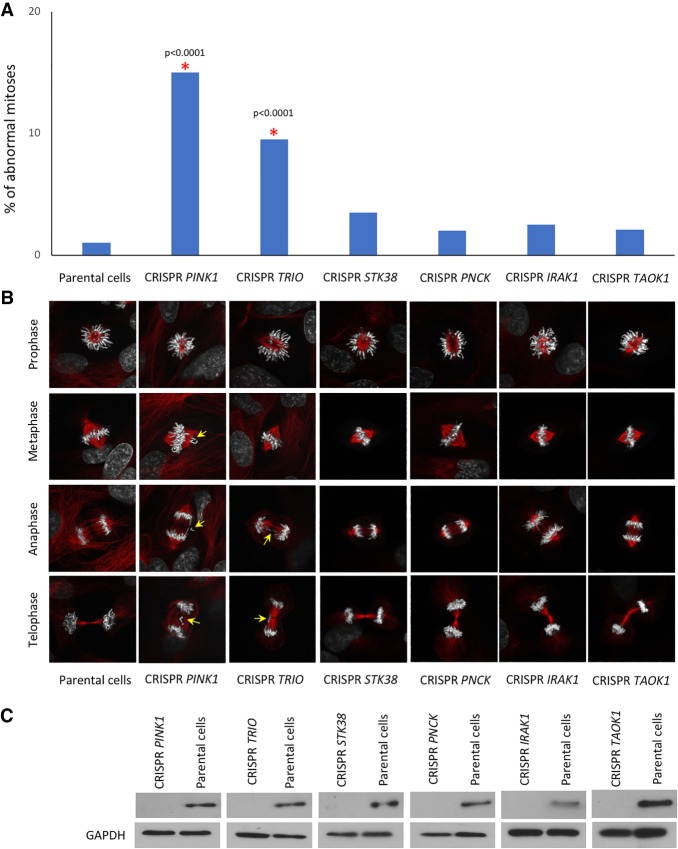
CRISPR/Cas9 disruption of the *PINK1*, *TRIO*, *IRAK1*, *PNCK*, *STK38*, and *TAOK1* genes. (*A*) Percentage of abnormal mitoses counted after CRISPR/Cas9 disruption of the *PINK1*, *TRIO*, *IRAK1*, *PNCK*, *STK38,* and *TAOK1* genes. For statistical significance, Fisher's exact test was applied. A red asterisk indicates statistical significance (*P* < 0.05) in comparison with negative control (Parental cells). About 150 mitotic events were analyzed. (*B*) Immunostaining of the cells after CRISPR/Cas9 knockout against tubulin alpha (red) counterstained with DAPI to observe mitotic abnormalities. Yellow arrows point to the identified mitotic abnormalities. (*C*) Western blot analysis confirming absence of PINK1, TRIO, IRAK1, PNCK, STK38, and TAOK1 proteins after CRISPR/Cas9-mediated knockout of these genes (Supplemental Table S8).

In addition, we prepared CRISPR/Cas9 knockouts (Supplemental Methods) for the five kinases of greatest interest to us (*PINK1*, *TRIO*, *IRAK1*, *PNCK*, and *TAOK1*) and determined the resulting phenotypes. *STK38* was included as a control because, as previously shown, this gene is involved in proper chromosome transmission (Hergovich et al. 2007; Chiba et al. 2009; Yan et al. 2015). In two cases, for *PINK1* and *TRIO*, we observed the problems with chromosomes alignment and kinetochore attachment (Fig. 8). The more pronounced phenotypes scored after siRNA knockdown of these genes compared to CRISPR/Cas9-induced gene disruption may be explained by the following reasons. In the case of siRNA-mediated knockdown, we analyzed problems with chromosome alignment within 96 h of siRNA transfection. In contrast, CRISPR/Cas9-mediated defects were observed after cell selection, which takes 7 d. Cells may up-regulate compensatory pathways during this selection period, and cells with the highest levels of abnormalities may not survive.

After *STK38*, *IRAK1*, *PNCK*, and *TAOK1* CRISPR/Cas9-induced gene disruption, we did not observe the phenotypes similar to that after siRNA knockdown of these genes (Fig. 8). The same result was obtained with the *STK38* gene that is required for mitosis progression (Hergovich et al. 2007; Chiba et al. 2009; Yan et al. 2015). Therefore, the negative results obtained after CRISPR/Cas9 knockout experiments do not exclude that *IRAK1*, *PNCK*, and *TAOK1* are involved in the control of chromosome transmission. It should be noted that there are several publications on side-by-side comparison of CRISPR/Cas9 and RNAi screens (Deans et al. 2016; Morgens et al. 2016; Schuster et al. 2019), indicating that the two screening technologies may identify different biological categories of genes or showed little correlation, which can be partially explained by the identification of distinct essential biological processes with each technology.

To summarize the preceding experiments, we conclude that five genes, *PINK1*, *IRAK1*, *PNCK*, *TAOK1*, and *TRIO* may be considered as novel CIN genes involved in the control of chromosome transmission in human cells.

## Discussion

Compared to yeast, for which essentially every gene has been checked for its role in chromosome transmission, only a moderate fraction of genes that control proper transmission of chromosomes has been annotated in humans (Paulsen et al. 2009; Hurov et al. 2010; Hutchins et al. 2010; Neumann et al. 2010). This difference is mainly attributable to the development of conceptually simple color colony assays in yeast that provided a powerful high-throughput genetic tool to assess the rates of chromosome mis-segregation and to identify mutants deficient in this process (Spencer et al. 1990). The final list of yeast genes involved in controlling chromosome stability consists of 937 genes (Stirling et al. 2012; Duffy et al. 2016). This catalog of genes revealed a number of human orthologs known to be recurrently overexpressed and/or amplified in tumors (Duffy et al. 2016). However, a large number of human CIN genes remains to be discovered in humans. Identification of these genes would be a first step toward completing the annotation of genetic loci controlling chromosome transmission in humans.

In the current study, we developed a novel HTI assay for identification of genes controlling chromosome transmission in human cells. Our strategy uses a nonessential human artificial chromosome, the alphoid^tetO^-HAC (Nakano et al. 2008), expressing a dual short half-life green fluorescent protein dGFP (Sakaue-Sawano et al. 2008). The HAC/dGFP-HTI assay was used to screen a siRNA library of human protein kinases and identified five new candidate CIN genes, that is, *PINK1*, *TRIO*, *IRAK1*, *PNCK,* and *TAOK1*, knockdown of which leads to elevated frequencies of binucleated cells with micronuclei and chromatin bridges (both measures of genome damage and chromosome instability). All these genes have previously been associated with human disorders. Earlier biochemical and genetic studies revealed that *PINK1*, which has been associated to Parkinson's disease, works together with *PRKN* (also known as *parkin*) in the pathway involved in mitochondrial quality control (Pickrell and Youle 2015). *TRIO* is an essential gene with a prominent role in the development of the nervous system. *TRIO* expression is significantly increased in different types of tumors and has been proposed to participate in oncogenesis (Schmidt and Debant 2014). *IRAK1* is associated with pediatric systemic Lupus Erythematosus and Lubs X-linked mental retardation syndrome (Gottipati et al. 2008). *PNCK* is overexpressed in a subset of breast tumors and linked to Jervell and Lange-Nielsen Syndrome 1 (Wu et al. 2013). *TAOK1* is involved in the cell cycle and signaling by Rho GTPases pathways (Raman et al. 2007). To our knowledge, neither *PINK1*, *TRIO*, *IRAK1*, *PNCK*, or *TAOK1* genes have previously been linked to CIN. It is known that in yeast a large fraction of CIN genes has been originally identified as those that function in pathways with unknown connections to chromosome segregation (e.g., tRNA synthesis, GPI anchors, and secretion) (Yuen et al. 2007; Stirling et al. 2011, 2012).

Here, we went on to show that siRNA knockdown of *PINK1*, *STK38*, *TRIO*, *TAOK1*, and *PRKCE* causes problems during mitosis progression. More specifically, knockdown of *PINK1* leads to severe defects in metaphase and anaphase chromosome alignment as well as lagging chromosomes in telophase (Supplemental Fig. S12). Knockdown of *STK38* was associated with problems in mitotic spindle formation in prophase, whereas knockdown of *TRIO*, *BUB1,* and *BUB1B* was associated with formation of chromatin bridges in anaphase (Supplemental Fig. S12). All these phenotypes can lead to aneuploidy, chromosome damage, and micronucleation formation. We also showed that knockdown of *PINK1, TRIO, STK38, BUB1,* and *BUB1B* induces formation of DSBs that may also cause chromosome instability.

Our results are supported by bioinformatical data. The NCI-60 cell lines derived from nine tissues of origin types of cancer have been characterized for multiple parameters, including transcript expression (Reinhold et al. 2012, 2015). Bioinformatical analysis of the NCI-60 database using NCI-60 expression data from five different microarray platforms (Methods) showed a significant correlation between down-regulation of *PINK1*, *TRIO*, *IRAK1*, *BUB1*, and *BUB1B* and an increased level of cytogenetic alterations (Supplemental Table S5).

In addition, we constructed a gene interaction network map that represents potential functional relationships among the CIN kinases, PINK1, STK38, TRIO, IRAK1, PNCK, and TAOK, and the proteins involved in cell division and cell cycle regulation (Supplemental Fig. S13). The most frequent relationship was protein–protein interactions (54), followed by activation (27) and phosphorylation (21) (Supplemental Table S6). Knowledge about interacting proteins is crucial for understanding their biological functions, which can be performed by studying networks of these interactions. Also, this network may also help in planning the future experiments to shed light on the role of these kinases in the complex process of chromosome transmission.

Identification of novel CIN genes is crucial for understanding the molecular mechanisms of mitotic regulation. Analysis of a role of each CIN gene identified in this study in the complex process of chromosome transmission will be the subject of future investigations. In addition, because CIN represents a vulnerability that can be exploited as a therapeutic avenue for treatment of cancer (Janssen et al. 2009; Colombo and Moll 2011; Bakhoum and Compton 2012; Giam and Rancati 2015), the CIN genes identified in this study introduce potential biomarkers that may expedite the development of new therapeutic strategies that target cancer cells. In the future, the HAC/dGFP-HTI assay can be applied for screening different siRNA libraries (such as those targeted toward cell cycle regulation, DNA damage response, epigenetics, transcription factors) and for genome-wide screening to identify other genes involved in CIN. We admit that some genes may be missed during siRNA libraries screening. This may be caused by a high cytotoxic effect of some siRNAs as has been observed for siRNA against *AURKB* in this study or a high stability of the protein such as CENPA. For such genes, other approaches can be applied. For example, gene overexpression was used for identification of CIN genes (Duffy et al. 2016). Nevertheless, the fact that this assay can identify genes previously unknown to have any connection with chromosome segregation suggests that further characterization of these novel CIN genes may reveal previously unsuspected aspects of mitotic control.

## Methods

### Construction of the p264-GFP-CDT1-GFP-GEMININ vector

At the first step of construction, the 1043- and 1091-bp fragments of the cell cycle sensors, GFP-CDT1 and GFP-GMNN, containing the coding region of the GFP were PCR-amplified from GFP-CDT1 and GFP-GMNN synthetized gBlocks (Integrated DNA Technologies [IDT]) using the corresponding primers (Supplemental Table S7). The primers contain EcoRI restriction sites at the 5′ ends of the fragments, which are necessary for the further steps of construction. The amplified products were ligated with the EcoRI-digested pCX vector producing the pCX-GFP-CDT1 vector of 5813 bp in length and pCX-GFP-Geminin vector of 5861 bp in length. Each sensor and a green fluorescent protein are under the SV40 virus promoter (Supplemental Fig. S1A,B). The second step of construction included restriction of the pCX-GFP-CDT1 and pCX-GFP-Geminin vectors with BamHI/SpeI and AvrII/SpeI, respectively, that produced two fragments of 465 and 3700 bp in length, respectively. The third step of construction included ligation of the BamHI/SpeI pCX-GFP-CDT1 fragment with the linearized p264 vector (Lee et al. 2013b) producing the p264_GFP-CDT1vector (Supplemental Fig. S1C). The fourth step of construction included ligation of the AvrII/SpeI pCX-GFP-Geminin fragment with the AvrII-digested p264_GFP-CDT1 vector producing the p264_GFP-CDT1-GFP-GEMININ vector (Supplemental Fig. S1D). The final p264-GFP-CDT1-GFP-GEMININ vector contains the open reading frames of GFP-CDT1 and GFP-GMNN, each under control of the CAG promoter that allows their expression in hamster CHO and human HT1080 cells. The final p264-GFP-CDT1-GFP-GEMININ vector contains a single *loxP* site and a 3′ part of the *HPRT* gene flanked by the cHS4 insulators that is essential for its loading into the alphoid^tetO^-HAC by Cre-lox-mediated recombination. Primers used for plasmids construction are provided in Supplemental Table S7.

### Loading of p264-GFP-CDT1-GFP-GEMININ vector into alphoid^tetO^-HAC in hamster CHO cells

Two micrograms of the p264-GFP-CDT1-GFP-GEMININ vector and 0.2 µg of the Cre expressing pCpG-iCre vector DNA were cotransfected into HPRT-deficient hamster CHO cells containing the alphoid^tetO^-HAC with a single *loxP* site by lipofection using X-tremeGENE 9 (Roche). HPRT-positive colonies were selected after 2–3 wk growth in HAT medium. For each experiment, from 10 to 15 clones were usually selected. The correct loading of the p264-GFP-CDT1-GFP-GEMININ vector in the HAC was confirmed by PCR using a specific pair of primers to detect reconstitution of the *HPRT* gene (Supplemental Table S7). The final construct was designated as HAC/dGFP (Supplemental Fig. S2).

### Microcell-mediated chromosome transfer

MMCT transfer of HAC/dGFP from hamster CHO cells to human HT1080 cells was performed as described previously (Liskovykh et al. 2016).

### FISH analysis

The presence of the HAC in an autonomous form was confirmed by FISH analysis as previously described (Nakano et al. 2008; Iida et al. 2010; Kim et al. 2011; Supplemental Methods).

### Generation of HT1080/pCX-CDT1-GFP and HT1080/pCX-GEMININ-GFP cell lines for time-lapse microscopy

Human HT1080 cells were transfected with the pCX-CDT1-GFP and pCX-GEMININ-GFP vectors described above (Supplemental Fig. S1A,B). Per one well of a six-well plate, 150,000 cells were seeded and transfected by 2 µg of each plasmid using a standard protocol provided by DNA Transfection Reagent X-tremeGENE 9 (Roche). To select the cells with stable GFP expression, we used MoFlo Astrios EQ cell sorter (Beckman Coulter). The cells were sorted and seeded on 96-well plate. The clones with the brightest GFP expression were taken for the time-lapse microscopy experiment.

### Time-lapse microscopy

HT1080 cells containing HAC/dGFP and HT1080 cells transfected by either pCX-CDT1-GFP or pCX-GEMININ-GFP vectors were seeded (1000 cells per cm^2^) on a separate µ-Slide 8 Well (ibidi) in DMEM (Thermo Fisher Scientific) supplemented with 10% (v/v) fetal bovine serum (Clontech Laboratories) at 37°C in 5% CO_2_ atmosphere. Time-lapse imaging was performed using the FV1200 confocal laser scanning microscopy system equipped with the objective lens (Olympus, UPLSAPO 20× NA = 0.75). A 405 nm LD Laser with Integrated Transmitted Light Photomultiplier Detector and 488 nm Argon laser with High-Sensitivity Detector (GaAsP) were used. To avoid cross detection, the images were acquired sequentially at 488 nm (Argon) and 405 nm (LD). The transmitted light signal and GFP fluorescence were merged for each confocal image. The recording interval was 15 min.

### Flow cytometry

The HT1080 containing HAC/dGFP cells were grown for 96 h after transfection, harvested by trypsin-treatment, and resuspended in PBS containing 3 µM DRAQ7. Flow cytometry was performed on an BD Accuri C6. All samples were vortexed immediately before flow cytometry examination. Fluorescence of GFP-positive cells was measured by the 488 nm laser and detected at 510 nm. The dead cells were counted by DRAQ7 fluorescence excited by the 640 nm laser and detected at 722 nm. Samples were acquired in at least three separate triplicates for 30 sec or 1 × 10^4^ events (at minimum). Flow cytometry analysis was primarily performed using C-Flow Plus (BD Biosciences).

### siRNA transfection using 24-well plate

The genes of interest were knocked down using siRNAs (Supplemental Table S1) purchased from Dharmacon. For siRNA treatment, 12.5 × 10^3^/well cells were seeded in 24-well plates a day before the experiment. Cells were transfected with each siRNA (a working concentration 17 nM) using Lipofectamine RNAiMAX (Thermo Fisher Scientific) following the manufacturer's protocol. Cells were grown without blasticidine for 96 h after transfection. Silencing efficiency of each protein was monitored by western blot analysis (Supplemental Table S8). After 96 h, the cells were collected and analyzed by flow cytometry to detect the proportion of cells that reactivated GFP fluorescence or lost GFP signal. All the experiments were performed in triplicate.

### siRNA transfection using 24-well plates for rescue experiments

The rescue experiments were performed as described in the Lipofectamine RNAiMAX protocol (Thermo Fisher Scientific). The genes of interest were depleted using siRNAs (Supplemental Table S1), which were purchased from Dharmacon. For siRNA treatment, 12.5 × 10^3^/well cells were seeded in 24-well plates a day before the experiment. Cells were transfected with each siRNA (a working concentration of 12 nM) using Lipofectamine RNAiMAX (Thermo Fisher Scientific). To complement siRNA effect, cotransfection of siRNA and cDNA resistant to siRNA was performed (for *PINK1* cDNA, from GenScript OHu25380; for *TRIO* cDNA, from GenScript OHu25435). Cells were grown for 96 h after transfection. After 96 h, the cells were fixed, immunostained, and analyzed by confocal microscopy. All the experiments were performed in triplicates.

### siRNA oligo library preparation

High-throughput imaging of siRNA screen was performed in 384-well plates. The library used in screening includes siRNA oligos targeting 720 human genes annotated to be kinases and phosphatases (four pooled siRNA oligos per gene, G-003705 Human Phosphatase Lot 09126 and G-003505 Human Protein Kinase Lot 09174, On-Target Plus, Dharmacon). siRNA oligos of a negative, nontargeting control siRNA (Negative siRNA Control Pool #2, Dharmacon, D-001206-14-20), a positive control siRNA pool (PLK1, Dharmacon, M-003290-01), and two positive biological controls of siRNA pools (PRKCE, Dharmacon; SKA3, customer synthesized) at the same concentration were included in eight replicates for each plate. Two microliters per well of siRNA of each oligo pool at the concentration of 1.25 µM were spotted at the bottom of 384-well CellCarrier Ultra imaging plate (PerkinElmer 6057300) using a PerkinElmer Janus Automated liquid handler. The 384-well plates were air dried under a sterile laminar flow for at least 30 min, sealed, and then stored at −80°C until transfection.

### siRNA oligo library transfection

On the day of transfection, the plates were equilibrated for at least 30 min at RT and then spun at 4000 rpm for 2 min. Twenty-five microliters of Opti-MEM (Thermo Fisher Scientific 51985034) containing 0.075 µL of premixed Lipofectamine RNAiMAX (Thermo Fisher Scientific 13778150) was dispensed in each well of the imaging plate using Thermo Fisher Scientific Multidrop Combi and incubated for 30 min at RT (Supplemental Fig. S14). Twenty-five microliters of 450 cells (18 cells/µL) in DMEM, 20% FBS were added to the siRNA oligo/RNAiMax mix, incubated for 30 min at RT and then incubated for 96 h at 37°C. The final concentration of siRNA oligos in the medium was 50 nM.

### Fixation and fluorescence staining

Cells were fixed by adding 50 µL of 8% paraformaldehyde (PFA) directly to the media, incubated for 15 min at RT, washed three times with 50 µL of PBS, and then incubated with 50 µL of DAPI (0.1 µg/mL) in PBS at 4°C until imaging.

### High-throughput imaging

Fixed and stained plates were imaged using a Yokogawa CV7000S spinning disk confocal microscope with Olympus 40× (NA 0.95) PlanApoChromat lens, an emission 405/488/561/640 dichroic mirror, and a 16-bit sCMOS camera (2550 × 2160 pixels) with pixel binning set to 2 × 2. For the DAPI channel, a 405-nm laser source and a 445/45-nm bandpass acquisition filter were used. For the GFP channel, a 488-nm laser source and a 525/50-nm bandpass acquisition filter were used. The DAPI and GFP channels were acquired sequentially at a single focal plane in nine fields of view per well. Images were saved as 16-bit TIFF files.

### High-content image analysis

TIFF files generated by the CV7000S microscope were imported and analyzed using PerkinElmer Columbus 2.7. The DAPI channel was used to segment a nuclear ROI mask, which was then used to measure the mean fluorescence intensity in the nucleus in the GFP channel. Nuclei touching the image borders and nuclei with a roundness value <0.7, often representing nuclear segmentation errors, were excluded from the subsequent analysis steps. The cells with values of GFP mean fluorescence intensity <100 AU, an empirically determined threshold that was kept constant for all plates in the screen, were classified as GFP−. The percentage of GFP− cells was used as a proxy for measuring HAC loss. Well-level data were exported as tab-separated text files.

### Calculation of doubling time of the HAC/dGFP-containing HT1080 cells

HT1080 cells containing HAC/dGFP cells (6500 cells/cm^2^) were seeded in a six-well tissue culture plate and cultivated in the presence of 10 µg/mL blasticidin for 210 h using Cell-IQ high-content in vivo imaging system equipped with 20× LUCPlanFLN Olympus Objective and Hamamatsu CCD camera. The growth curve was performed by time-lapse cell population analysis, recognizing each cell by its peculiar image using a phase-contrast microscopy using computer vision as well as fluorescent signal analysis to identify GFP-positive cells. The growth curve was generated automatically using Cell-IQ Analyzer software after the image library was performed, and each cell was marked with a specific dot marker plotted on the image mask for an operator's visual control (Supplemental Fig. S15).

### Calculation of the rate of HAC loss induced by siRNA-mediated knockdown of a target gene

The rate of HAC loss was calculated as previously described (Lee et al. 2013a) with some modifications (Supplemental Methods; Supplemental Table S9).

### Cytokinesis-block micronucleus assay

Cytokinesis-block micronucleus assay was performed as described (Fenech 2007) with minor changes (Supplemental Methods).

## Supplementary Material

Supplemental Material
