# Supplementary material for: A novel assay to screen siRNA libraries identifies protein kinases required for chromosome transmission
Source: Genome Res. 2019 Oct;29(10):1719–32. doi: 10.1101/gr.254276.119 (PMC6771407; doi:10.1101/gr.254276.119)
Supplement: Supplemental Material [file supp_29_10_1719__index.html]

A novel assay to screen siRNA libraries identifies protein kinases required for chromosome transmission — Supplemental Material 

# A novel assay to screen siRNA libraries identifies protein kinases required for chromosome transmission

## Supplemental Material

- Supplemental\_Material.docx
- Supplemental\_Movie\_S1.mp4
- Supplemental\_Movie\_S2.mp4
- Supplemental\_Movie\_S3\_PINK1.avi
- Supplemental\_Movie\_S4\_TRIO.avi
- Supplemental\_Movie\_S5\_IRAK1.avi
- Supplemental\_Movie\_S6\_PNCK.avi
- Supplemental\_Movie\_S7\_TAOK1.avi
- Supplemental\_Movie\_S8\_STK38.avi
- Supplemental\_Movie\_S9\_NC.avi
