# Supplementary material for: A novel assay to screen siRNA libraries identifies protein kinases required for chromosome transmission
Source: Genome Res. 2019 Oct;29(10):1719–32. doi: 10.1101/gr.254276.119 (PMC6771407; doi:10.1101/gr.254276.119)
Supplement: Supplemental Material [file supp_gr.254276.119_Supplemental_Material.docx]

**Supplemental Materials**

for

**A novel assay to screen siRNA libraries identifies protein kinases as required for chromosome transmission**

Mikhail Liskovykh^1,7^ , Nikolay V. Goncharov^1,2,7^, Nikolai Petrov^1^, Vasilisa Aksenova^3^, Gianluca Pegoraro^4^, Laurent L. Ozbun^4^, William C. Reinhold^1^, Sudhir Varma^1^, Mary Dasso^3^, Vadim Kumeiko^2^, Hiroshi Masumoto^5^, William C. Earnshaw^6^, Vladimir Larionov^1^ and Natalay Kouprina^1^

^1^Developmental Therapeutics Branch, National Cancer Institute, NIH, Bethesda, MD 20892, USA

^2^School of Biomedicine, Far Eastern Federal University, National Scientific Center of Marine Biology, Far Eastern Branch of RAS, Vladivostok, 690000, Russia

^3^Division of Molecular and Cellular Biology, National Institute for Child Health and Human Development, NIH, Bethesda, MD 20892, USA

^4^High-Throughput Imaging Facility, National Cancer Institute, NIH, Bethesda, MD 20892, USA

^5^Laboratory of Chromosome Engineering, Department of Frontier Research and Development, Kazusa DNA Research Institute, Kisarazu, Chiba 292-0818d, Japan

^6^Wellcome Centre for Cell Biology, University of Edinburgh, Edinburgh EH9 3JR, Scotland

^7^ These authors contributed equally.

Corresponding authors: mikhail.liskovykh@nih.gov; larionov@mail.nih.gov; kouprinn@mail.nih.gov

**Contents:**

1. Supplemental Figures S1-S15
2. Supplemental Tables S1-S9
3. Supplemental Methods
4. Supplemental Movies S1-S9

**Supplemental Figures**


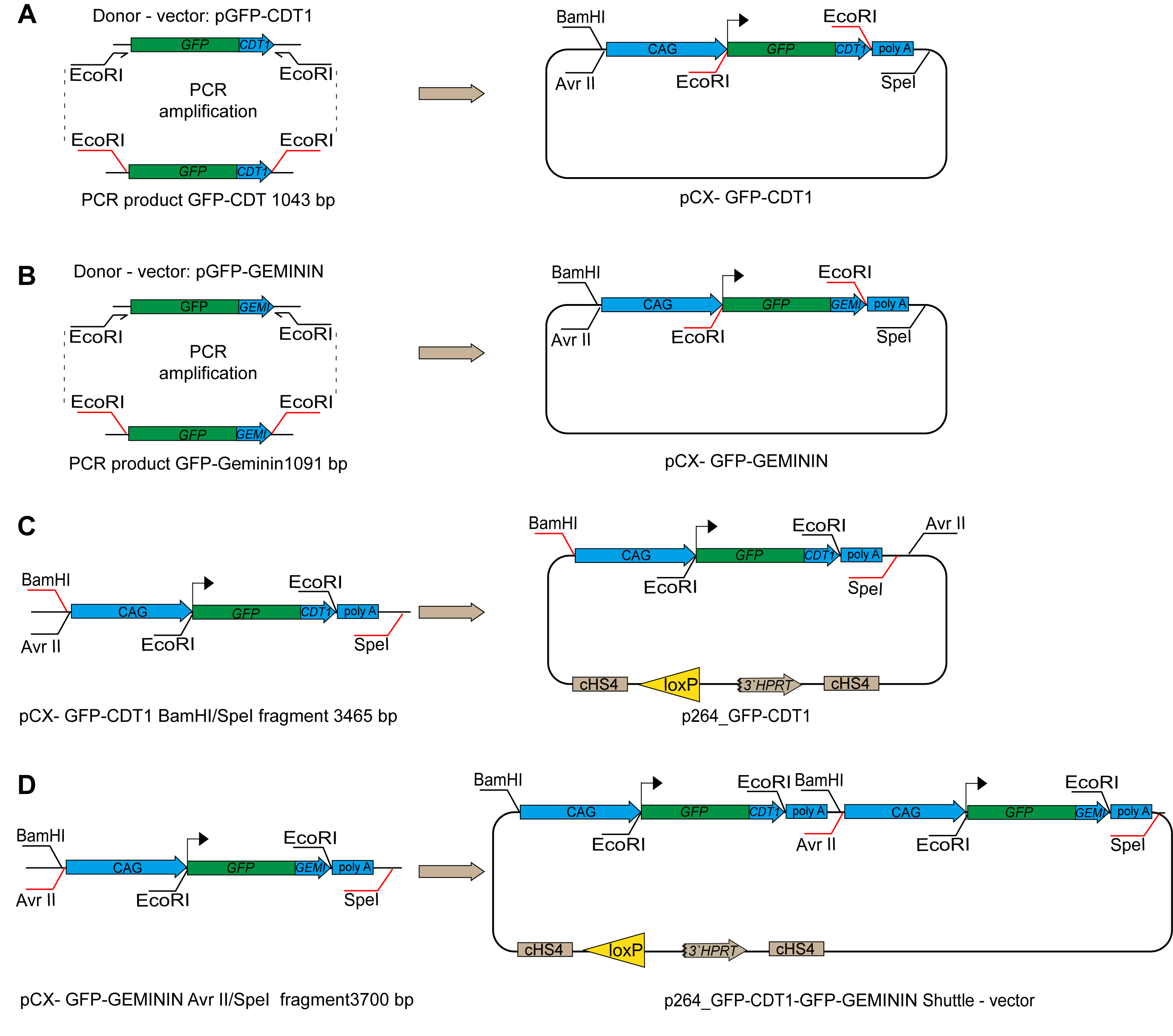


**Figure S1.** Construction of the p264_GFP-CDT1-GFP-GEMININ vector pCX-GFP-CDT1. (*A*) and pCX-GFP-GEMININ (*B*) vectors were constructed as follows. First, *GFP-CDT1* and *GFP-GEMININ* fusions were PCR-amplified from the *GFP-CDT1* and *GFP-GEMININ* synthetized gBlocks (IDT, USA) using the corresponding primers (Supplementary Table S6) with EcoRI restriction sites at 5’ends of 1,043 bp *GFP-CDT1* (*A*) and 1,091 bp *GFP-GEMININ* fragments (*B*). Then EcoRI/EcoRI fragments from the pGFP-CDT1 and pGFP- GEMININ vectors containing the cell cycle sensors and the GFP reporter were cloned into the EcoRI digested pCX vector producing the pCX-GFP-CDT1 and pCX-GFP-GEMININ vectors. (*C*, *D*) p264_GFP-CDT1- GFP-GEMININ vector was constructed as follows. A 3,465 bp BamHI/SpeI fragment from the pCX- GFP-CDT1 vector contains the *GFP-CDT1* fusion under the CAG promotor. This fusion was cloned into the p264 vector (Lee at al. 2013b) producing the p264_GFP-CDT1 vector (**c**). A 3,700 bp AvrII/SpeI fragment from the pCX-GFP-GEMININ vector was cloned into the p264_GFP-CDT1 Avr II-digested vector producing the p264_GFP-CDT1-GFP-GEMININ vector (*D*). A had to tail orientation of the *GFP-CDT1-GFP-GEMININ* sequences was chosen for the next steps. p264_GFP-CDT1-GFP-GEMININ vector has a single loxP site and a 3’part of the *HPRT* gene flanked by the cHS4 insulator that are essential for its loading into the alphoidtetO-HAC.

**
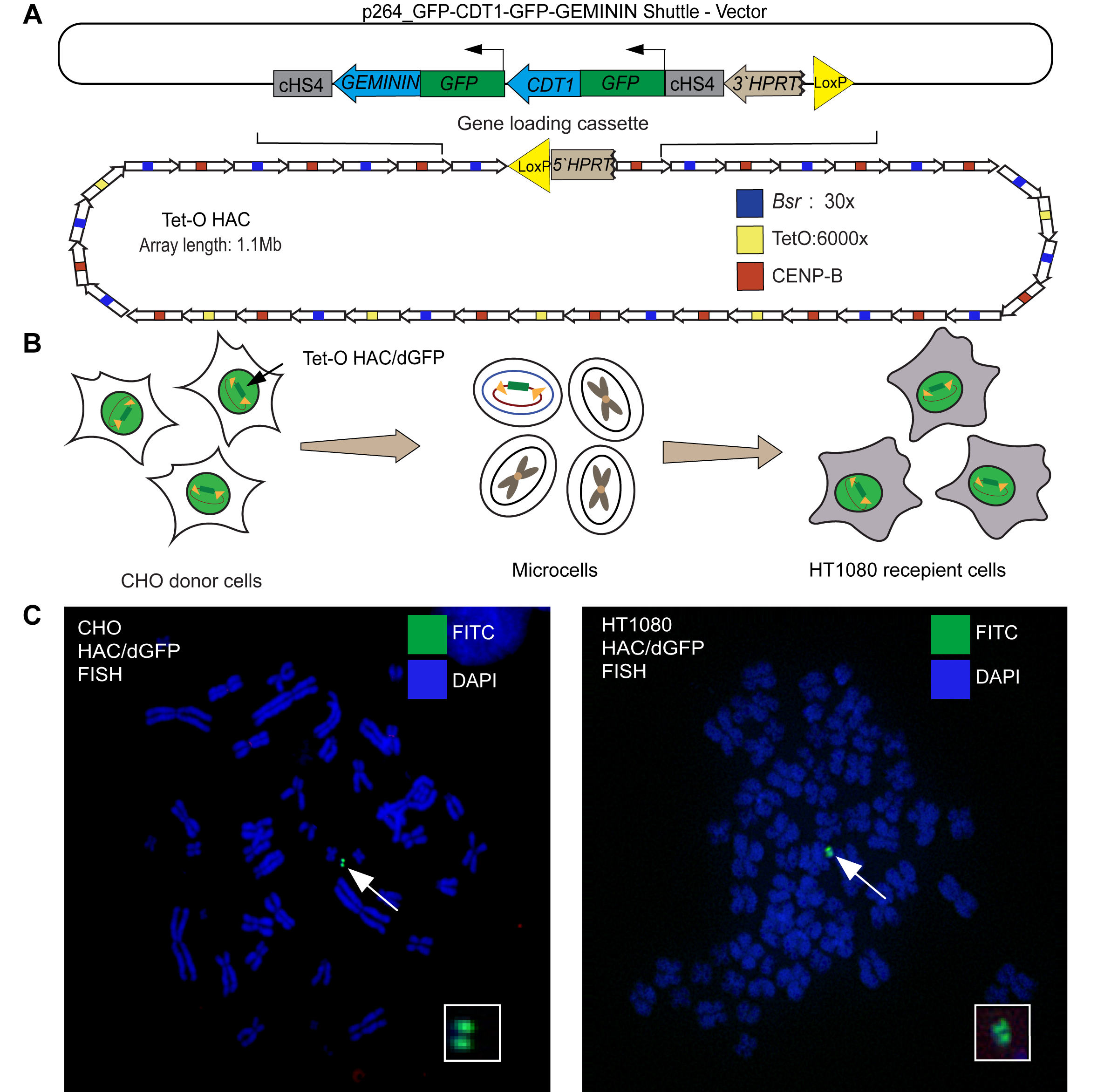
**

**Figure S2.** Loading of the p264_GFP-CDT1-GFP-GEMININ vector into alphoidtetO-HAC with the following MMCT transfer of the HAC/dGFP from hamster CHO cells to human HT1080 cells. (*A*)The *GEMININ-GFP-CDT1-GFP* cassette is protected by the cHS4 insulator to prevent epigenetic silencing of the transgene. The p264_GFP-CDT1-GFP-GEMININ vector was loaded into the loxP site of alphoidtetO-HAC (or Tet-O HAC) by Cre-loxP-mediated recombination followed by reconstitution of the *HPRT* gene producing the HAC/dGFP. The original alphoidtetO-HAC contains ~6,000 copies of the tetracycline operator (tetO) sequence and ~30 copies of the selectable marker blasticidin (*Bsr*) (Nakano et al. 2008). The size of the alphoid DNA array in the HAC is ~1.1 Mb. (*B*) MMCT transfer of the HAC/dGFP from the donor hamster CHO cells to human HT1080 recipient cells. (*C***)** FISH analysis of the HAC/dGFP in hamster CHO and human HT1080 cells using the PNA-labeled probe for the tetO sequences (in green). White arrows point to the HAC/dGFP. Chromosomal DNA was counterstained with DAPI (in blue).

**
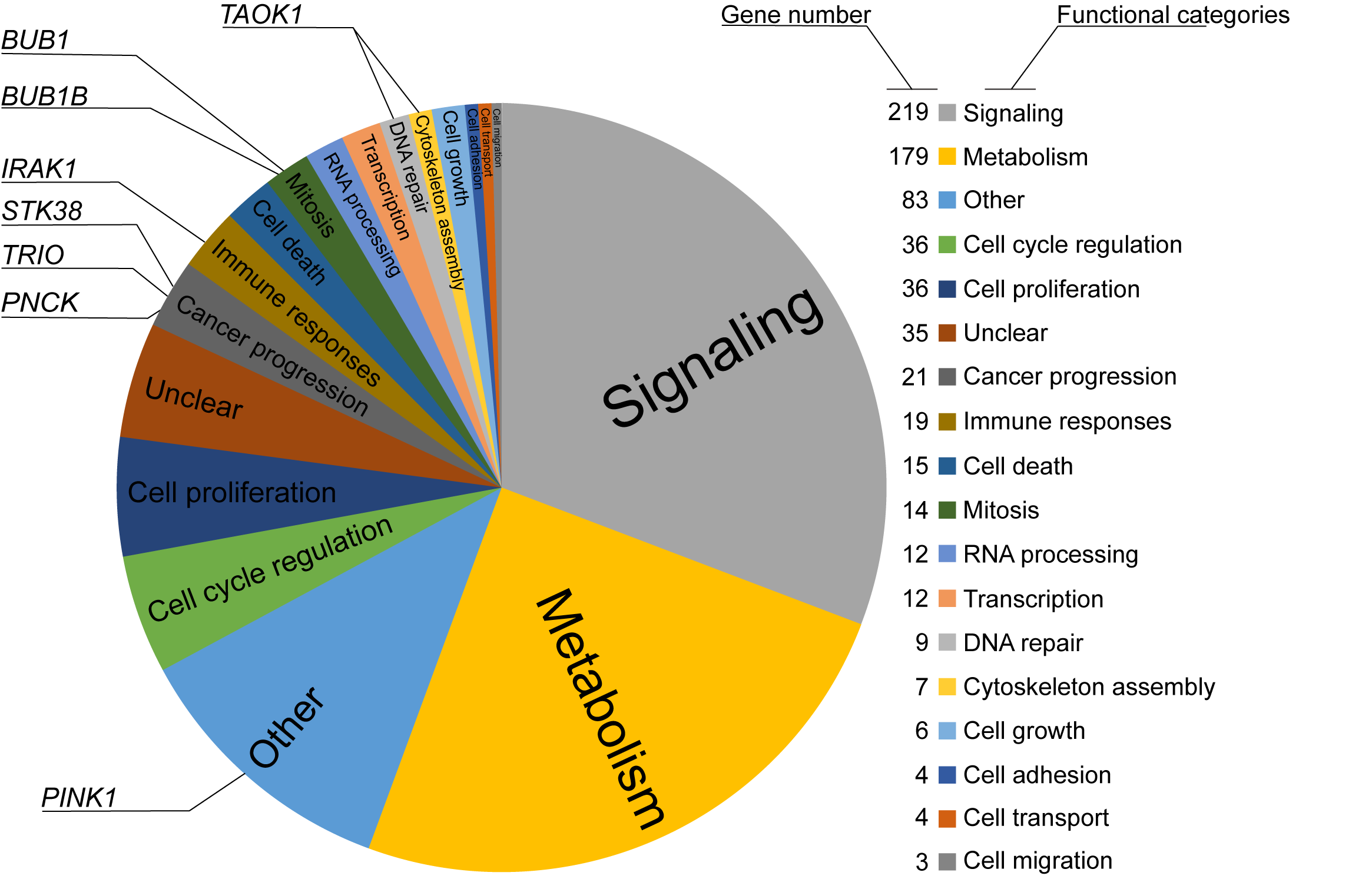
**

**Figure S3.** siRNA library contains 714 genes previously annotated as either kinases or phosphatases**.** The genes are grouped by functional categories according to their roles in basic biological processes. The majority of protein-kinases reveal activity as signaling messengers (in grey). The protein kinases that did not form a group were classified as “Other” (in blue). The proteins with the unknown biological role were classified as “Unclear” (in brown). Eight following genes identified by the HAC/dGFP-HTI assay effect chromosome stability. These genes were re-confirmed by individual siRNAs. TAOK1 is attributed to the regulators of DNA repair process and actin cytoskeleton assembling. BUB1 and BUB1B are the known compounds of spindle assembling checkpoint and mitosis regulation. IRAK1 is an interleukin 1 (IL-1) receptor-associated kinase that plays a critical role in initiating innate immune responses against foreign pathogens and other types of dangers. STK38, TRIO*,* and PNCK are associated with tumor progression. PINK1 is a mitochondrial signal protein which activates stress-inhibition of mitochondria.


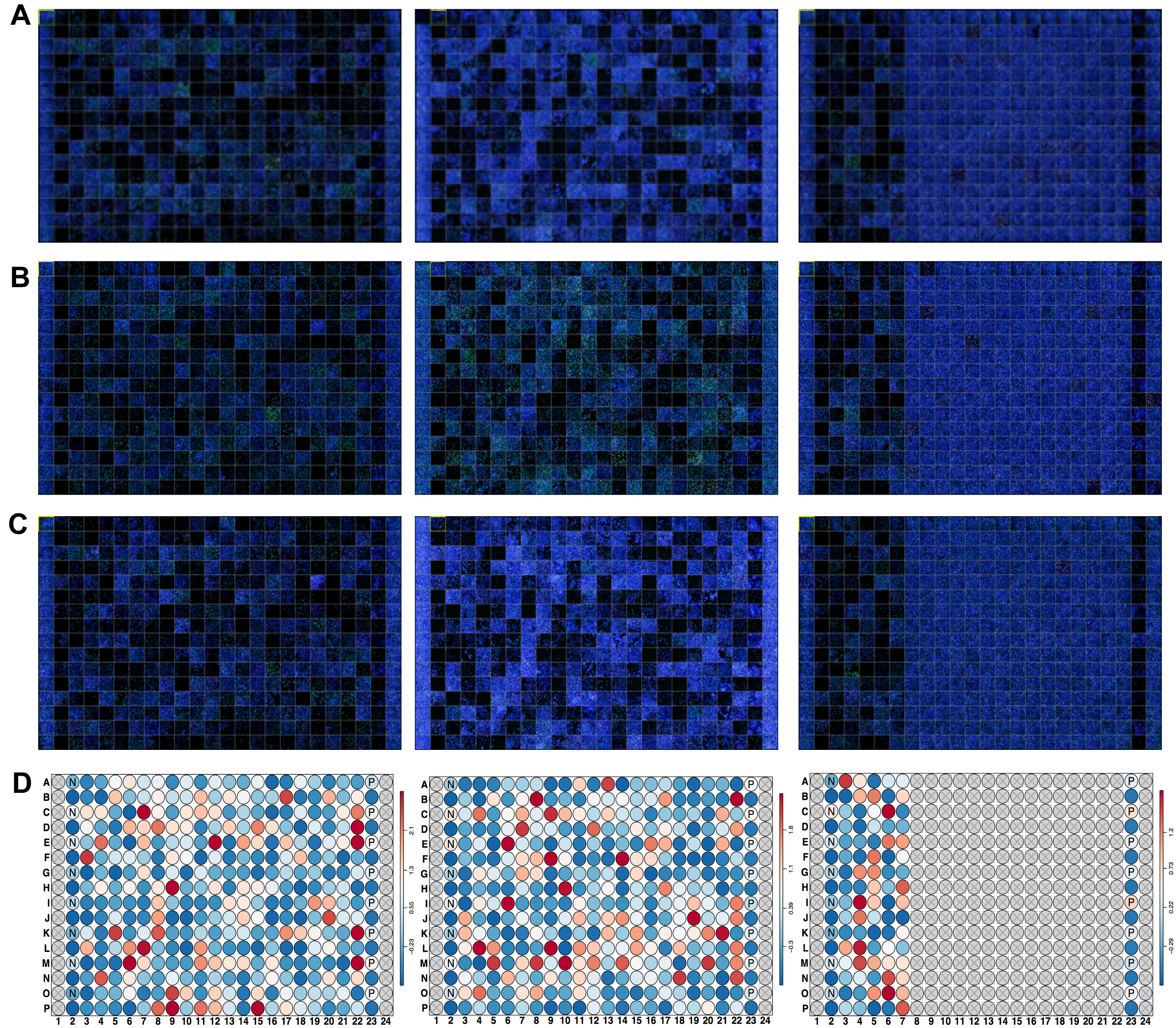


**Figure S4.** Confocal laser scanning fluorescence microscopy images of the protein kinases siRNA library screening and the heat-map data. (*A-C*) Three independent repeats of screening of siRNA library against protein kinases have been carried out. The pictures show representative 10-folf magnification of the confocal multiple images exported from Columbus Image Data Storage and Analysis System. Each repeat represents three 384-well plates with 714 genes analyzed. Each well (square) is a combination of 9 independent fields of a view. (*D*) GFP-positive cells heat map generated from three independent experiments normalized to a negative control (scr. siRNA). Knockdown of the genes is shown by color from blue to red reflecting the decrease of the GFP fluorescence intensity.


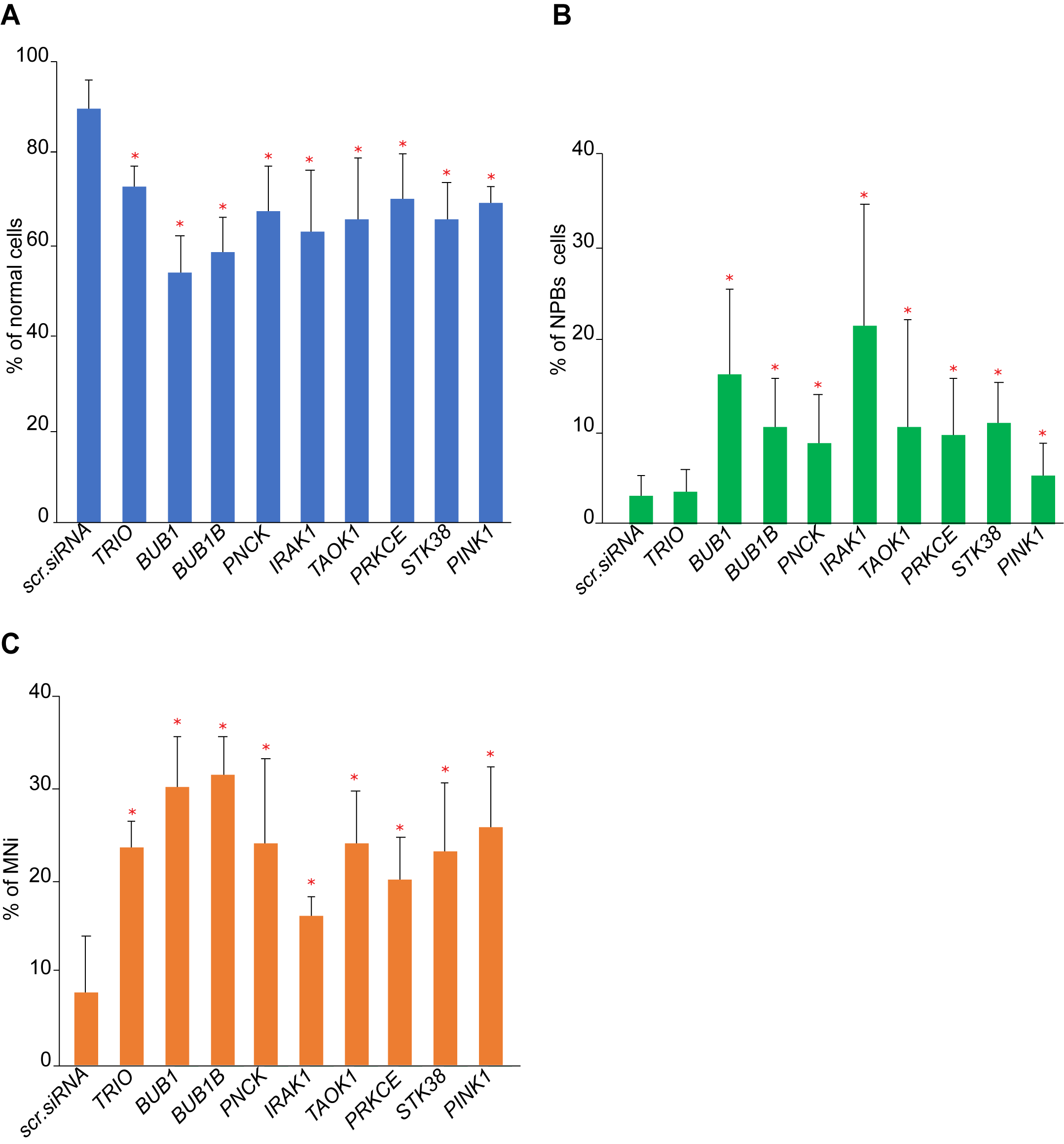


**Figure S5.** Micronuclei (MNi) and nucleoplasmic bridges (NPBs) formation in fibrosarcoma HT1080 cells after knockdown of one of the following genes: *TRIO, BUB1, BUB1B, PNCK, IRAK1, TAOK1,* *PRKCE, STK38* and *PINK1* genes. (*A*) The percentage of the binucleated cells without abnormalities. (*B*) The percentage of MNi formed after knockdown of the genes. (*C*) The percentage of NPBs after knockdown of the genes. Scrambled siRNA (scr. siRNA) was used as a negative control. Error bars correspond to a standard deviation (SD) of four replicates. The red asterisks indicates significant difference from a negative control, calculated by Fisher's exact test with Bonferroni correction for multiple testing (p<0.0011).

**
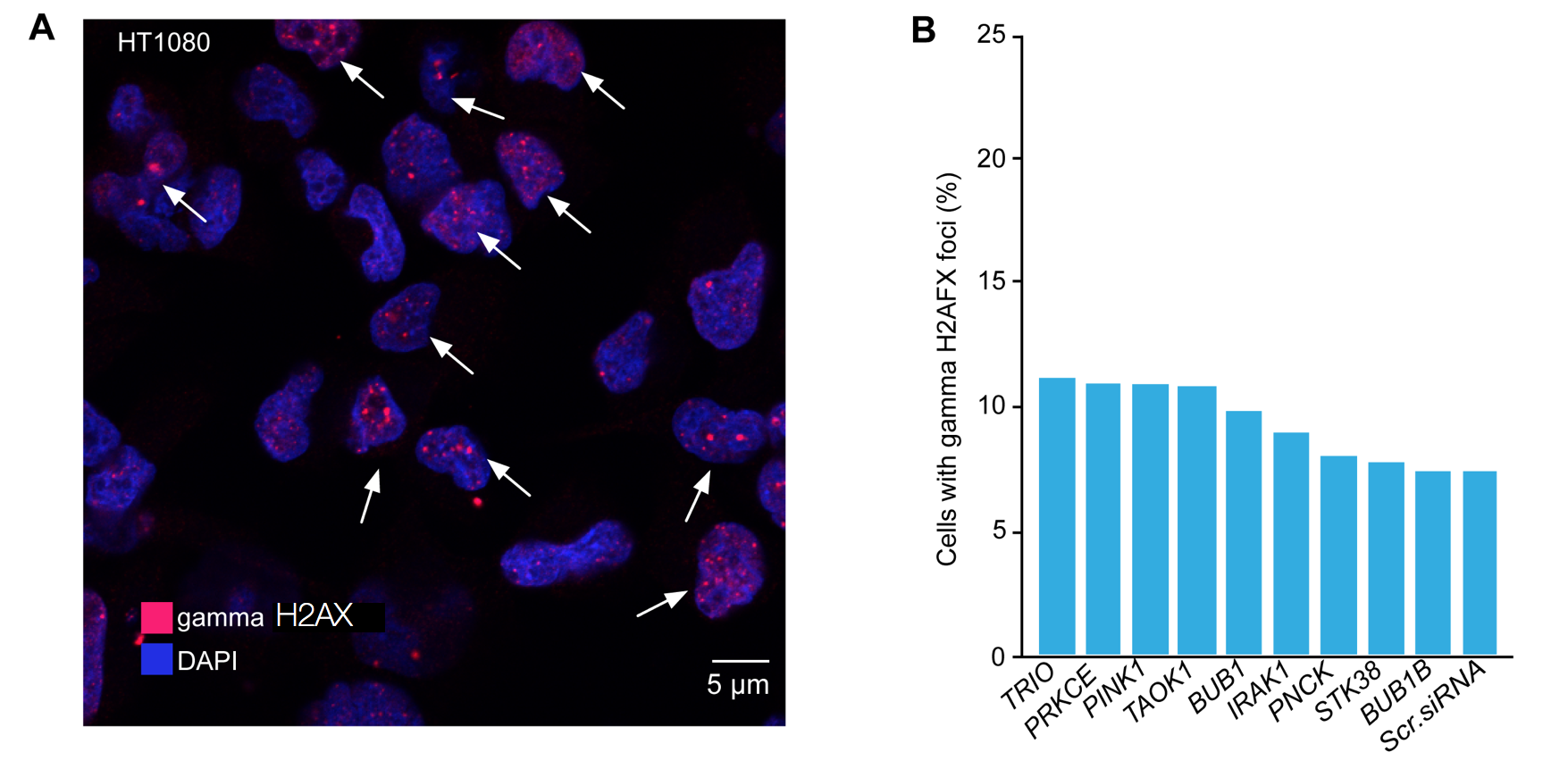
**

**Figure S6.** Immunostaining of double-stranded breaks (DSBs) with antibody against phosphorylated gamma H2AX in interphase of HT1080 cells after knockdown of *TRIO, PRKCE, PINK1, TAOK1, BUB1, IRAK1, PNCK, STK38,* and *BUB1B* genes. (*A*) Examples of immunostaining of the cells. Red signals – gamma H2AX staining as a marker for DSBs. Accumulation of gamma H2AX foci occurred at day 3. White arrows point to the cell nuclei with gamma H2AX signals. (*B*) A statistical effect of gamma H2AX foci was determined at day 3 (Fisher’s exact test: p-value; 2-tailed). As seen, the number of gamma H2AX foci in HT1080 cells almost didn’t change after siRNA knockdown of the listed genes that may be due to a high internal level of DNA damage in HT1080 cells. Even a negative control (scr. siRNA) shows a high level of foci that masks all other effects making them statistically insignificant.

**
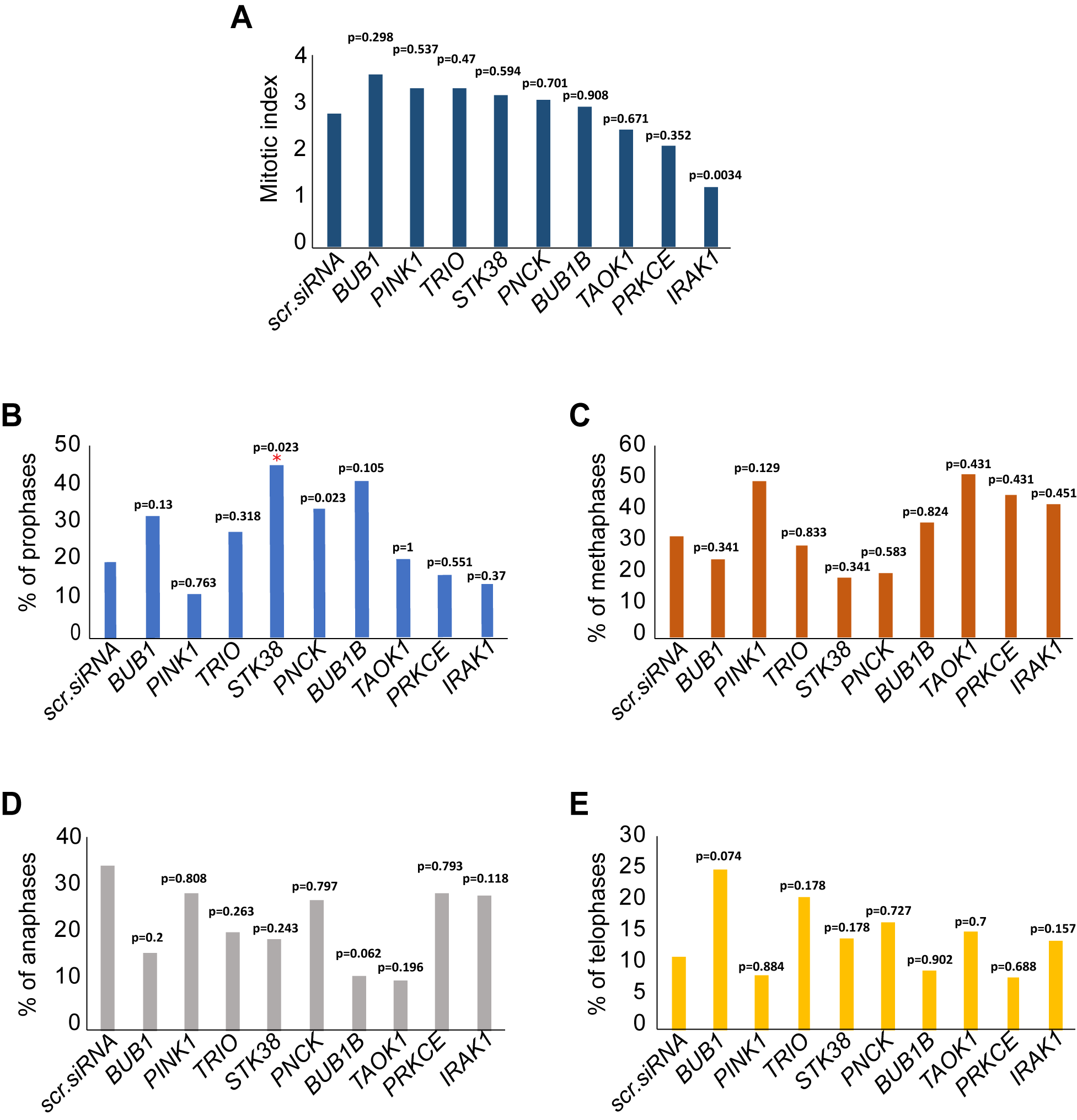
**

**Figure S7.** Mitotic index (*A*) and distribution of different stages of mitosis (*B-E*) in RPE cells after knockdown of *PINK1, STK38, PRKCE, TRIO, TAOK1, IRAK1, BUB1B, PNCK,* and *BUB1* genes. To count mitotic index, approximately 1,000 nuclei were analyzed. To count distribution of mitotic stages, approximately 50-60 mitotic events were analyzed. For statistical significance, Fisher’s exact test was applied. p-value <0.05 was considered as significant (red asterisk).


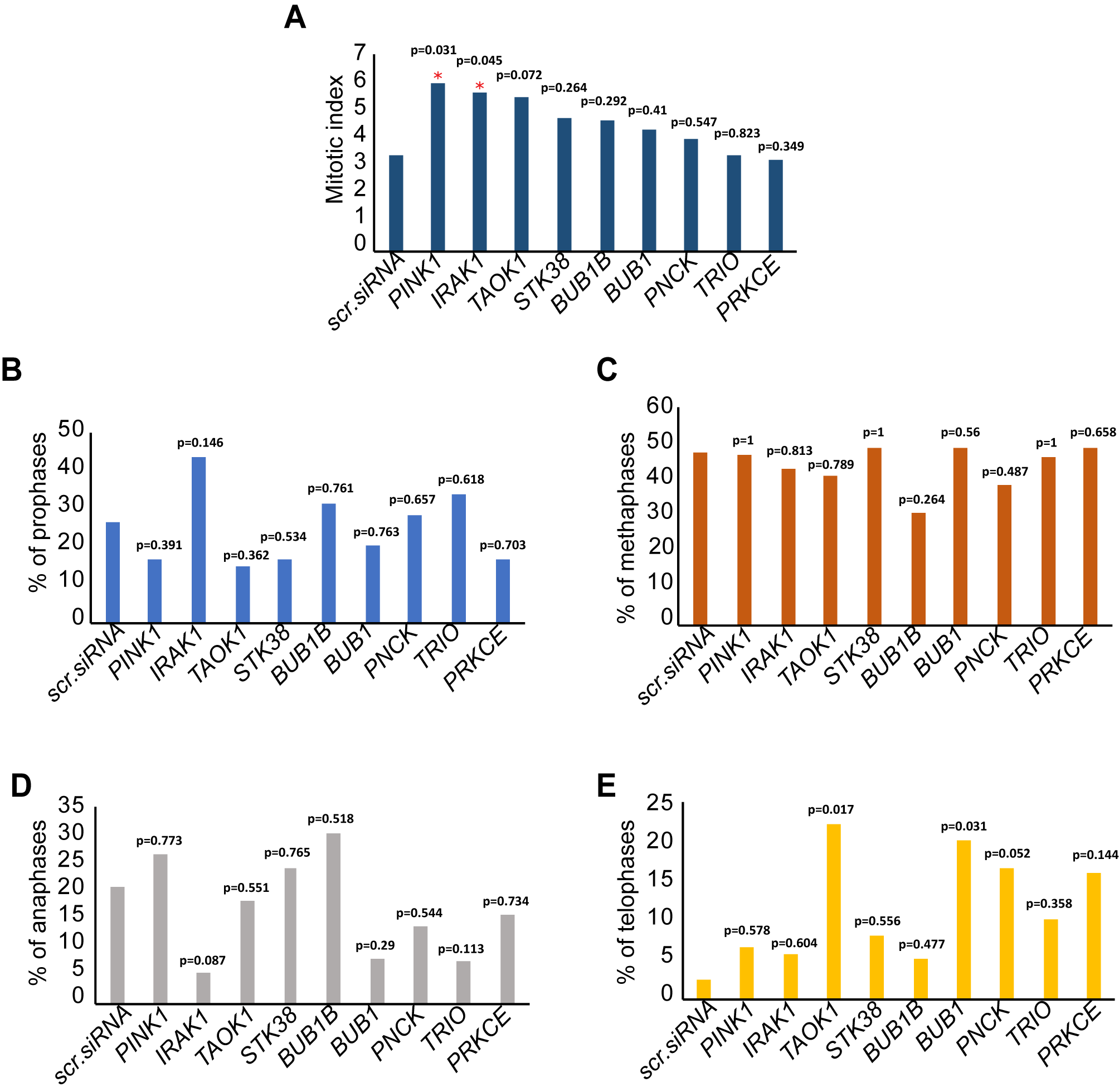


**Figure S8.** Mitotic index (*A*) and distribution of different stages of mitosis (*B-E*) in HT1080 cells after knockdown of *PINK1, STK38, PRKCE, TRIO, TAOK1, IRAK1, BUB1B, PNCK,* and *BUB1* genes. To count mitotic index, approximately 1,000 nuclei were analyzed. To count distribution of mitotic stages, approximately 50-60 mitotic events were analyzed. For statistical significance, Fisher’s exact test was applied. p-value <0.05 was considered as significant (red asterisks).

**
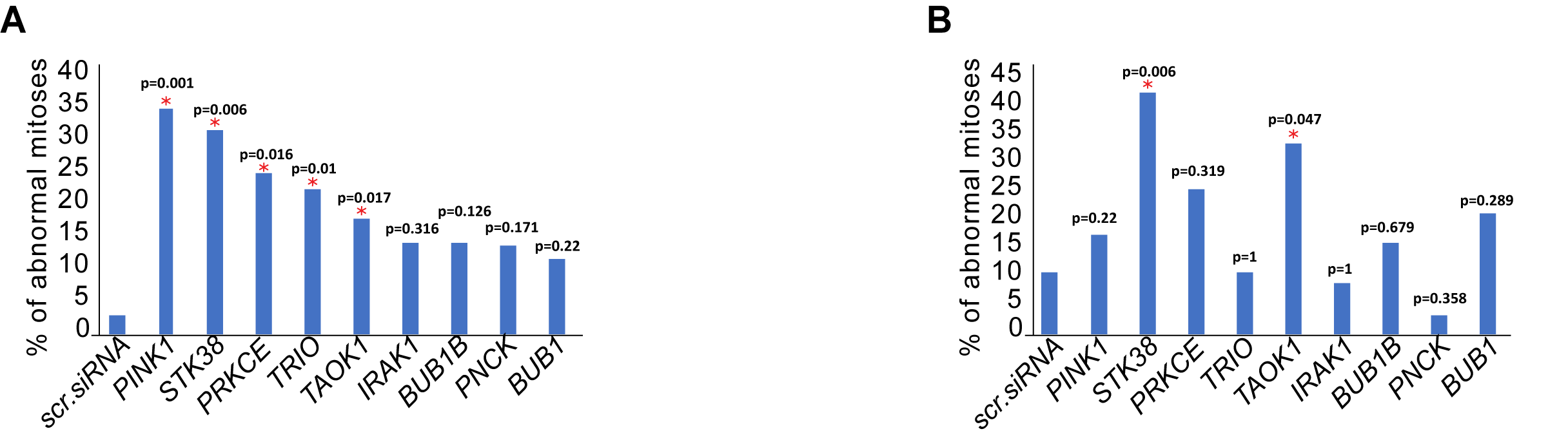
**

**Figure S9.** Proportion of the abnormal mitoses observed in RPE (*A*) and HT1080 (*B*) cells after knockdown of *PINK1, STK38, PRKCE, TRIO, TAOK1, IRAK1, BUB1B, PNCK,* and *BUB1* genes**.** For calculations, approximately 50-60 mitotic events were analyzed. For statistical significance, Fisher’s exact test was applied. p-value <0.05 was considered as significant (red asterisks).

**
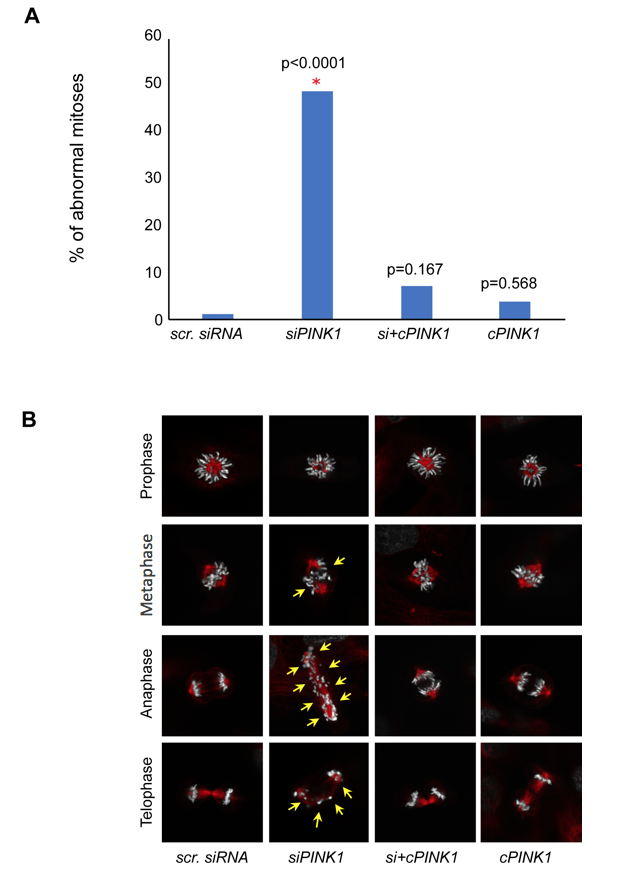
**

**Figure S10.** Results of the rescue experiment for the *PINK*1 gene. (*A*) Percentage of abnormal mitoses counted after transfection of RPE cells with scrambled siRNA (scr. siRNA); siRNA against *PINK1* (si*PINK1*); siRNA against *PINK1* together with cDNA resistant to siRNA (si+c*PINK1*); and cDNA of *PINK1* gene (c*PINK1*). For statistical significance Fisher’s exact test was applied. Red asterisk indicates statistical significance (p<0.05) in comparison with negative control. About 150 mitotic events were analyzed. (*B*) Immunostaining of transfected, as described above, RPE cells against tubulin alpha (red) counterstained with DAPI to observe mitotic abnormalities. Yellow arrows point to the identified mitotic abnormalities.

**
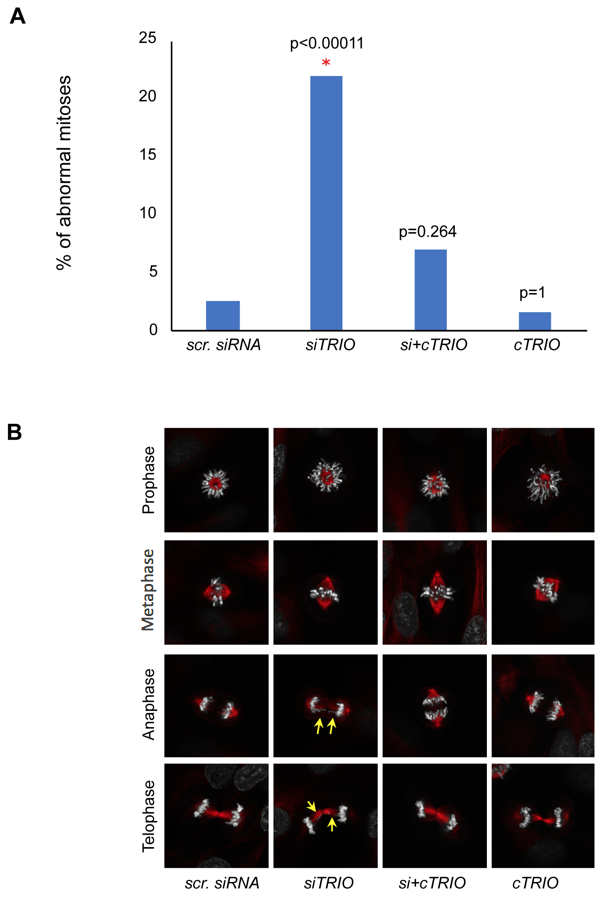
**

**Figure S11.** Results of the rescue experiment for the *TRIO* gene. (*A*) Percentage of abnormal mitoses counted after transfection of RPE cells with scrambled siRNA (scr. siRNA); siRNA against *TRIO* (*siTRIO*); siRNA against *PINK1* together with cDNA resistant to siRNA (si+*cTRIO*); and cDNA of *PINK1* gene (*cTRIO*). For statistical significance Fisher’s exact test was applied. Red asterisk indicates statistical significance (p<0.05) in comparison with negative control. About 150 mitotic events were analyzed. (*B*) Immunostaining of transfected, as described above, RPE cells against tubulin alpha(red) counterstained with DAPI to observe mitotic abnormalities. Yellow arrows point to the identified mitotic abnormalities.

**
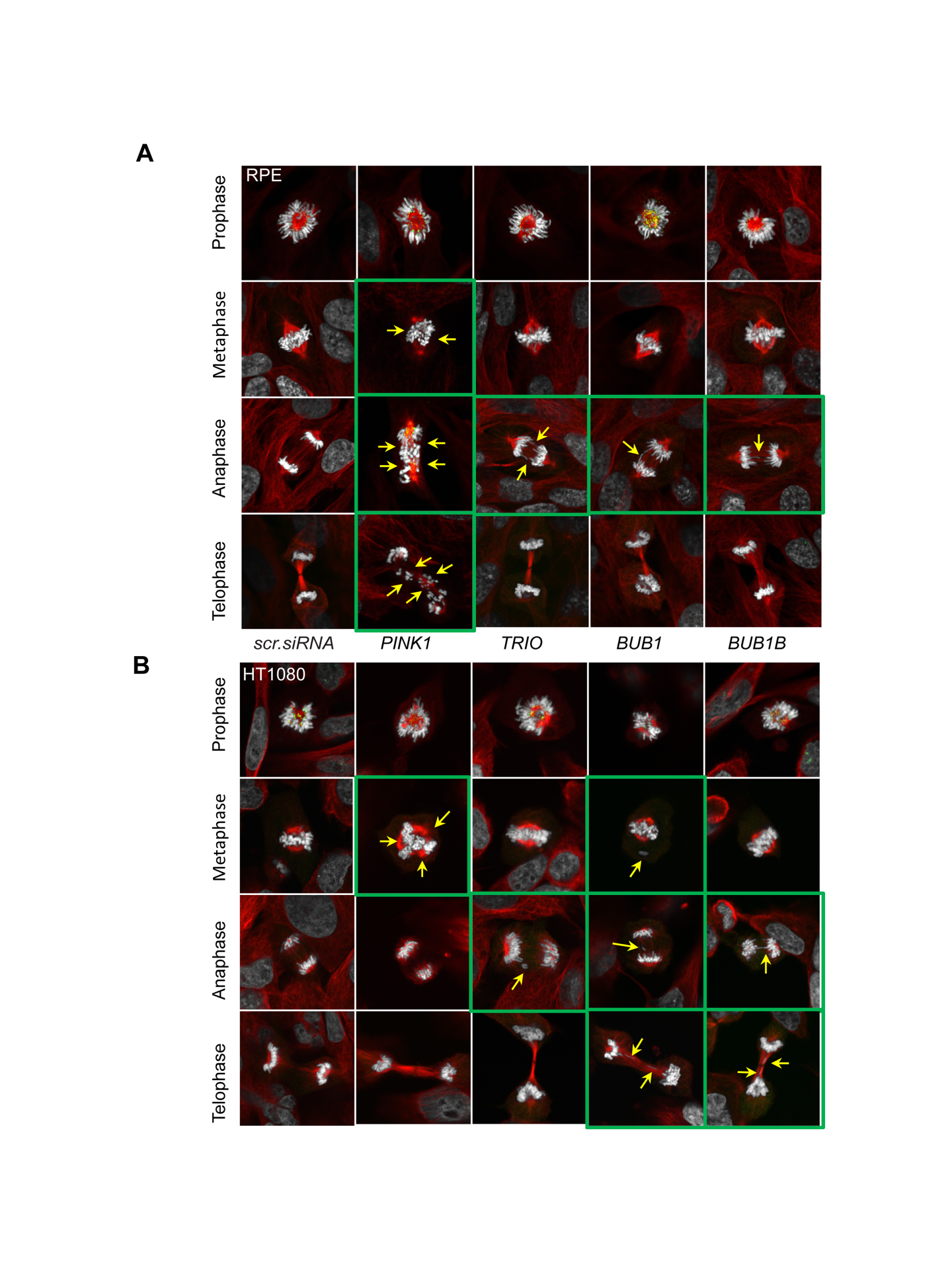
**

**Figure S12.** Analysis of localization of tubulin alpha and MAD1 at the different stages of mitosis after knockdown of *PINK1, TRIO, BUB1,* and *BUB1B* genes in RPE (*A*) and HT1080 (*B*) cells. scr. siRNA stands for a negative control. Staining by antibodies against tubulin alpha is marked in red, against MAD1 in green. DAPI counterstaining in grey. Green squares and yellow arrows point to the observed mitotic abnormalities.

**

Figure S13.** A gene interaction network map that represents potential functional relationships among the CIN discovered kinases (PINK1, STK38, TRIO, IRAK1, PNCK, and TAOK) and the proteins involved in cell division and cell cycle regulation. The most frequent relationship was protein-protein interactions (54), followed by activation (27) and phosphorylation (21) (Supplemental Table S6).

**
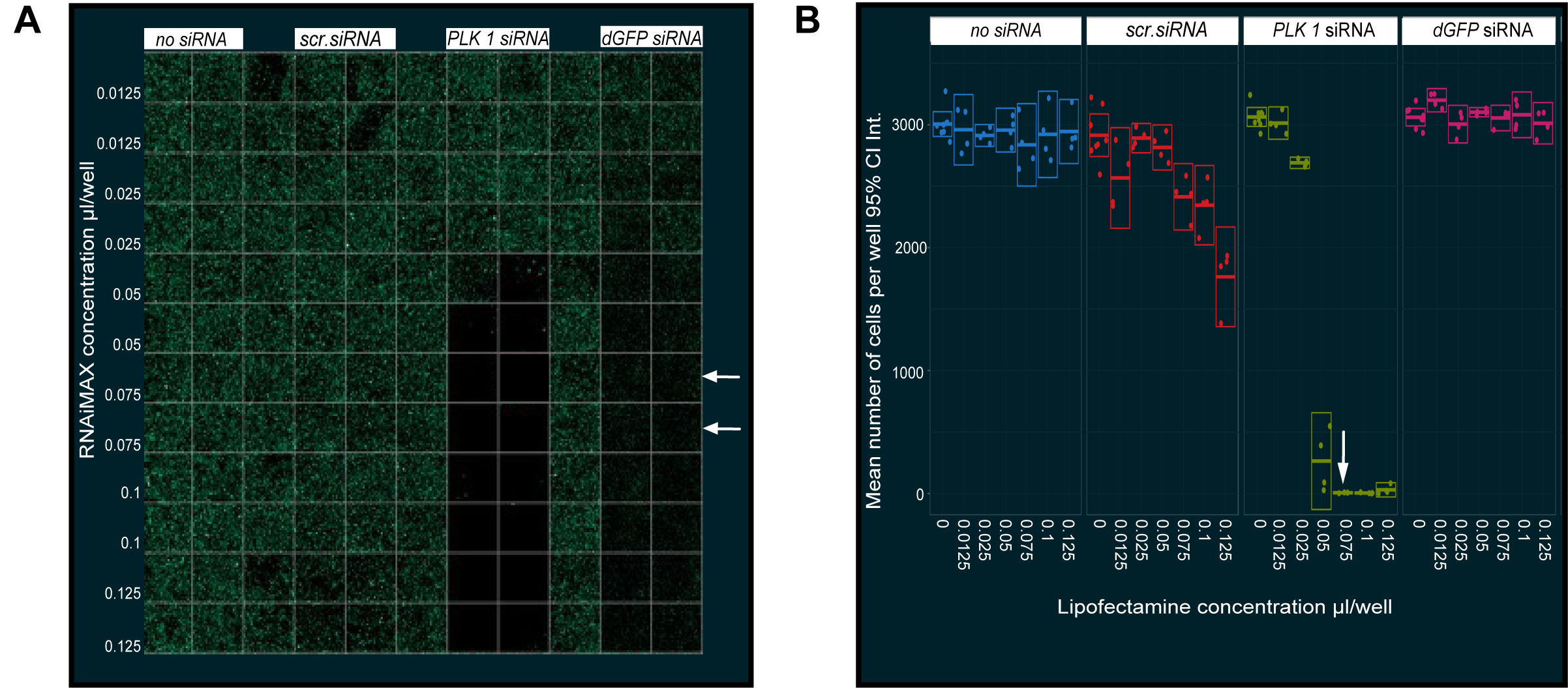
**

**Figure S14.** Optimization of siRNA transfections for high-content screening. Efficiency of transfection across wells was normalized for *PLK1.* HT1080 cells containing HAC/dGFP were transfected with siRNAs against *PLK1* and *dGFP* on 384-well plates with different concentrations of Lipofectamine RNAiMAX for 16 hrs. After 96 hrs, the total number of cells with the GFP signal was quantified. (*A*) Representative 10-fold magnification of confocal multiple images exported from Columbus Image Data Storage and Analysis System. Each square is a combination of nine independent fields of view. (*B)* Identification of the optimal Lipofectamine RNAiMAX concentration based on cell viability after siRNA against *PLK1* transfection and loss of the GFP signal caused by *dGFP* knockdown. The error bars represent the standard deviation of three independent repeats. Concentration of Lipofectamine RNAiMAX 0.075 μl per well was identified as optimal for the siRNA library screening.


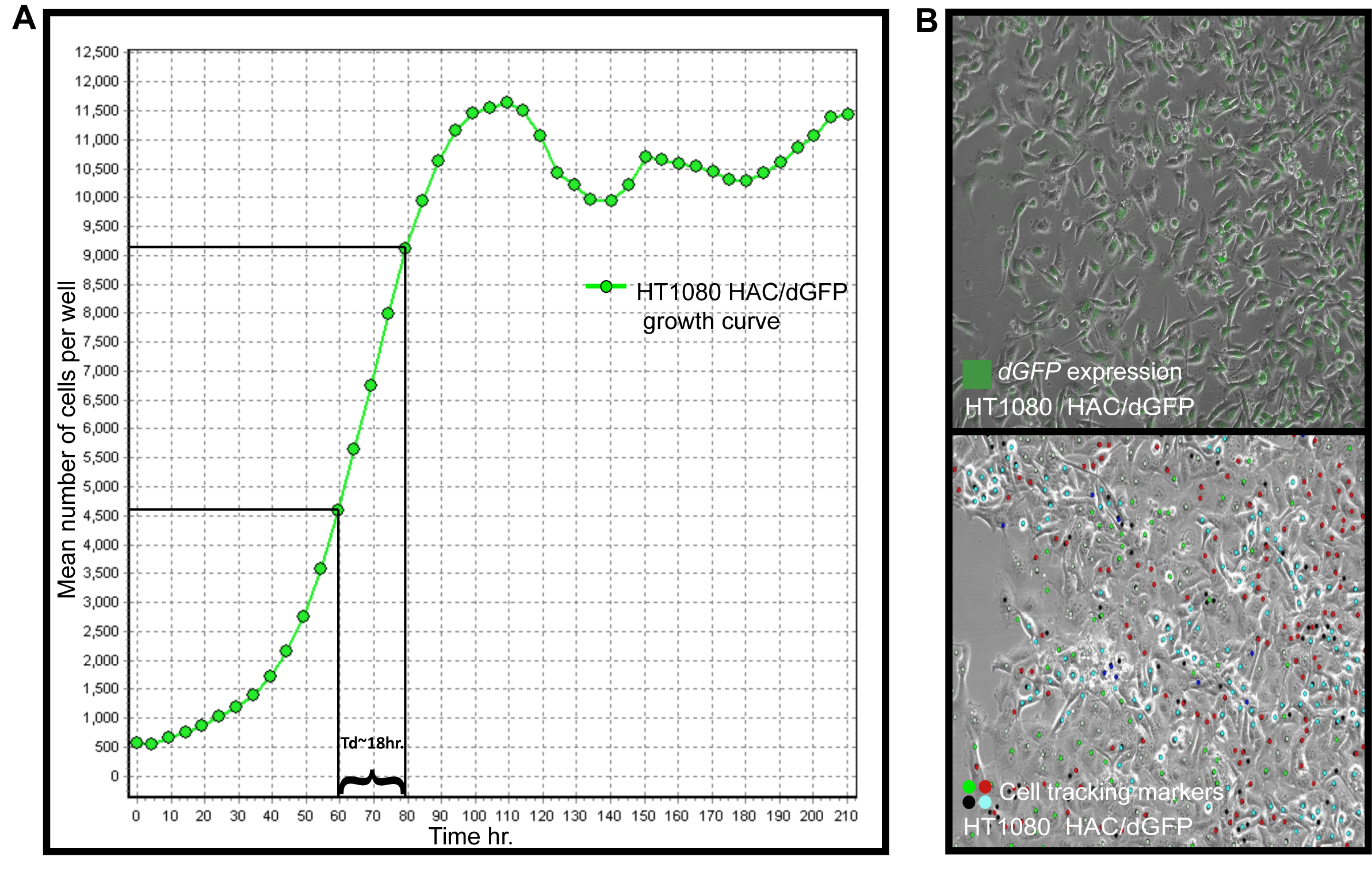


**Figure S15.** Calculation of doubling time in HAC/dGFP-containing HT1080 cells. (*A*) Doubling time (Td) was calculated in the logarithmic phase of the growth curve of the HAC/dGFP-containing HT1080 cells. One average cell cycle of HAC/dGFP-containing HT1080 cells takes approximately 18 hours. (*B*) The pictures of HAC/dGFP-containing HT1080 cells expressing the *dGFP* transgene (top) and the cell tracking markers of different subtypes of the events (bottom): GFP-positive cells, cell debris, non-fluorescence cells, and dead cells. The growth curve was developed using cell tracking of the GFP-positive cells and a recorded growth of cell population at multiple time points excluding the death cells, cell debris and non-fluorescence cells from the final data set.

**Supplemental Tables**

**Table S1 A list of siRNAs used in this study**

|  | **siRNA target** | **Gene ID** | **Sense sequence as ordered***** |
| --- | --- | --- | --- |
|  | **Control human CIN genes** | | |
|  | *SKA3* | 221150 | 5′- AGACAAACAUGAACAUUAAUU -3′ |
|  | *CENPE* | 1062 | 5′- AACACGGAUGCUGGUGACCUC -3′ |
|  | *OIP5* | 11339 | 5′- AGGCAGUACUUACAACCUUUU -3′ |
|  | *AURKB* | 9212 | 5′-GGUGAUGGAGAAUAGCAGUdTdT-3′ |
|  | *CENPN* | 55839 | 5′- CUACCUACGUGGUGUACUAUU -3′ |
|  | *CENPA* | 1058 | 5′-GCCCGAGGCCCCGAGGAGGUU-3′ |
|  | **Human orthologs of yeast CIN genes** | | |
|  | *RPL13* | 6137 | 5′- CACUGAGGAAGAGAAGAAUUUCAAA-3′ |
|  | *CNOT6* | 57472 | 5′- UUCUUUUCAGACUUGUUGG-3′ |
|  | *NF1* | 4763 | 5'-CAGTGAACGTAAGGGTTCT-3′ |
|  | *PIGB* | 9488 | 5'- GAAAUAAGCGCUUUCCUAAUUUCdGdC-3' |
|  | *MUC4* | 4585 | 5′-CAGCGACACTAGAGGGACAUU-3′ |
|  | *PRC1* | 9055 | 5′- GGG AUU CCA GAG GAC CA AA-3′ |
|  | *MYO5B* | 4645 | 5′- GUGCCAGUCUAAAGAUGAAUU-3′ |
|  | *PRKCE* | 5581 | 5′-AAGCCCCTAAAGACAATGAAG-3′dTdT-3′ |
|  | *MSI1* | 4440 | 5′-GGAGAAAGUGUGUGAAAUUdTdT3-3′ |
|  | *SMARCAD1* | 56916 | 5′- GCATGAACCCCTTGTGCTG-3′ |
|  | *XAB2* | 56949 | 5′- GAACCAAUUCUCUGUCAAAdTdT-3′ |
|  | *PIGU* | 128869 | Dharmacon# J-017428-05-0002 |
|  | *NPEPPS* | 9520 | Dharmacon# J-005979-12-00 |
|  | *PIGS* | 94005 | Dharmacon# J-013702-12-0002 |
|  | *CIAO2B* | 51647 | Dharmacon# J-020340-12-0002 |
|  | *RTN2* | 6253 | Dharmacon# J-012717-20-0002 |
|  | *C12orf10* | 60314 | Dharmacon# J-013747-20-0002 |
|  | *MEMO1* | 51072 | Dharmacon# J-016087-20-0002 |
|  | *GPN2* | 54707 | Dharmacon# J-020288-12-0002 |
|  | *TANGO6* | 79613 | Dharmacon# J-034978-19-0002 |
|  | *WDR76* | 79968 | Dharmacon# J-014509-08-0002 |
|  | *UAP1* | 6675 | Dharmacon# J-017160-09-0002 |
|  | *RAB1A* | 5861 | 5′-CAGCAUGAAUCCCGAAUAU– 3’ |
|  | *PLCD3* | 113026 | 5′-UGAACGACAUGUACGCCUA– 3’ |
|  | *PPIP5K1* | 9677 | 5′-GUAUUUGCCCUGAUCGAAA– 3’ |
|  | *IPO11* | 51194 | 5′-GACGGAAGAUCCUGAAACA– 3’ |
|  | *NAT10* | 55226 | 5′-GGAAUAUGGUGGACUAUCA– 3’ |
|  | *AP2B1* | 163 | 5′-GUACAAUGAUCCCAUCUAU– 3’ |
|  | **The genes reconfirmed by individual siRNAs** | | |
|  | *MAPK7* | 5598 | 5′-CACGACAACAUCAUCGCCAUU– 3’ |
|  | *IRAK1* | 3654 | 5′-AAGUUGCCAUCCUCAGCCUCC– 3’ |
|  | *BTK* | 695 | 5′-GCCAAUGAAUGCCAAAUGAUdtdt– 3’ |
|  | *TAOK1* | 57551 | 5′-CCAACUAUCUCGUCACAAAUU– 3’ |
|  | *TNK2* | 10188 | 5′-GGUGUUCAGUGGAAAGCGA– 3’ |
|  | *NEK9* | 91754 | 5′-GGACUCAAUGAAUUCAAUA– 3’ |
|  | *TTBK1* | 84630 | 5′-GAACAGGUAGGGAUGAUCA– 3’ |
|  | *CSNK1G2* | 1455 | 5′-UCGAGAAGCCCGCCUAUGA– 3’ |
|  | *CRIM1* | 51232 | 5′-GAACUGGACUGAUGACCAA– 3’ |
|  | *TRIO* | 7204 | 5′-AAAAAUGCCUAUGUUCAACCA– 3’ |
|  | *HIPK2* | 28996 | 5′-GAGAAUCACUCCAAUCGAA– 3’ |
|  | *STK11* | 6794 | 5′-UGACUGUGGUGCCGUACUU– 3’ |
|  | *ITPKB* | 3707 | 5′-GAAGUGGCAGCGAGAGUUA– 3’ |
|  | *MYLK* | 4638 | 5′-CUAAGACCAUUCGCGAUUU– 3’ |
|  | *CSK* | 1445 | 5′-CUGGCCAUCCGGUACAGAAUU– 3’ |
|  | *STK38* | 11329 | 5′-CCUUAUCGCUCAACAUGAAdtdt– 3’ |
|  | *PNCK* | 13972 | 5′-AGAACGAGAUCGCAGUGCUUU– 3’ |
|  | *PINK1* | 65018 | 5′-AUGGGUCAGCACGUUCAGUUA– 3’ |
|  | **Negative control** | | |
|  | ON-TAGET scr.siRNA pool |  | 5′- UGGUUUACAUGUCGACUAA -3′  5′- UGGUUUACAUGUUGUGUGA -3′  5′- UGGUUUACAUGUUUUCUGA -3′  5′- UGGUUUACAUGUUUUCCUA -3′ |
|  | Dharmacon siGENOME® SMARTpool® siRNA Library of Human Protein Kinases |  |  |

**Table S2 A final list of novel CIN genes**

| **Gene name and**  **Gene ID** | **Corresponding references** |
| --- | --- |
| ***BUB1***  **Gene ID: 699** | 1. (Asghar et al. 2015) 2. (Breit et al. 2015) 3. (Cahill et al. 1998) 4. (Gao et al. 2009) 5. (Jessulat et al. 2015) 6. (Jia, Li & Yu 2016) 7. (Musio et al. 2003) 8. (Nilsson 2015) 9. (Overlack et al. 2015) 10. (Ricke, Jeganathan & van Deursen 2011) 11. (Wills et al. 2016) |
| ***BUB1B***  **Gene ID: 701** | 1. (Chou et al. 2015) 2. (Hahn et al. 2016) 3. (Mansouri et al. 2016) 4. (Weaver et al. 2003) 5. (Weaver et al. 2016) 6. (Vleugel et al. 2015) |
| ***TRIO***  **Gene ID: 7204** | 1. (B. Wang et al. 2015) 2. (Bellanger et al. 2000) 3. (Cannet, Schmidt, Delaval & Debant 2014) 4. (Debant et al. 1996) 5. (Katrancha et al. 2017) 6. (Pengelly et al. 2016) 7. (Seipel et al. 1999) 8. (Varvagiannis, Vissers, Baralle & de Vries 1993) 9. (Zheng et al. 2004) |
| ***STK38***  **Gene ID: 11329** | 1. (Bettoun et al. 2016) 2. (Bhattacharya, Large, Heizmann, Hemmings & Chazin 2003) 3. (Bisikirska et al. 2013) 4. (Cornils, Kohler, Hergovich & Hemmings 2011) 5. (Fukasawa, Enomoto & Miyagawa 2015) 6. (Hergovich, Lamla, Nigg, & Hemmings 2007) 7. (Yan et al. 2015) |
| ***PNCK***  **Gene ID: 13972** | 1. (Deb et al. 2008) 2. (Deb et al. 2011) 3. (Gardner, Ha, Reynolds & Chodosh 2000) 4. (S. Wu et al. 2013) |
| ***TAOK1***  **Gene ID: 57551** | 1. (Chen et al. 2003) 2. (Draviam et al. 2007) 3. (Raman, Earnest, Zhang, Zhao & Cobb 2007) 4. (Shrestha et al. 2014) 5. (Westhorpe, Diez, Gurden, Tighe & Taylor 2010) 6. (Wu & Wang 2008) |
| ***IRAK1***  **Gene ID: 3654** | 1. (Huang, Li, Sane & Li 2004) 2. (Jensen & Whitehead 2001) 3. (Mamidipudi, Lin, Seibenhener & Wooten 2004) 4. (Ordureau et al. 2008) 5. (Strelow, Kollewe & Wesche 2003) 6. (Vollmer et al. 2017) 7. (Wee et al. 2015) |
| ***PINK1***  **Gene ID: 65018** | 1. (Chen & Dorn 2013) 2. (Geisler et al. 2010) 3. (Kane & Youle 2011) 4. (Kane et al. 2014) 5. (Matsuda et al. 2010) 6. (O’Flanagan, Morais, Wurst, De Strooper & O’Neill 2015) 7. (Puschmann et al. 2017) 8. (Vives-Bauza et al. 2010) 9. (Wang et al. 2011) 10. (Xiong et al. 2009) 11. (Zhang et al. 2017) |
| ***PRKCE***  **Gene ID: 5581** | 1. (Brownlow, Pike, Zicha, Collinson & Parker 2014) 2. (Koliou, Fedonidis, Kalpachidou & Mangoura 2016) 3. (Saurin, Brownlow & Parker 2009) 4. (Sharma, Kakazu & Bazan 2007) |

**References**

1. Asghar, A., Lajeunesse, A., Dulla, K., Combes, G., Thebault, P., Nigg, E. A., & Elowe, S. (2015). Bub1 autophosphorylation feeds back to regulate kinetochore docking and promote localized substrate phosphorylation. *Nature Communications*, *6*, 8364.
2. Breit, C., Bange, T., Petrovic, A., Weir, J. R., Muller, F., Vogt, D., & Musacchio, A. (2015). Role of Intrinsic and Extrinsic Factors in the Regulation of the Mitotic Checkpoint Kinase Bub1. *PloS One*, *10*(12), e0144673.
3. Cahill, D. P., Lengauer, C., Yu, J., Riggins, G. J., Willson, J. K., Markowitz, S. D., et al. Vogelstein, B. (1998). Mutations of mitotic checkpoint genes in human cancers. *Nature*, *392*(6673), 300–303.
4. Gao, F., Ponte, J. F., Levy, M., Papageorgis, P., Cook, N. M., Ozturk, S., et al. Thiagalingam, S. (2009). hBub1 negatively regulates p53 mediated early cell death upon mitotic checkpoint activation. *Cancer Biology & Therapy*, *8*(7), 548–556.
5. Jessulat, M., Malty, R. H., Nguyen-Tran, D.-H., Deineko, V., Aoki, H., Vlasblom, J., et al. Babu, M. (2015). Spindle Checkpoint Factors Bub1 and Bub2 Promote DNA Double-Strand Break Repair by Nonhomologous End Joining. *Molecular and Cellular Biology*, *35*(14), 2448–2463.
6. Jia, L., Li, B., & Yu, H. (2016). The Bub1-Plk1 kinase complex promotes spindle checkpoint signalling through Cdc20 phosphorylation. *Nature Communications*, *7*, 10818.
7. Musio, A., Montagna, C., Zambroni, D., Indino, E., Barbieri, O., Citti, L., et al. Vezzoni, P. (2003). Inhibition of BUB1 results in genomic instability and anchorage-independent growth of normal human fibroblasts. *Cancer Research*, *63*(11), 2855–2863.
8. Nilsson, J. (2015). Bub1/BubR1: swiss army knives at kinetochores. *Cell Cycle* 14(19):2999-300.
9. Overlack, K., Primorac, I., Vleugel, M., Krenn, V., Maffini, S., Hoffmann, I., et al. Musacchio, A. (2015). A molecular basis for the differential roles of Bub1 and BubR1 in the spindle assembly checkpoint. *ELife*, *4*, e05269.
10. Ricke, R. M., Jeganathan, K. B., & van Deursen, J. M. (2011). Bub1 overexpression induces aneuploidy and tumor formation through Aurora B kinase hyperactivation. *The Journal of Cell Biology*, *193*(6), 1049–1064.
11. Wills, E. S., Cnossen, W. R., Veltman, J. A., Woestenenk, R., Steehouwer, M., Salomon, J., et al. Drenth, J. P. H. (2016). Chromosomal abnormalities in hepatic cysts point to novel polycystic liver disease genes. *European Journal of Human Genetics*, *24*(12), 1707–1714.
12. Chou, C.-K., Wu, C.-Y., Chen, J. Y.-F., Ng, M.-C., Wang, H.-M. D., Chen, J.-H., et al. Chiu, C.-C. (2015). BubR1 Acts as a Promoter in Cellular Motility of Human Oral Squamous Cancer Cells through Regulating MMP-2 and MMP-9. *International Journal of Molecular Sciences*, *16*(7), 15104–15117.
13. Hahn, M.-M., Vreede, L., Bemelmans, S. A. S. A., van der Looij, E., van Kessel, A. G., Schackert, H. K., et al. de Voer, R. M. (2016). Prevalence of germline mutations in the spindle assembly checkpoint gene BUB1B in individuals with early-onset colorectal cancer. *Genes, Chromosomes & Cancer*, *55*(11), 855–863.
14. Mansouri, N., Movafagh, A., Sayad, A., Heidary Pour, A., Taheri, M., Soleimani, S., et al. Mortazavi-Tabatabaei, S. A. (2016). Targeting of BUB1b Gene Expression in Sentinel Lymph Node Biopsies of Invasive Breast Cancer in Iranian Female Patients. *Asian Pacific Journal of Cancer Prevention*, *17*(S3), 317–321.
15. Weaver, B. A. A., Bonday, Z. Q., Putkey, F. R., Kops, G. J. P. L., Silk, A. D., & Cleveland, D. W. (2003). Centromere-associated protein-E is essential for the mammalian mitotic checkpoint to prevent aneuploidy due to single chromosome loss. *The Journal of Cell Biology*, *162*(4), 551–563.
16. Weaver, R. L., Limzerwala, J. F., Naylor, R. M., Jeganathan, K. B., Baker, D. J., & van Deursen, J. M. (2016). BubR1 alterations that reinforce mitotic surveillance act against aneuploidy and cancer. *ELife*, ,*5,* pii: e1662
17. Vleugel, M., Hoek, T. A., Tromer, E., Sliedrecht, T., Groenewold, V., Omerzu, M., & Kops, G. J. P. L. (2015). Dissecting the roles of human BUB1 in the spindle assembly checkpoint. *Journal of Cell Science*, *128*(16), 2975–2982.
18. Wang, B., Fang, J., Qu, L., Cao, Z., Zhou, J., & Deng, B. (2015). Upregulated TRIO expression correlates with a malignant phenotype in human hepatocellular carcinoma. *Tumour Biology : The Journal of the International Society for Oncodevelopmental Biology and Medicine*, *36*(9), 6901–6908.
19. Bellanger, J. M., Astier, C., Sardet, C., Ohta, Y., Stossel, T. P., & Debant, A. (2000). The Rac1- and RhoG-specific GEF domain of Trio targets filamin to remodel cytoskeletal actin. *Nature Cell Biology*, *2*(12), 888–892.
20. Cannet, A., Schmidt, S., Delaval, B., & Debant, A. (2014). Identification of a mitotic Rac-GEF, Trio, that counteracts MgcRacGAP function during cytokinesis. *Molecular Biology of the Cell*, *25*(25), 4063–4071.
21. Debant, A., Serra-Pages, C., Seipel, K., O’Brien, S., Tang, M., Park, S. H., & Streuli, M. (1996). The multidomain protein Trio binds the LAR transmembrane tyrosine phosphatase, contains a protein kinase domain, and has separate rac-specific and rho-specific guanine nucleotide exchange factor domains. *Proceedings of the National Academy of Sciences of the United States of America*, *93*(11), 5466–5471.
22. Katrancha, S. M., Wu, Y., Zhu, M., Eipper, B. A., Koleske, A. J., & Mains, R. E. (2017). Neurodevelopmental disease-associated de novo mutations and rare sequence variants affect TRIO GDP/GTP exchange factor activity. *Human Molecular Genetics*, *26*(23), 4728–4740.
23. Pengelly, R. J., Greville-Heygate, S., Schmidt, S., Seaby, E. G., Jabalameli, M. R., Mehta, S. G., et al. Baralle, D. (2016). Mutations specific to the Rac-GEF domain of TRIO cause intellectual disability and microcephaly. *Journal of Medical Genetics*, *53*(11), 735–742.
24. Seipel, K., Medley, Q. G., Kedersha, N. L., Zhang, X. A., O’Brien, S. P., Serra-Pages, C., et al. Streuli, M. (1999). Trio amino-terminal guanine nucleotide exchange factor domain expression promotes actin cytoskeleton reorganization, cell migration and anchorage-independent cell growth. *Journal of Cell Science*, *112 ( Pt 1*, 1825–1834.
25. Varvagiannis, K., Vissers, L. E. L. M., Baralle, D., & de Vries, B. B. A. (2017). TRIO-Related Intellectual Disability. In M. P. Adam, H. H. Ardinger, R. A. Pagon, S. E. Wallace, L. J. H. Bean, K. Stephens, & A. Amemiya (Eds.) editors GeneReviews® [internet]. Seattle (WA): University of Washington, Seattle; 1993-2018.
26. Zheng, M., Simon, R., Mirlacher, M., Maurer, R., Gasser, T., Forster, T., et al. Schraml, P. (2004). TRIO amplification and abundant mRNA expression is associated with invasive tumor growth and rapid tumor cell proliferation in urinary bladder cancer. *The American Journal of Pathology*, *165*(1), 63–69.
27. Bettoun, A., Joffre, C., Zago, G., Surdez, D., Vallerand, D., Gundogdu, R., et al. Hergovich, A. (2016). Mitochondrial clearance by the STK38 kinase supports oncogenic Ras-induced cell transformation. *Oncotarget*, *7*(28), 44142–44160.
28. Bhattacharya, S., Large, E., Heizmann, C. W., Hemmings, B., & Chazin, W. J. (2003). Structure of the Ca2+/S100B/NDR kinase peptide complex: insights into S100 target specificity and activation of the kinase. *Biochemistry*, *42*(49), 14416–14426.
29. Bisikirska, B. C., Adam, S. J., Alvarez, M. J., Rajbhandari, P., Cox, R., Lefebvre, C., et al. Califano, A. (2013). STK38 is a critical upstream regulator of MYC’s oncogenic activity in human B-cell lymphoma. *Oncogene*, *32*(45), 5283–5291.
30. Cornils, H., Kohler, R. S., Hergovich, A., & Hemmings, B. A. (2011). Human NDR kinases control G(1)/S cell cycle transition by directly regulating p21 stability. *Molecular and Cellular Biology*, *31*(7), 1382–1395.
31. Fukasawa, T., Enomoto, A., & Miyagawa, K. (2015). Serine-Threonine Kinase 38 regulates CDC25A stability and the DNA damage-induced G2/M checkpoint. *Cellular Signalling*, *27*(8), 1569–1575.
32. Hergovich, A., Lamla, S., Nigg, E. A., & Hemmings, B. A. (2007). Centrosome-associated NDR kinase regulates centrosome duplication. *Molecular Cell*, *25*(4), 625–634.
33. Yan, M., Chu, L., Qin, B., Wang, Z., Liu, X., Jin, C., et al. Yao, X. (2015). Regulation of NDR1 activity by PLK1 ensures proper spindle orientation in mitosis. *Scientific Reports*, *5*, 10449.
34. Deb, T. B., Coticchia, C. M., Barndt, R., Zuo, H., Dickson, R. B., & Johnson, M. D. (2008). Pregnancy-upregulated nonubiquitous calmodulin kinase induces ligand-independent EGFR degradation. *American Journal of Physiology Cell Physiology*, *295*(2), C365-77.
35. Deb, T. B., Zuo, A. H., Wang, Y., Barndt, R. J., Cheema, A. K., Sengupta, S., et al. Johnson, M. D. (2011). Pnck induces ligand-independent EGFR degradation by probable perturbation of the Hsp90 chaperone complex. *American Journal of Physiology Cell Physiology*, *300*(5), C1139-54.
36. Gardner, H. P., Ha, S. I., Reynolds, C., & Chodosh, L. A. (2000). The caM kinase, Pnck, is spatially and temporally regulated during murine mammary gland development and may identify an epithelial cell subtype involved in breast cancer. *Cancer Research*, *60*(19), 5571–5577.
37. Wu, S., Lv, Z., Wang, Y., Sun, L., Jiang, Z., Xu, C., et al. Wang, R. (2013). Increased expression of pregnancy up-regulated non-ubiquitous calmodulin kinase is associated with poor prognosis in clear cell renal cell carcinoma. *PloS One*, *8*(4), e59936.
38. Chen, Z., Raman, M., Chen, L., Lee, S. F., Gilman, A. G., & Cobb, M. H. (2003). TAO (thousand-and-one amino acid) protein kinases mediate signaling from carbachol to p38 mitogen-activated protein kinase and ternary complex factors. *The Journal of Biological Chemistry*, *278*(25), 22278–22283.
39. Draviam, V. M., Stegmeier, F., Nalepa, G., Sowa, M. E., Chen, J., Liang, A., et al. Elledge, S. J. (2007). A functional genomic screen identifies a role for TAO1 kinase in spindle-checkpoint signalling. *Nature Cell Biology*, *9*(5), 556–564.
40. Raman, M., Earnest, S., Zhang, K., Zhao, Y., & Cobb, M. H. (2007). TAO kinases mediate activation of p38 in response to DNA damage. *The EMBO Journal*, *26*(8), 2005–2014.
41. Shrestha, R. L., Tamura, N., Fries, A., Levin, N., Clark, J., & Draviam, V. M. (2014). TAO1 kinase maintains chromosomal stability by facilitating proper congression of chromosomes. *Open Biology*, *4*(6), 130108.
42. Westhorpe, F. G., Diez, M. A., Gurden, M. D. J., Tighe, A., & Taylor, S. S. (2010). Re-evaluating the role of Tao1 in the spindle checkpoint. *Chromosoma*, *119*(4), 371–379.
43. Wu, M.-F., & Wang, S.-G. (2008). Human TAO kinase 1 induces apoptosis in SH-SY5Y cells. *Cell Biology International*, *32*(1), 151–156.
44. Huang, Y., Li, T., Sane, D. C., & Li, L. (2004). IRAK1 serves as a novel regulator essential for lipopolysaccharide-induced interleukin-10 gene expression. *The Journal of Biological Chemistry*, *279*(49), 51697–51703.
45. Jensen, L. E., & Whitehead, A. S. (2001). IRAK1b, a novel alternative splice variant of interleukin-1 receptor-associated kinase (IRAK), mediates interleukin-1 signaling and has prolonged stability. *The Journal of Biological Chemistry*, *276*(31), 29037–29044.
46. Mamidipudi, V., Lin, C., Seibenhener, M. L., & Wooten, M. W. (2004). Regulation of interleukin receptor-associated kinase (IRAK) phosphorylation and signaling by iota protein kinase C. *The Journal of Biological Chemistry*, *279*(6), 4161–4165.
47. Ordureau, A., Smith, H., Windheim, M., Peggie, M., Carrick, E., Morrice, N., & Cohen, P. (2008). The IRAK-catalysed activation of the E3 ligase function of Pellino isoforms induces the Lys63-linked polyubiquitination of IRAK1. *The Biochemical Journal*, *409*(1), 43–52.
48. Strelow, A., Kollewe, C., & Wesche, H. (2003). Characterization of Pellino2, a substrate of IRAK1 and IRAK4. *FEBS Letters*, *547*(1–3), 157–161.
49. Vollmer, S., Strickson, S., Zhang, T., Gray, N., Lee, K. L., Rao, V. R., & Cohen, P. (2017). The mechanism of activation of IRAK1 and IRAK4 by interleukin-1 and Toll-like receptor agonists. *The Biochemical Journal*, *474*(12), 2027–2038.
50. Wee, Z. N., Yatim, S. M. J. M., Kohlbauer, V. K., Feng, M., Goh, J. Y., Bao, Y., et al. Yu, Q. (2015). IRAK1 is a therapeutic target that drives breast cancer metastasis and resistance to paclitaxel. *Nature Communications*, *6*, 8746.
51. Chen, Y., & Dorn, G. W. 2nd. (2013). PINK1-phosphorylated mitofusin 2 is a Parkin receptor for culling damaged mitochondria. *Science (New York, N.Y.)*, *340*(6131), 471–475.
52. Geisler, S., Holmstrom, K. M., Treis, A., Skujat, D., Weber, S. S., Fiesel, F. C., et al. Springer, W. (2010). The PINK1/Parkin-mediated mitophagy is compromised by PD-associated mutations. *Autophagy*, *6*(7), 871–878.
53. Kane, L. A., Lazarou, M., Fogel, A. I., Li, Y., Yamano, K., Sarraf, S. A., et al. Youle, R. J. (2014). PINK1 phosphorylates ubiquitin to activate Parkin E3 ubiquitin ligase activity. *The Journal of Cell Biology*, *205*(2), 143–153.
54. Kane, L. A., & Youle, R. J. (2011). PINK1 and Parkin flag Miro to direct mitochondrial traffic. *Cell*, *147*(4), 721–723.
55. Matsuda, N., Sato, S., Shiba, K., Okatsu, K., Saisho, K., Gautier, C. A., et al. Tanaka, K. (2010). PINK1 stabilized by mitochondrial depolarization recruits Parkin to damaged mitochondria and activates latent Parkin for mitophagy. *The Journal of Cell Biology*, *189*(2), 211–221.
56. O’Flanagan, C. H., Morais, V. A., Wurst, W., De Strooper, B., & O’Neill, C. (2015). The Parkinson’s gene PINK1 regulates cell cycle progression and promotes cancer-associated phenotypes. *Oncogene*, *34*(11), 1363–1374.
57. Puschmann, A., Fiesel, F. C., Caulfield, T. R., Hudec, R., Ando, M., Truban, D., et al. Springer, W. (2017). Heterozygous PINK1 p.G411S increases risk of Parkinson’s disease via a dominant-negative mechanism. *Brain : A Journal of Neurology*, *140*(1), 98–117.
58. Vives-Bauza, C., Zhou, C., Huang, Y., Cui, M., de Vries, R. L. A., Kim, J., et al. Przedborski, S. (2010). PINK1-dependent recruitment of Parkin to mitochondria in mitophagy. *Proceedings of the National Academy of Sciences of the United States of America*, *107*(1), 378–383.
59. Wang, X., Winter, D., Ashrafi, G., Schlehe, J., Wong, Y. L., Selkoe, D., et al. Schwarz, T. L. (2011). PINK1 and Parkin target Miro for phosphorylation and degradation to arrest mitochondrial motility. *Cell*, *147*(4), 893–906.
60. Xiong, H., Wang, D., Chen, L., Choo, Y. S., Ma, H., Tang, C., et al. Zhang, Z. (2009). Parkin, PINK1, and DJ-1 form a ubiquitin E3 ligase complex promoting unfolded protein degradation. *The Journal of Clinical Investigation*, *119*(3), 650–660.
61. Zhang, R., Gu, J., Chen, J., Ni, J., Hung, J., Wang, Z., et al. Ji, L. (2017). High expression of PINK1 promotes proliferation and chemoresistance of NSCLC. *Oncology Reports*, *37*(4), 2137–2146.
62. Brownlow, N., Pike, T., Zicha, D., Collinson, L., & Parker, P. J. (2014). Mitotic catenation is monitored and resolved by a PKCepsilon-regulated pathway. *Nature Communications*, *5*, 5685.
63. Koliou, X., Fedonidis, C., Kalpachidou, T., & Mangoura, D. (2016). Nuclear import mechanism of neurofibromin for localization on the spindle and function in chromosome congression. *Journal of Neurochemistry*, *136*(1), 78–91.
64. Saurin, A. T., Brownlow, N., & Parker, P. J. (2009). Protein kinase C epsilon in cell division: control of abscission. *Cell Cycle*, *8*(4), 549–555.
65. Sharma, G. D., Kakazu, A., & Bazan, H. E. P. (2007). Protein kinase C alpha and epsilon differentially modulate hepatocyte growth factor-induced epithelial proliferation and migration. *Experimental Eye Research*, *85*(2), 289–297.

**Table S3 Formation of micronuclei (MNi) and nucleoplasmic bridges (NPBs) after knockdown of a gene of interest in RPE cells**

| **Gene** | **Number of normal cells** | **Number of MNi** | **Number of NPBs** | **Total number of cells** | **% of normal cells** | **% of MNi** | **% of NPBs** |
| --- | --- | --- | --- | --- | --- | --- | --- |
| *STK38* | 149 | 3 | 256 | 408 | 36.5 | 0.7 | 62.7*** |
| *IRAK1* | 120 | 1 | 45 | 166 | 72.3 | 0.6 | 27.1 |
| *PINK1* | 34 | 2 | 203 | 239 | 14.2 | 0.8 | 84.9*** |
| *PNCK* | 129 | 6 | 42 | 177 | 72.9 | 3.4** | 23.7 |
| *TRIO* | 263 | 19 | 24 | 306 | 85.9 | 6.2** | 7.8 |
| *PRKCE* | 231 | 9 | 170 | 410 | 56.3 | 2.2 | 41.5*** |
| *TAOK1* | 214 | 3 | 80 | 297 | 72.1 | 1.0 | 26.9 |
| *BUB1* | 312 | 12 | 80 | 404 | 77.2 | 3.0** | 19.8 |
| *BUB1B* | 316 | 31 | 63 | 410 | 77.1 | 7.6** | 15.4 |
| scr. siRNA* | 386 | 1 | 18 | 405 | 95.3 | 0.2 | 4.4 |

*scr. siRNA is a negative control.

** Knockdown of *PNCK, TRIO, BUB1* and *BUB1B* genes produces the highest percentage of MNi.

***Knockdown of *STK38,* *PINK1* and *PRKCE* genes produces the highest percentage of NPBs.

**Table S4 Formation of micronuclei (MNi) and nucleoplasmic bridges (NPBs) after knockdown of a gene of interest in HT1080 cells**

| **Gene** | **Number of normal cells** | **Number of MNi** | **Number of NPBs** | **Total number of cells** | **% of normal cells** | **% of MNi** | **% of NPBs** |
| --- | --- | --- | --- | --- | --- | --- | --- |
| *STK38* | 257 | 91 | 42 | 390 | 65.9 | 23.3 | 10.8 |
| *IRAK1* | 197 | 51 | 67 | 315 | 62.5 | 16.2 | 21.3 |
| *PINK1* | 241 | 91 | 19 | 351 | 68.7 | 25.9 | 5.4 |
| *PNCK* | 284 | 100 | 36 | 420 | 67.6 | 23.8 | 8.6 |
| *TRIO* | 308 | 100 | 14 | 422 | 73.0 | 23.7 | 3.3 |
| *PRKCE* | 290 | 83 | 39 | 412 | 70.4 | 20.1 | 9.5 |
| *TAOK1* | 297 | 110 | 48 | 455 | 65.3 | 24.2 | 10.5 |
| *BUB1* | 215 | 121 | 64 | 400 | 53.8 | 30.3 | 16.0 |
| *BUB1B* | 209 | 113 | 37 | 359 | 58.2 | 31.5 | 10.3 |
| scr. siRNA* | 370 | 32 | 12 | 414 | 89.4 | 7.7 | 2.9 |

*scr. siRNA is a negative control.

# Table S5 Bioinformatical analysis of NCI-60 database

| **Correlations with gene transcript expression** | | | | | | | |
| --- | --- | --- | --- | --- | --- | --- | --- |
| **Genes associated with chromosomal transfer** | **Cytogenetics; Modal chromosomal number** | **Cytogenetics; # of structurally rearanged**  **chromosomes** | **Cytogenetics; Numerical complexity** | **Cytogeneti; Numericalet rogeneity** | **Cytogenetics; Fraction of normal chromosomes that experience numerical heterogeneity** | **Cytogenetics; Fraction of abnormal chromosomes that experience numerical heterogeneity** | **Cytogenetics; Structural heterogeneity** |
| *ATM* | -0.048 | 0.020 | -0.049 | 0.135 | 0.081 | 0.112 | 0.186 |
| *BLK* | 0.155 | 0.060 | 0.099 | -0.093 | -0.107 | -0.140 | -0.046 |
| *BTK* | -0.084 | -0.081 | -0.092 | -0.206 | -0.213 | -0.207 | -0.017 |
| *BUB1* | -0.124 | 0.047 | -0.017 | -0.225 | -0.265 | -0.271 | -0.036 |
| *BUB1B* | -0.071 | -0.024 | -0.239 | -0.195 | -0.136 | 0.025 | -0.105 |
| *CAMK2G* | -0.022 | 0.285 | -0.053 | -0.159 | -0.187 | -0.187 | -0.071 |
| *CRIM1* | -0.001 | 0.207 | 0.257 | **0.358** | 0.292 | 0.061 | 0.069 |
| *CSK* | -0.050 | -0.022 | -0.133 | -0.301 | -0.259 | -0.104 | 0.022 |
| *CSNK1G2* | -0.127 | **-0.375** | **-0.409** | -0.219 | -0.277 | -0.186 | -0.069 |
| *FRK* | -0.064 | 0.090 | -0.017 | -0.007 | 0.030 | 0.184 | -0.064 |
| *HIPK2* | 0.290 | -0.147 | 0.310 | **0.418** | **0.459** | 0.170 | 0.186 |
| *IRAK1* | 0.092 | 0.211 | 0.213 | 0.299 | 0.300 | 0.187 | 0.109 |
| *ITPKB* | 0.170 | -0.181 | 0.010 | -0.181 | -0.059 | -0.027 | 0.072 |
| *KSR2* | -0.227 | 0.106 | -0.323 | -0.306 | -0.240 | -0.091 | -0.151 |
| *MAPK7* | 0.088 | 0.142 | 0.266 | **0.448** | **0.391** | **0.379** | 0.185 |
| *MYLK* | **0.354** | 0.004 | **0.412** | **0.472** | **0.415** | 0.075 | 0.255 |
| *MYLK4* | 0.000 | 0.240 | 0.070 | 0.010 | -0.030 | 0.110 | 0.120 |
| *NEK9* | -0.037 | -0.279 | -0.026 | 0.090 | 0.170 | 0.034 | 0.025 |
| *PDXK* | 0.033 | 0.018 | 0.045 | 0.089 | 0.099 | -0.029 | -0.173 |
| *PHKB* | -0.126 | -0.035 | -0.123 | -0.056 | -0.090 | -0.164 | -0.088 |
| *PHKG1* | -0.167 | -0.125 | -0.311 | -0.032 | -0.088 | 0.084 | -0.119 |
| *PINK1* | 0.312 | 0.270 | 0.320 | **0.372** | 0.253 | 0.137 | 0.302 |
| *PNCK* | 0.045 | 0.237 | 0.126 | 0.072 | 0.043 | 0.079 | 0.058 |
| *PRKCE* | 0.205 | 0.128 | 0.252 | -0.043 | -0.009 | 0.062 | 0.115 |
| *RBKS* | -0.061 | -0.077 | -0.074 | 0.006 | 0.022 | 0.049 | -0.256 |
| *STK11* | -0.068 | -0.265 | -0.198 | -0.013 | -0.137 | -0.122 | -0.109 |
| *STK38* | -0.025 | 0.146 | 0.094 | 0.205 | 0.142 | 0.121 | 0.007 |
| *TAOK1* | 0.210 | -0.139 | 0.111 | 0.298 | **0.339** | 0.120 | 0.133 |
| *TNK2* | 0.017 | 0.108 | 0.100 | -0.153 | -0.141 | -0.099 | 0.023 |
| *TPD52L3* | 0.085 | 0.301 | 0.205 | 0.136 | 0.090 | 0.033 | -0.008 |
| *TRIO* | 0.195 | 0.311 | 0.486 | 0.433 | 0.378 | 0.186 | 0.291 |
| *TTBK1* | -0.018 | 0.031 | -0.017 | -0.099 | -0.107 | -0.183 | -0.163 |
| **Correlations with amino acid changing variants** | | | | | | | |
| *ATM* | -0.160 | -0.110 | -0.266 | -0.187 | -0.214 | -0.010 | -0.187 |
| *BLK* | 0.124 | 0.242 | 0.116 | 0.009 | -0.021 | -0.043 | 0.150 |
| *BTK* | -0.014 | 0.151 | 0.121 | 0.143 | 0.026 | 0.051 | -0.052 |
| *BUB1* | -0.094 | -0.268 | **-0.339** | -0.133 | -0.174 | 0.021 | -0.130 |
| *BUB1B* | 0.227 | -0.084 | 0.177 | 0.200 | 0.196 | 0.051 | 0.257 |
| *CAMK2G* | -0.141 | -0.185 | -0.234 | -0.199 | -0.245 | -0.222 | -0.119 |
| *CRIM1* | -0.143 | 0.100 | -0.159 | **-0.336** | -0.315 | -0.165 | -0.026 |
| *CSK* | -0.173 | -0.159 | -0.255 | -0.156 | -0.177 | -0.222 | -0.102 |
| *CSNK1G2* | -0.242 | -0.165 | -0.193 | -0.203 | -0.142 | -0.139 | -0.152 |
| *FRK* | -0.161 | 0.009 | -0.144 | -0.191 | -0.231 | 0.039 | -0.074 |
| *IRAK1* | 0.104 | -0.073 | 0.172 | 0.193 | 0.156 | 0.166 | 0.303 |
| *ITPKB* | -0.233 | -0.120 | -0.121 | 0.051 | -0.029 | 0.188 | 0.048 |
| *MAPK7* | -0.075 | -0.046 | -0.133 | -0.039 | -0.051 | -0.032 | -0.022 |
| *MYLK* | -0.168 | -0.072 | -0.101 | -0.127 | -0.072 | 0.016 | -0.097 |
| *MYLK4* | -0.010 | -0.040 | -0.170 | -0.100 | -0.060 | 0.040 | -0.060 |
| *NEK9* | 0.105 | -0.022 | 0.033 | 0.088 | 0.076 | 0.012 | -0.117 |
| *PDXK* | -0.014 | 0.073 | -0.005 | -0.135 | -0.130 | -0.096 | -0.027 |
| *PHKB* | -0.077 | -0.111 | -0.153 | -0.196 | -0.193 | 0.015 | -0.125 |
| *PINK1* | -0.017 | 0.185 | 0.055 | -0.040 | 0.040 | -0.027 | 0.040 |
| *PNCK* | 0.176 | 0.159 | 0.222 | -0.050 | -0.021 | 0.188 | -0.014 |
| *PRKCE* | -0.094 | -0.213 | -0.207 | -0.156 | -0.152 | 0.019 | -0.110 |
| *STK38* | -0.001 | 0.019 | -0.020 | -0.157 | -0.145 | -0.201 | -0.115 |
| *TNK2* | -0.077 | -0.127 | -0.095 | -0.079 | -0.001 | 0.049 | -0.209 |
| *TPD52L3* | 0.314 | -0.037 | 0.281 | -0.071 | -0.089 | -0.222 | **0.429** |
| *TRIO* | -0.280 | -0.190 | -0.170 | -0.121 | -0.171 | 0.033 | -0.129 |
| *TTBK1* | -0.129 | 0.101 | -0.078 | -0.192 | -0.286 | -0.233 | -0.215 |
| **Correlations with protein function affecting variants** | | | | | | | |
| *ATM* | -0.077 | -0.148 | -0.144 | -0.093 | -0.159 | 0.142 | -0.084 |
| *BLK* | 0.124 | 0.242 | 0.116 | 0.009 | -0.021 | -0.043 | 0.150 |
| *BUB1* | -0.064 | -0.186 | -0.254 | -0.160 | -0.148 | -0.111 | -0.079 |
| *BUB1B* | 0.225 | -0.159 | -0.109 | -0.071 | -0.089 | -0.222 | -0.111 |
| *CAMK2G* | -0.141 | -0.185 | -0.234 | -0.199 | -0.245 | -0.222 | -0.119 |
| *CRIM1* | -0.014 | 0.073 | -0.005 | -0.135 | -0.130 | -0.096 | -0.027 |
| *CSK* | -0.173 | -0.159 | -0.255 | -0.156 | -0.177 | -0.222 | -0.102 |
| *CSNK1G2* | -0.165 | -0.146 | -0.234 | -0.221 | -0.245 | -0.222 | -0.119 |
| *FRK* | -0.177 | -0.033 | -0.162 | -0.140 | -0.188 | 0.101 | -0.069 |
| *IRAK1* | 0.034 | 0.241 | 0.016 | -0.071 | -0.089 | -0.036 | 0.007 |
| *ITPKB* | **-0.341** | -0.069 | -0.301 | -0.143 | -0.272 | 0.108 | -0.127 |
|  |  |  |  |  |  |  |  |
| *MAPK7* | -0.074 | -0.058 | -0.145 | -0.047 | -0.059 | -0.054 | -0.033 |
| *MYLK* | -0.184 | 0.001 | -0.156 | 0.028 | -0.086 | 0.245 | -0.044 |
| *MYLK4* | -0.170 | -0.160 | -0.260 | -0.160 | -0.180 | -0.220 | -0.100 |
| *NEK9* | -0.178 | -0.043 | -0.146 | -0.029 | -0.003 | -0.022 | -0.112 |
| *PHKB* | -0.116 | -0.200 | -0.272 | -0.074 | -0.084 | 0.156 | -0.091 |
| *PNCK* | 0.176 | 0.159 | 0.222 | -0.050 | -0.021 | 0.188 | -0.014 |
| *PRKCE* | -0.082 | -0.110 | -0.038 | -0.058 | -0.069 | 0.123 | -0.079 |
| *STK38* | 0.225 | -0.159 | -0.109 | -0.071 | -0.089 | -0.222 | -0.111 |
| *IRAK1* | 0.034 | 0.241 | 0.016 | -0.071 | -0.089 | -0.036 | 0.007 |
| *ITPKB* | **-0.341** | -0.069 | -0.301 | -0.143 | -0.272 | 0.108 | -0.127 |
|  |  |  |  |  |  |  |  |
| *MAPK7* | -0.074 | -0.058 | -0.145 | -0.047 | -0.059 | -0.054 | -0.033 |
| *MYLK* | -0.184 | 0.001 | -0.156 | 0.028 | -0.086 | 0.245 | -0.044 |
| *MYLK4* | -0.170 | -0.160 | -0.260 | -0.160 | -0.180 | -0.220 | -0.100 |
| *NEK9* | -0.178 | -0.043 | -0.146 | -0.029 | -0.003 | -0.022 | -0.112 |
| *PHKB* | -0.116 | -0.200 | -0.272 | -0.074 | -0.084 | 0.156 | -0.091 |
| *PNCK* | 0.176 | 0.159 | 0.222 | -0.050 | -0.021 | 0.188 | -0.014 |
| *PRKCE* | -0.082 | -0.110 | -0.038 | -0.058 | -0.069 | 0.123 | -0.079 |
| *STK38* | 0.225 | -0.159 | -0.109 | -0.071 | -0.089 | -0.222 | -0.111 |
| *TNK2* | -0.313 | -0.211 | **-0.359** | -0.236 | -0.304 | -0.165 | -0.167 |
| *TPD52L3* | 0.314 | -0.037 | 0.281 | -0.071 | -0.089 | -0.222 | **0.429** |
| *TRIO* | -0.249 | -0.094 | -0.109 | -0.129 | -0.162 | 0.031 | -0.079 |
| *TTBK1* | -0.080 | 0.022 | -0.052 | -0.069 | -0.112 | -0.064 | -0.044 |

Red, plain text indicates statistical significance of positive correlation, with p< 0.05.

Red, bold text indicates statistical significance of positive correlation, with p< 0.01.

Blue, bold text indicates statistical significance of negative correlation, with p< 0.05.

Blue, plain text indicates statistical significance of negative correlation, with p< 0.01.

**Table S6 Relationships between the newly discovered CIN genes and known genes**

| **From Molecule(s)** | **Relationship Type** | **To Molecule(s)** | **Relationship Type** | **Count** |
| --- | --- | --- | --- | --- |
| ATM | activation | BUB1 | protein-protein interactions | 54 |
| ATM | activation | CASP3 | activation | 27 |
| ATM | inhibition | MDM2 | phosphorylation | 21 |
| ATM | inhibition | STK11 | expression | 11 |
| ATM | localization | HIPK2 | regulation of binding | 8 |
| ATM | phosphorylation | BUB1 | inhibition | 6 |
| ATM | phosphorylation | HIPK2 | localization | 6 |
| ATM | phosphorylation | MDM2 | transcription | 6 |
| ATM | phosphorylation | STK11 | molecular cleavage | 5 |
| ATM | protein-protein interactions | MDM2 | ubiquitination | 2 |
| ATM | ubiquitination | MDM2 | membership | 1 |
| AURKB | activation | ATM | protein-DNA interactions | 1 |
| AURKB | activation | MAD2L1 | translocation | 1 |
| AURKB | localization | BUB1B |  |  |
| AURKB | phosphorylation | ATM |  |  |
| AURKB | phosphorylation | MAD2L1 |  |  |
| AURKB | protein-protein interactions | BUB1B |  |  |
| BLK | activation | BTK |  |  |
| BLK | phosphorylation | BTK |  |  |
| BTK | activation | ATM |  |  |
| BTK | phosphorylation | ATM |  |  |
| BUB1 | activation | AURKB |  |  |
| BUB1 | localization | AURKB |  |  |
| BUB1 | protein-protein interactions | AURKB |  |  |
| BUB1B | protein-protein interactions | AURKB |  |  |
| BUB1B | protein-protein interactions | BUB1 |  |  |
| BUB1B | protein-protein interactions | MAD2L1 |  |  |
| BUB1B | regulation of binding | MAD2L1 |  |  |
| CASP3 | activation | ATM |  |  |
| CASP3 | molecular cleavage | ATM |  |  |
| CASP3 | molecular cleavage | MDM2 |  |  |
| CASP3 | molecular cleavage | TAOK1 |  |  |
| CASP3 | protein-protein interactions | MDM2 |  |  |
| CSK | activation | BTK |  |  |
| CSK | protein-protein interactions | CAMK2G |  |  |
| ERK | activation | FOS |  |  |
| ERK | activation | MYC |  |  |
| ERK | expression | FOS |  |  |
| ERK | expression | MYC |  |  |
| ERK | membership | MAPK7 |  |  |
| ERK | phosphorylation | FOS |  |  |
| ERK | phosphorylation | MYC |  |  |
| ERK | regulation of binding | FOS |  |  |
| ERK | regulation of binding | MYC |  |  |
| ERK | transcription | FOS |  |  |
| ERK | transcription | MYC |  |  |
| ESR1 | activation | SRC |  |  |
| ESR1 | expression | HSP90AA1 |  |  |
| ESR1 | expression | MYC |  |  |
| ESR1 | expression | TAOK1 |  |  |
| ESR1 | expression | TRIO |  |  |
| ESR1 | inhibition | HSP90AA1 |  |  |
| ESR1 | phosphorylation | SRC |  |  |
| ESR1 | protein-DNA interactions | MYC |  |  |
| ESR1 | protein-protein interactions | HSP90AA1 |  |  |
| ESR1 | protein-protein interactions | MYC |  |  |
| ESR1 | protein-protein interactions | SRC |  |  |
| ESR1 | regulation of binding | MYC |  |  |
| ESR1 | transcription | MYC |  |  |
| FOS | expression | TAOK1 |  |  |
| FRK | protein-protein interactions | CSK |  |  |
| HIPK2 | activation | ATM |  |  |
| HIPK2 | phosphorylation | ATM |  |  |
| HSP90AA1 | inhibition | ESR1 |  |  |
| HSP90AA1 | protein-protein interactions | ESR1 |  |  |
| IRAK1 | protein-protein interactions | BTK |  |  |
| IRAK1 | protein-protein interactions | PRKCE |  |  |
| IRAK1 | translocation | PRKCE |  |  |
| KSR2 | activation | ERK |  |  |
| KSR2 | phosphorylation | ERK |  |  |
| KSR2 | protein-protein interactions | ERK |  |  |
| KSR2 | protein-protein interactions | HSP90AA1 |  |  |
| LRRK2 | protein-protein interactions | HSP90AA1 |  |  |
| MAD1L1 | localization | MAD2L1 |  |  |
| MAD1L1 | protein-protein interactions | BUB1B |  |  |
| MAD1L1 | protein-protein interactions | MAD2L1 |  |  |
| MAD1L1 | regulation of binding | MAD2L1 |  |  |
| MAD2L1 | activation | AURKB |  |  |
| MAD2L1 | localization | AURKB |  |  |
| MAD2L1 | protein-protein interactions | BUB1 |  |  |
| MAD2L1 | protein-protein interactions | BUB1B |  |  |
| MAD2L1 | protein-protein interactions | MAD1L1 |  |  |
| MAD2L1 | regulation of binding | BUB1B |  |  |
| MDM2 | inhibition | ATM |  |  |
| MDM2 | protein-protein interactions | ATM |  |  |
| MDM2 | protein-protein interactions | CASP3 |  |  |
| MOB1A | activation | STK38 |  |  |
| MOB1A | protein-protein interactions | AURKB |  |  |
| MOB1A | protein-protein interactions | STK38 |  |  |
| MYC | activation | ERK |  |  |
| MYC | activation | MDM2 |  |  |
| MYC | expression | MDM2 |  |  |
| MYC | expression | PNCK |  |  |
| MYC | molecular cleavage | MDM2 |  |  |
| MYC | protein-protein interactions | ESR1 |  |  |
| MYC | protein-protein interactions | MDM2 |  |  |
| MYC | transcription | MDM2 |  |  |
| MYLK | activation | SRC |  |  |
| MYLK | localization | SRC |  |  |
| MYLK | protein-protein interactions | SRC |  |  |
| MYLK4 | protein-protein interactions | CDC37 |  |  |
| MYLK4 | protein-protein interactions | HSP90AA1 |  |  |
| NEK9 | protein-protein interactions | MDM2 |  |  |
| PHKB | protein-protein interactions | AURKB |  |  |
| PINK1 | protein-protein interactions | CDC37 |  |  |
| PINK1 | protein-protein interactions | HSP90AA1 |  |  |
| PINK1 | protein-protein interactions | IRAK1 |  |  |
| PLK1 | protein-protein interactions | BUB1B |  |  |
| PLK1 | protein-protein interactions | NEK9 |  |  |
| PRKCE | protein-protein interactions | IRAK1 |  |  |
| PTPN11 | activation | ERK |  |  |
| PTPN11 | activation | SRC |  |  |
| PTPN11 | inhibition | SRC |  |  |
| PTPN11 | phosphorylation | ERK |  |  |
| PTPN11 | phosphorylation | SRC |  |  |
| PTPN11 | protein-protein interactions | PNCK |  |  |
| PTPN11 | protein-protein interactions | SRC |  |  |
| SRC | activation | ESR1 |  |  |
| SRC | activation | FOS |  |  |
| SRC | activation | MAPK7 |  |  |
| SRC | activation | MYLK |  |  |
| SRC | activation | PTPN11 |  |  |
| SRC | expression | FOS |  |  |
| SRC | expression | MYC |  |  |
| SRC | molecular cleavage | ESR1 |  |  |
| SRC | phosphorylation | ESR1 |  |  |
| SRC | phosphorylation | FOS |  |  |
| SRC | phosphorylation | MAPK7 |  |  |
| SRC | phosphorylation | MYLK |  |  |
| SRC | phosphorylation | PTPN11 |  |  |
| SRC | protein-protein interactions | ESR1 |  |  |
| SRC | protein-protein interactions | MYLK |  |  |
| SRC | protein-protein interactions | PTPN11 |  |  |
| SRC | regulation of binding | ESR1 |  |  |
| SRC | regulation of binding | MYC |  |  |
| SRC | transcription | FOS |  |  |
| SRC | transcription | MYC |  |  |
| SRC | ubiquitination | ESR1 |  |  |
| STK38 | protein-protein interactions | MDM2 |  |  |
| STK38 | protein-protein interactions | MOB1A |  |  |
| TAOK1 | activation | MAPT |  |  |
| TAOK1 | phosphorylation | MAPT |  |  |
| TAOK1 | protein-protein interactions | LRRK2 |  |  |
| TNK2 | protein-protein interactions | CSK |  |  |
| TNK2 | protein-protein interactions | HSP90AA1 |  |  |
| TRIO | protein-protein interactions | MYC |  |  |
| UBC | protein-protein interactions | MDM2 |  |  |
| UBC | protein-protein interactions | PINK1 |  |  |
| UBC | protein-protein interactions | PNCK |  |  |

**IRAK1-BTK**

1. Jefferies, C. A., Doyle, S., Brunner, C., Dunne, A., Brint, E., Wietek, C., … O’Neill, L. A. J. (2003). Bruton’s tyrosine kinase is a Toll/interleukin-1 receptor domain-binding protein  that participates in nuclear factor kappaB activation by Toll-like receptor 4. *The Journal of Biological Chemistry*, *278*(28), 26258–26264. https://doi.org/10.1074/jbc.M301484200

**IRAK1-PRKCE**

1. Gan, L., & Li, L. (2010). Interleukin-1 Receptor-Associated Kinase-1 (IRAK-1) functionally associates with  PKCepsilon and VASP in the regulation of macrophage migration. *Molecular Immunology*, *47*(6), 1278–1282. https://doi.org/10.1016/j.molimm.2009.12.004
2. Udgata, A., Qureshi, R., & Mukhopadhyay, S. (2016). Transduction of Functionally Contrasting Signals by Two Mycobacterial PPE Proteins Downstream of TLR2 Receptors. *Journal of Immunology (Baltimore, Md. : 1950)*, *197*(5), 1776–1787. https://doi.org/10.4049/jimmunol.1501816

**PINK1-CDC37**

1. Weihofen, A., Ostaszewski, B., Minami, Y., & Selkoe, D. J. (2008). Pink1 Parkinson mutations, the Cdc37/Hsp90 chaperones and Parkin all influence the maturation or subcellular distribution of Pink1. *Human Molecular Genetics*, *17*(4), 602–616. https://doi.org/10.1093/hmg/ddm334
2. Moriwaki, Y., Kim, Y.-J., Ido, Y., Misawa, H., Kawashima, K., Endo, S., & Takahashi, R. (2008). L347P PINK1 mutant that fails to bind to Hsp90/Cdc37 chaperones is rapidly degraded in a proteasome-dependent manner. *Neuroscience Research*, *61*(1), 43–48. https://doi.org/10.1016/j.neures.2008.01.006
3. Imai, Y., Kanao, T., Sawada, T., Kobayashi, Y., Moriwaki, Y., Ishida, Y., … Takahashi, R. (2010). The loss of PGAM5 suppresses the mitochondrial degeneration caused by inactivation of PINK1 in Drosophila. *PLoS Genetics*, *6*(12), e1001229. https://doi.org/10.1371/journal.pgen.1001229
4. Taipale, M., Tucker, G., Peng, J., Krykbaeva, I., Lin, Z.-Y., Larsen, B., … Lindquist, S. (2014). A quantitative chaperone interaction network reveals the architecture of cellular protein homeostasis pathways. *Cell*, *158*(2), 434–448. https://doi.org/10.1016/j.cell.2014.05.039
5. Ando, M., Fiesel, F. C., Hudec, R., Caulfield, T. R., Ogaki, K., Gorka-Skoczylas, P., … Springer, W. (2017). The PINK1 p.I368N mutation affects protein stability and ubiquitin kinase activity. *Molecular Neurodegeneration*, *12*(1), 32. https://doi.org/10.1186/s13024-017-0174-z

**PINK1-HSP90AA1**

1. Weihofen, A., Ostaszewski, B., Minami, Y., & Selkoe, D. J. (2008). Pink1 Parkinson mutations, the Cdc37/Hsp90 chaperones and Parkin all influence the maturation or subcellular distribution of Pink1. *Human Molecular Genetics*, *17*(4), 602–616. https://doi.org/10.1093/hmg/ddm334
2. Moriwaki, Y., Kim, Y.-J., Ido, Y., Misawa, H., Kawashima, K., Endo, S., & Takahashi, R. (2008). L347P PINK1 mutant that fails to bind to Hsp90/Cdc37 chaperones is rapidly degraded in a proteasome-dependent manner. *Neuroscience Research*, *61*(1), 43–48. https://doi.org/10.1016/j.neures.2008.01.006
3. Imai, Y., Kanao, T., Sawada, T., Kobayashi, Y., Moriwaki, Y., Ishida, Y., … Takahashi, R. (2010). The loss of PGAM5 suppresses the mitochondrial degeneration caused by inactivation of PINK1 in Drosophila. *PLoS Genetics*, *6*(12), e1001229. https://doi.org/10.1371/journal.pgen.1001229
4. Taipale, M., Krykbaeva, I., Koeva, M., Kayatekin, C., Westover, K. D., Karras, G. I., & Lindquist, S. (2012). Quantitative analysis of HSP90-client interactions reveals principles of substrate recognition. *Cell*, *150*(5), 987–1001. https://doi.org/10.1016/j.cell.2012.06.047
5. Ando, M., Fiesel, F. C., Hudec, R., Caulfield, T. R., Ogaki, K., Gorka-Skoczylas, P., … Springer, W. (2017). The PINK1 p.I368N mutation affects protein stability and ubiquitin kinase activity. *Molecular Neurodegeneration*, *12*(1), 32. https://doi.org/10.1186/s13024-017-0174-z

**PINK1-IRAK1**

1. Lee, H. J., & Chung, K. C. (2012). PINK1 positively regulates IL-1beta-mediated signaling through Tollip and IRAK1 modulation. *Journal of Neuroinflammation*, *9*, 271. https://doi.org/10.1186/1742-2094-9-271

**PINK1-UBS**

1. Kane, L. A., Lazarou, M., Fogel, A. I., Li, Y., Yamano, K., Sarraf, S. A., … Youle, R. J. (2014). PINK1 phosphorylates ubiquitin to activate Parkin E3 ubiquitin ligase activity. *The Journal of Cell Biology*, *205*(2), 143–153. https://doi.org/10.1083/jcb.201402104
2. Koyano, F., Okatsu, K., Kosako, H., Tamura, Y., Go, E., Kimura, M., … Matsuda, N. (2014). Ubiquitin is phosphorylated by PINK1 to activate parkin. *Nature*, *510*(7503), 162–166. https://doi.org/10.1038/nature13392
3. Shiba-Fukushima, K., Arano, T., Matsumoto, G., Inoshita, T., Yoshida, S., Ishihama, Y., … Imai, Y. (2014). Phosphorylation of mitochondrial polyubiquitin by PINK1 promotes Parkin mitochondrial tethering. *PLoS Genetics*, *10*(12), e1004861. https://doi.org/10.1371/journal.pgen.1004861
4. Okatsu, K., Koyano, F., Kimura, M., Kosako, H., Saeki, Y., Tanaka, K., & Matsuda, N. (2015). Phosphorylated ubiquitin chain is the genuine Parkin receptor. *The Journal of Cell Biology*, *209*(1), 111–128. https://doi.org/10.1083/jcb.201410050
5. Fiesel, F. C., Ando, M., Hudec, R., Hill, A. R., Castanedes-Casey, M., Caulfield, T. R., … Springer, W. (2015). (Patho-)physiological relevance of PINK1-dependent ubiquitin phosphorylation. *EMBO Reports*, *16*(9), 1114–1130. https://doi.org/10.15252/embr.201540514
6. Klosowiak, J. L., Park, S., Smith, K. P., French, M. E., Focia, P. J., Freymann, D. M., & Rice, S. E. (2016). Structural insights into Parkin substrate lysine targeting from minimal Miro substrates. *Scientific Reports*, *6*, 33019. https://doi.org/10.1038/srep33019
7. Ando, M., Fiesel, F. C., Hudec, R., Caulfield, T. R., Ogaki, K., Gorka-Skoczylas, P., … Springer, W. (2017). The PINK1 p.I368N mutation affects protein stability and ubiquitin kinase activity. *Molecular Neurodegeneration*, *12*(1), 32. https://doi.org/10.1186/s13024-017-0174-z

**PNCK-MYC**

1. Oster, S. K., Ho, C. S. W., Soucie, E. L., & Penn, L. Z. (2002). The myc oncogene: MarvelouslY Complex. *Advances in Cancer Research*, *84*, 81–154.

**PNCK-PTPN11**

1. Daakour, S., Hajingabo, L. J., Kerselidou, D., Devresse, A., Kettmann, R., Simonis, N., et. al. Twizere, J.-C. (2016). Systematic interactome mapping of acute lymphoblastic leukemia cancer gene products reveals EXT-1 tumor suppressor as a Notch1 and FBWX7 common interactor. *BMC Cancer*, *16*, 335. https://doi.org/10.1186/s12885-016-2374-2

**PNCK-UBC**

1. Na, C. H., Jones, D. R., Yang, Y., Wang, X., Xu, Y., & Peng, J. (2012). Synaptic protein ubiquitination in rat brain revealed by antibody-based ubiquitome analysis. *Journal of Proteome Research*, *11*(9), 4722–4732. https://doi.org/10.1021/pr300536k

**PRKCE-MAPT**

1. Zhu, X., Rottkamp, C. A., Boux, H., Takeda, A., Perry, G., & Smith, M. A. (2000). Activation of p38 kinase links tau phosphorylation, oxidative stress, and cell cycle-related events in Alzheimer disease. *Journal of Neuropathology and Experimental Neurology*, *59*(10), 880–888.
2. Sun, W., Qureshi, H. Y., Cafferty, P. W., Sobue, K., Agarwal-Mawal, A., Neufield, K. D., & Paudel, H. K. (2002). Glycogen synthase kinase-3beta is complexed with tau protein in brain microtubules. *The Journal of Biological Chemistry*, *277*(14), 11933–11940. https://doi.org/10.1074/jbc.M107182200
3. Sawamura, N., Gong, J.-S., Chang, T.-Y., Yanagisawa, K., & Michikawa, M. (2003). Promotion of tau phosphorylation by MAP kinase Erk1/2 is accompanied by reduced cholesterol level in detergent-insoluble membrane fraction in Niemann-Pick C1-deficient cells. *Journal of Neurochemistry*, *84*(5), 1086–1096.
4. Sahara, N., Vega, I. E., Ishizawa, T., Lewis, J., McGowan, E., Hutton, M., et. al. Yen, S.-H. (2004). Phosphorylated p38MAPK specific antibodies cross-react with sarkosyl-insoluble hyperphosphorylated tau proteins. *Journal of Neurochemistry*, *90*(4), 829–838. https://doi.org/10.1111/j.1471-4159.2004.02558.x
5. Liu, S. J., Zhang, J. Y., Li, H. L., Fang, Z. Y., Wang, Q., Deng, H. M., et. al. Wang, J. Z. (2004). Tau becomes a more favorable substrate for GSK-3 when it is prephosphorylated by  PKA in rat brain. *The Journal of Biological Chemistry*, *279*(48), 50078–50088. https://doi.org/10.1074/jbc.M406109200
6. Timm, T., Matenia, D., Li, X.-Y., Griesshaber, B., & Mandelkow, E.-M. (2006). Signaling from MARK to tau: regulation, cytoskeletal crosstalk, and pathological  phosphorylation. *Neuro-Degenerative Diseases*, *3*(4–5), 207–217. https://doi.org/10.1159/000095258
7. Singh, T. J., Grundke-Iqbal, I., & Iqbal, K. (1995). Phosphorylation of tau protein by casein kinase-1 converts it to an abnormal Alzheimer-like state. *Journal of Neurochemistry*, *64*(3), 1420–1423.
8. Ekinci, F. J., & Shea, T. B. (1997). Selective activation by bryostatin-1 demonstrates unique roles for PKC epsilon in neurite extension and tau phosphorylation. *International Journal of Developmental Neuroscience : The Official Journal of the International Society for Developmental Neuroscience*, *15*(7), 867–874.

**STK38-MDM2**

1. Yamauchi, T., Nishiyama, M., Moroishi, T., Yumimoto, K., & Nakayama, K. I. (2014). MDM2 mediates nonproteolytic polyubiquitylation of the DEAD-Box RNA helicase DDX24. *Molecular and Cellular Biology*, *34*(17), 3321–3340. https://doi.org/10.1128/MCB.00320-14

**STK38-MOB1A**

1. Devroe, E., Erdjument-Bromage, H., Tempst, P., & Silver, P. A. (2004). Human Mob proteins regulate the NDR1 and NDR2 serine-threonine kinases. *The Journal of Biological Chemistry*, *279*(23), 24444–24451. https://doi.org/10.1074/jbc.M401999200
2. Bichsel, S. J., Tamaskovic, R., Stegert, M. R., & Hemmings, B. A. (2004). Mechanism of activation of NDR (nuclear Dbf2-related) protein kinase by the hMOB1 protein. *The Journal of Biological Chemistry*, *279*(34), 35228–35235. https://doi.org/10.1074/jbc.M404542200
3. Hutchins, J. R. A., Toyoda, Y., Hegemann, B., Poser, I., Heriche, J.-K., Sykora, M. M., et. al. Peters, J.-M. (2010). Systematic analysis of human protein complexes identifies chromosome segregation proteins. *Science (New York, N.Y.)*, *328*(5978), 593–599. https://doi.org/10.1126/science.1181348
4. Kohler, R. S., Schmitz, D., Cornils, H., Hemmings, B. A., & Hergovich, A. (2010). Differential NDR/LATS interactions with the human MOB family reveal a negative role for human MOB2 in the regulation of human NDR kinases. *Molecular and Cellular Biology*, *30*(18), 4507–4520. https://doi.org/10.1128/MCB.00150-10
5. So, J., Pasculescu, A., Dai, A. Y., Williton, K., James, A., Nguyen, V. et. al. Colwill, K. (2015). Integrative analysis of kinase networks in TRAIL-induced apoptosis provides a source of potential targets for combination therapy. *Science Signaling*, *8*(371), rs3. https://doi.org/10.1126/scisignal.2005700
6. Joffre, C., Dupont, N., Hoa, L., Gomez, V., Pardo, R., Goncalves-Pimentel, C., et. al. Camonis, J. (2015). The Pro-apoptotic STK38 Kinase Is a New Beclin1 Partner Positively Regulating Autophagy. *Current Biology : CB*, *25*(19), 2479–2492. https://doi.org/10.1016/j.cub.2015.08.031

**TRIO-ESR1**

1. Notas, G., Kampa, M., Pelekanou, V., Troullinaki, M., Jacquot, Y., Leclercq, G., & Castanas, E. (2013). Whole transcriptome analysis of the ERalpha synthetic fragment P295-T311 (ERalpha17p) identifies specific ERalpha-isoform (ERalpha, ERalpha36)-dependent and -independent actions in breast cancer cells. *Molecular Oncology*, *7*(3), 595–610. https://doi.org/10.1016/j.molonc.2013.02.012

**TRIO-MYC**

1. Hein, M. Y., Hubner, N. C., Poser, I., Cox, J., Nagaraj, N., Toyoda, Y., et. al. Mann, M. (2015). A human interactome in three quantitative dimensions organized by stoichiometries and abundances. *Cell*, *163*(3), 712–723. https://doi.org/10.1016/j.cell.2015.09.053

**Table S7 Primers used in this study**

| **Primer name** |  **Sequence** |
| --- | --- |
| ***HPRT* gene reconstitution** | |
| Lox137-R | 5′-AGCCTTCTGTACACATTTCTTCTC- 3′ |
| Rev #65′ | 5'- GCTCTACTAAGCAGATGGCCACAGAACTAG-3′ |
| **Primers used for construction of the plasmids** | |
| EcoRI-GFP-FWD | 5'-AGAA TTCGCCACCA TGGTGAGCA-3′ |
| EcoRI-GFP-hCDT1-REV | 5'-TGAATTCTTAGATGGTGTCCTGGTCCT-3′ |
| EcoRI-GFP-hGEMININ-REV | 5'-TGAATTCTTACAGCGCCTTTCTCCG-3′ |
| **Primers detecting hamster *SINE*s** | |
| B2-F | 5′-GCTCAGAGGTTAAGAGCACTGAC-3′ |
| B2-R | 5′-TGCTTCCA TGT A T A TCTGCACAC-3′ |

**Table S8 Antibodies used for Western blot and immunofluorescence**

|  **Primary antibodies** | | | | |
| --- | --- | --- | --- | --- |
| **Protein name** | **Catalog #** | **Size** | **Dilution** | **Company/Lab** |
| IRAK1 | sc-5288 | 80 kDa | WB 1:200 | Santa Cruz |
| STK38 | H00011329-M01 | 54 kDa | WB 1:500 | Abnova |
| TRIO | A304-269A-T | 346 kDa | WB 1:500 | Bethyl |
| PRKCE | sc-214 | 90 kDa | WB 1:200 | Santa Cruz |
| PNCK | AP16935b-ev | 38,5 kDa | WB 1:500 | Abgent |
| PINK1 | 6946 | 60, 50 kDa | WB 1:500 | Cell Signaling |
| BUB1 | 13330-1-AP | 45 kDa | WB 1:500 | ProtTech |
| BUB1B | 11504-2-AP | 120-130 kDa | WB 1:500 | ProtTech |
| TAOK1 | 26250-1-AP | 116 kDa | WB 1:500 | ProtTech |
| ITPKB | 12816-1-AP | 102-110 kDa | WB 1:500 | Protein Tech |
| MYLK | 21642-1-AP | 135 kDa | WB 1:500 | Protein Tech |
| TNK2 (ACK1) | 14304-1-AP | 70 kDa | WB 1:500 | Protein Tech |
| BLK | 10510-1-AP | 58 kDa | WB 1:500 | Protein Tech |
| FRK | 16197-1-AP | 54-57 kDa | WB 1:500 | Protein Tech |
| STK11 (LKB) | - | 45-49 kDa | WB 1:1000 | Dr. Lou's Lab, NCI |
| CSK | 17720-1-AP | 50 kDa | WB 1:1000 | Protein Tech |
| PDXK | 15309-1-AP | 40 kDa, 35 kDa | WB 1:500 | Protein Tech |
| PHKG1 | 16743-1-AP | 40 kDa | WB 1:500 | Protein Tech |
| CAMK2G | 12666-2-AP | 59 kDa | WB 1:1000 | Protein Tech |
| PHKB | 13400-1-AP | 124 kDa | WB 1:500 | Protein Tech |
| BTK | - | 70-76 kDa | WB 1:500 | Dr. Wiest's lab, NCI |
| HIPK2 | 55408-1-AP | 131 kDa, 101 kDa | WB 1:200 | Protein Tech |
| ATM | - | 350 kDa | WB 1:500 | Dr. Dasso's lab, NICHD |
| NEK9 | 11192-1-AP | 120 kDa | WB 1:300 | Protein Tech |
| CENPE | - | 300-316 kDa | WB 1:200 | Dr. Dasso's lab, NICHD |
| CENPN | 16751-1-AP | 40 kDa, 25-30 kDa | WB 1:500 | Protein Tech |
| AURKB | - | 34 kDa | WB 1:500 | Dr. Dasso's lab, NICHD |
| CENPA | - | 16 kDa | WB 1:500 | Dr. Masumoto's Lab, Japan |
| NAT10 | - | 116 kDa | WB 1:500 | Hunter's lab, NCI |
| PIGB | 12612-1-AP | 50-55 kDa | WB 1:500 | Protein Tech |
| PIGS | 18334-1-AP | 65-70 kDa | WB 1:200 | Protein Tech |
| PRC1 | 15617-1-AP | 66 kDa | WB 1:500 | Protein Tech |
| IPO11 | 14403-1-AP | 112 kDa | WB 1:500 | Protein Tech |
| CIAO2B (FAM96B) | 20108-1-AP | 18-20 kDa | WB 1:500 | Protein Tech |
| MSI1 | 27185-1-AP | 35 kDa | WB 1:500 | Protein Tech |
| AP2B1 | 15690-1-AP | 100 kDa-115 kDa | WB 1:1000 | Protein Tech |
| WDR76 | 25528-1-AP | 70 kDa | WB 1:200 | Protein Tech |
| PLCD3 | 16792-1-AP | 85 kDa | WB 1:500 | Protein Tech |
| MUC4 | 55343-1-AP | 235-250 kDa | WB 1:500 | Protein Tech |
| NF1 | 27249-1-AP | 319 kDa | WB 1:500 | Protein Tech |
| RAB1A | 11671-1-AP | 23 kDa | WB 1:1000 | Protein Tech |
| MEMO1 | 14604-1-AP | 31-34 kDa | WB 1:500 | Protein Tech |
| RPL13 | 11271-1-AP | 24 kDa | WB 1:500 | Protein Tech |
| XAB2 | 10637-1-AP | 100 kDa | WB 1:200 | Protein Tech |
| GAPDH | 14C10 | 37 kDA | WB 1:1000 | Cell Signaling |
| Tubulin alpha | T6199 | 50 kDa | IF 1:300 | Sigma |
| MAD1 | - | 83 kDA | IF 1:300 | Dr. Dasso's lab, NIH |
| **Secondary antibodies** | | | | |
| goat-anti-mouse HRP | sc-2302 |  | WB 1:5000 | Santa Cruz |
| goat-anti-rabbit HRP | sc-2004 |  | WB 1:5000 | Santa Cruz |
| anti-mouse-Alexa 647 | 4410 |  | IF 1:500 | Cell Signaling |
| anti-rabbit-Alexa 555 | 4413 |  | IF 1:500 | Cell Signaling |

**Тable S9 Percentage of HAC/dGFP and GFP-expressing cells in HT1080 during cultivation in the medium with blasticidin**

| **Detection day** | **Percentage of GFP-positive cells**  (detected by FACS) | **Percentage of HAC-positive cells**  (detected by FISH) |
| --- | --- | --- |
| Day 1 | 91.3 | 98.2 |
| Day 10 | 92.7 | 97.5 |
| Day 20 | 93.5 | 99.0 |
| Day 30 | 92.1 | 97.3 |
| Day 31 | 92.5 | 97.6 |

**Supplemental Methods**

**Cell lines and culture**

The human fibrosarcoma (HT1080; ATCC® CCL-121™) and hTERT RPE-1 (ATCC^®^ CRL-4000^™^) cell lines were obtained from the American Type Culture Collection and were authenticated both morphologically and by short tandem repeat analysis. All cell lines were tested regularly to confirm lack of mycoplasma infection with mycoplasma detection kit PlasmoTest from InvivoGen. The HT1080 cell line containing the alphoid^tetO^-HAC was cultured in Dulbecco’s Modified Eagle’s medium (DMEM) (Thermo Fisher Scientific) supplemented with 10% (v/v) fetal bovine serum (Clontech Laboratories, Inc.) at 37^o^C in 5% CO_2_ atmosphere in the presence of 10 μg/ml Blasticidin S (Thermo Fisher Scientific). Hypoxanthine phosphoribosyl transferase (HPRT)-deficient Chinese hamster ovary (CHO) cells (JCRB0218) carrying the alphoid^tetO^-HAC were maintained in Ham's F-12 nutrient mixture (Thermo Fisher Scientific) plus 10% FBS with 8 µg/ml of Blasticidin (Thermo Fisher Scientific). After loading of the p264-GFP-CDT1-GFP-GEMININ vector (see below) into the alphoid^tetO^-HAC, the CHO cells were cultured in presence of 1× HAT (Thermo Fisher Scientific) supplemented medium in the presence of 10 μg/ml Blasticidin S (Thermo Fisher Scientific). Human retina, eye, pigmented epithelium (RPE) cells were routinely cultured in Dulbecco’s Modified Eagle’s medium (DMEM) (Thermo Fisher Scientific) supplemented with 10% (v/v) fetal bovine serum (Clontech Laboratories, Inc.) at 37^o^C in 5% CO_2_ atmosphere.

**FISH analysis**

HT1080 cells containing the HAC were grown in DMEM medium to 70-80% confluence. Metaphase cells were obtained by adding colcemid (Gibco) to a final concentration of 0.05 μg/ml and incubating overnight. Media was aspirated and the plate was washed with 1× PBS. Cells were removed from the plate by 0.25% Typsin, washed off with DMEM, pelleted and resuspended in 10 ml of 50 mM KCl hypotonic solution and incubated for 30 min at 37°C. Cells were fixed by three washes of fixative solution (75% acetic acid, 25% methanol). Between each wash, cells were pelleted by centrifugation at 150 g for 4 min. Metaphase cells were evenly spread on a microscope slide and the fixative solution evaporated over boiling water. Dry slides were rehydrated with 1× PBS for 15 min, and fixed in 4% formaldehyde in 1× PBS for 2 min, followed by three 5 min 1× PBS washes and ethanol series (of 70%, 90% and 100%, correspondingly) dehydration. The PNA (peptide nucleic acid) DNA labeled probes were for telomere (CCCTAA)3-Cy3) (Panagene, South Korea) and for tetO-alphoid array (FITC-OO-ACCACTCCCTATCAG) (Panagene, South Korea). Ten nanomol of each PNA probe was mixed with hybridization buffer (10 mM Tris-HCl, pH 7.4; 70% Formamide; 5% Dextran sulfate) and applied to the slide, followed by denaturation at 80°C for 3 min. Slides were hybridized for 2 hr at RT in the dark. Slides were washed twice in 70% formamide, 10 mM Tris pH 7.2, 0.1% BSA followed by three washes with 1× TBS, 0.08% Tween-20. Slides were dehydrated gradually with a series of 70%, 90% and 100% ethanol washes and mounted (Vectorshield with DAPI). Images were captured using a Zeiss Microscope (Axiophot) equipped with a cooled-charge-coupled device (CCD) camera (Cool SNAP HQ, Photometric) and analyzed by IP lab software (Signal Analytics). The PNA-DNA hybrid probes demonstrated a high hybridization efficiency, staining intensity and adopt a stable duplex form with complementary nucleic acid.

**Western blot analysis**

Decrease in  the protein level  after siRNA transfection was monitored by Western blot analysis. HT1080 cells containing the HAC/dGFP were cultivated for 3 days and then seeded on 24-well plate in concentration 12.5×10^3^ cells per well. siRNA targeting sequences used for knockdown of a gene of interest are presented in Supplemental Table S1. For each gene, the cells were transfected with siRNA and then grown for 72 hr. Cell pellets were collected from a set of 4 wells of a 24-well plate and transferred to 1.5 ml Eppendorf tubes. Cell suspensions were centrifuged at 3000g for 3 min and washed with PBS. Then the cell pellets were frozen at -80^o^C. To extract proteins, cell pellets were suspended in 100 μl tissue lysis buffer [20 mM Tris-HCl pH 7.5; 125 mM NaCl; 1 mM EDTA; 1% Triton X-1001%; Proteinase/Phosphatase inhibitors) (Sigma-Adrich)]. To complete homogenization, the lysates were sonicated for a 10 min at 4^o^C. After that the samples were centrifuged at 16,000g for 5 min at 4^o^C. Then the protein concentrations of the samples was measured using Bradford assay (Bio-Rad). Test samples with equal concentrations (40 µg per well) proceeded via the standard protocol of SDS-PAGE Laemmli gel electrophoresis (SDS-PAGE) (Laemmli 1970). Western blotting was carried out in SDS Running Buffer (Thermo Fisher Scientific) with 20% of methanol using nitrocellulose membranes at 120V for 1 hr. After transfer procedure, the membranes were incubated in 5% skimmed milk, 0.1% Tween in PBS for 1 hr, then washed 2 times at RT for 2 min with 0.1% Tween in PBS and incubated with the primary antibodies against the target protein overnight at 4^o^C. The membranes were washed 3 times with 0.1% Tween in PBS for 15 min and incubated with the secondary antibodies for 1 hr. After that the membranes were washed 3 times with 0.1% Tween in PBS for 15 min. For membrane visualization, chemiluminescent substrates for horseradish peroxidase (HRP) was used (Thermo Fisher Scientific). The images of the membranes were taken using ChemiDoc™ Imaging Systems (Bio-Rad). The antibodies list is presented in Supplemental Table S8.

**Immunocytochemistry**

siRNA treated cells were fixed in 4% PFA in PBS for 15 min at RT. Cells were rinsed 2 times quickly with PBS to remove fixation solution at RT. 200 μl of 5% BSA in PBS-TT (PBS containing 0.5% Tween 20, 0.1% Triton X-100) were added to washed cells. Cells were incubated for 30 min at RT for blocking. Cells were washed 3 times with PBS-T (PBS, containing 0.1% Tween 20) for 5 min. 200 μl of the first antibody (dilution according to the manufacture’s protocol) in 1% BSA in PBS-TT were added to cells and then the cells were incubated at RT for 2 hs. The samples were washed 3 times with PBS-T for 5 min. 200 μl of the secondary antibodies (anti-mouse Alexa 647, dilution 1:500, CellSignaling 4410S; anti-rabbit Alexa 555, dilution 1:500, CellSignaling 4413S) in 1% BSA in PBS-TT were applied at RT for 1 hr. The samples were washed 3 times in PBS-T for 5 min. The samples were counterstained with DAPI and mount with mounting media (ProLong™ Diamond Antifade Mountant with DAPI, Life Technology, P36962). The samples obtained were analyzed using Confocal Microscope System Zeiss LSM780, LRBGE Fluorescence Imaging Facility (NIH). The antibodies list is presented in Supplemental Table S8.

**Bioinformatical data for high-content image analysis and a network**

The well level screen data generated by Columbus were analyzed using R 3.3.2 [R Core Team (2018)] where R is a language and environment for statistical computing (R Foundation for Statistical Computing, Vienna, Austria; URL https://www.R-project.org/ and the cellHTS2 2.36 package) (Boutros et al. 2006). The percentage of GFP- cells and the number of cells per well was calculated from Columbus and from the tab-separated text files. The data was normalized per plate basis by the first subtracting the siRNA oligo library median from each measurement and by then dividing this value by the siRNA oligo library median absolute deviation (MAD). Plate normalized values were ranked screen-wide by calculating z-scores, based on their distribution. z-scores for each biological replicates were aggregated by calculating the mean. Qiagen Ingenuity Pathway Analysis (IPA https://www.qiagenbioinformatics.com/products/ingenuity-pathway-analysis/) was used to discover known relationships between the genes in our list (Supplemental Table S6). Using direct and indirect connections from 18 new genes, we found a well connected network of 24 genes from our list (see Supplemental Fig. S13 and Supplemental Table S6).

**Transcription profiles and cytogenetic alterations in the NCI-60 cell lines**

We used expression data for theNCI-60 from five different arrays (Agilent Whole Human Genome Oligo Microarray 44K, and Affymetrix Human Genome U95, U133, and U133 Plus 2.0, and GeneChip Human Exon 1.0 ST HuEx). Quantitation of gene transcript expression levels was done as described previously (Reinhold et al. 2015). Cytogenetic alterations determined as described previously were compared to the gene transcript levels using the Pattern Comparison web-application (https://discover.nci.nih.gov/cellminer/) (Roschke et al. 2003).

**Calculation of the rate of HAC loss induced by siRNA-mediated knockdown of a target gene**

The HAC/dGFP is stably propagated in human HT1080 cells, i.e. almost every cell contains one copy of the HAC/dGFP expressing the GFP-CDT1-GFP-GEMININ cassette (Supplemental Table S9). The HAC is less stable than host chromosomes (Lee et al. 2016). Therefore, if the HAC contains the *GFP* transgene, its loss can be measured by flow cytometry or fluorescence scanning microscopy. In this study HAC loss after siRNA-mediated knockdown of a target gene is calculated based on the proportion of GFP- positive (x) and GFP-negative cells (y) in total cells population (Lee et al. 2013). Proportion (P_o_) of GFP- positive cells is calculated using the following formula:


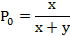


To measure HAC loss per one cell division, we introduced a new parameter (R), that means a probability of HAC-loss during cell division. The cells carrying the HAC are usually grown under blasticidin selection. The rate of HAC-loss is measured without selection when the cells start losing the HAC spontaneously. This parameter can be changed depending on the medium conditions and knockdown of a gene of interest. R_norm_ is the probability of HAC loss in the medium without selection. R1 is the probability of HAC loss after siRNA treatment. When cells are grown without selection and without siRNA treatment, the number of GFP-positive cells per one division is


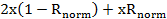


and the number of GFP-negative cells is


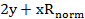


The proportion of GFP-positive cells is


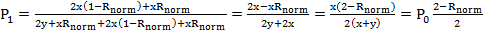

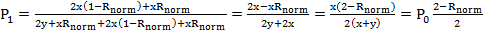


and after n divisions is


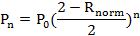


The proportion of siRNA-treated cells after one cell division is:

One average cell cycle of the HAC/dGFP containing HT1080 cells takes approximately 18 hours. This parameter was counted by recording the growth of cell population at multiple time points using cell tracking technique. Based on the average time of cell division, the calculation can be changed specifically for the day of GFP loss detection. In this study, the calculation was optimized specially for high-throughput siRNA library screening where we detected GFP loss after more than 600 deferent gene knockdown. To optimize this calculation, we used an average doubling time of HT1080 cells containing the HAC/dGFP, i.e 18 hours. Therefore, after 72 hours the growing cells will go through 4 cell cycles (n = 72/18 = 4).

Thus, the proportion of GFP-positive cells after 4 divisions is

Final calculation can be modified using the following formula:

;

The formula to count the probability of HAC-loss per cell division becomes

**Genomic DNA preparation and PCR analysis**

Genomic DNA was prepared using QIAmp DNA Mini Kit (QIAGEN Inc., Valencia, CA, USA). Reconstitution of the *HPRT* gene after Cre/lox-mediated recombination was determined by a pair of specific primers, Lox137-R and Rev #6 (Supplemental Table S7). Cross contamination by hamster chromosomes was determined by specific primers detecting hamster SINEs (Supplemental Table S7). PCR products for sequencing were separated by agarose gel electrophoresis and then gel extracted using Wizard® SV Gel and PCR Clean-Up System (Promega).

**Cytokinesis-block micronucleus assay**

Quadruplicate cultures of cells in 24-well plates were exposed to different siRNAs or scramble siRNA as a negative control. After 72 hours of cultivation Cytochalasin B was added to final concentration 4.5 μg/ml for 24 hours. The cells were trypsinized and 5×10^3^ cells were span down onto cytoslides (Shandon, # 5991056) at 1,000 rpm for 1 min in Cytospin 3 (Shandon). The slides were air-dried for 5 min, fixed with Diff-Quick fixative for 5 min, stained in Diff-Quick solution C (Eosin Y) (Electron Microscopy Sciences, # 26096) for 10 seconds, rinsed in distilled water and dried for 5 min. Coverslips were mounted with ProLong® Diamond Antifade Mountant with DAPI (Thermo Fisher Scientific, # P36962). About 100 binucleated cells on each slide were scored for the presence of micronuclei (MNi) or nucleoplasmic bridges (NPBs).

**Statistical analysis**

The statistical significance of comparisons between two groups was determined with Student’s *t*-test.

P values of less than 0.05 were considered statistically significant. For multiple testing, Fisher's exact test with Bonferroni correction was used.

**CRISPR/Cas9 gene disruption**

The CRISPR/Cas9 gene disruption experiments have been performed as previously published (Doench et al. 2016; Tzelepis et al. 2016). pKLV2-EF1a-Cas9Bsd-W was a gift from Dr. Kosuke Yusa (Addgene plasmid #68343; http://n2t.net/addgene:68343; RRID:Addgene_68343). TRIO gRNA (BRDN0001487141) was a gift from Dr. John Doench and Dr. David Root (Addgene plasmid #78034; http://n2t.net/addgene:78034; RRID:Addgene_78034). PINK1 gRNA (BRDN0001144744) was a gift from Dr. John Doench and Dr. David Root (Addgene plasmid #78038; http://n2t.net/addgene:78038; RRID:Addgene_78038). STK38L gRNA (BRDN0001145817) was a gift from Dr. John Doench and Dr. David Root (Addgene plasmid # 76420 ; http://n2t.net/addgene:76420 ; RRID:Addgene_76629). PNCK gRNA (BRDN0001145068) was a gift from Dr. John Doench and Dr. David Root (Addgene plasmid # 76420 ; http://n2t.net/addgene:76420 ; RRID:Addgene_77328). IRAK1 gRNA (BRDN0001145661) was a gift from Dr. John Doench and Dr. David Root (Addgene plasmid # 76420 ; http://n2t.net/addgene:76420; RRID:Addgene_76420). TAOK1 gRNA (BRDN0001148505) was a gift from Dr. John Doench and DrDavid Root (Addgene plasmid # 76420 ; http://n2t.net/addgene:76420 ; RRID:Addgene_76806).

**Live-cell imaging**

To perform live-cell imaging RPE cells were labeled with histone H2B fused with mCherry protein (pLenti6-H2B-mCherry was a gift from Dr. Torsten Wittmann (Addgene plasmid #89766; http://n2t.net/addgene:89766 ; RRID:Addgene_89766)). The cells were sited and transfected identically to siRNA library screening (see Methods). The cells were imaged using a Yokogawa CV7000S spinning disk confocal microscope supplied with an incubator for live-cell imaging with 5% CO_2_ atmosphere with Olympus 40X (NA 0.95) PlanApoChromat lens, an emission 405/488/561/640 dichroic mirror and a 16-bit sCMOS camera (2550×2160 pixels) with pixel binning set to 2×2. The cells were imaged at 80 hr after transfection during 16 hr with the 3 min recording interval.

**References**

1. Doench JG, Fusi N, Sullender M, Hegde M, Vaimberg EW, Donovan KF, Smith I, Tothova Z, Wilen C, Orchard R et. al. 2016. Optimized sgRNA design to maximize activity and minimize off-target effects of CRISPR-Cas9. *Nat Biotechnol* **34**: 184-191.
2. Tzelepis K, Koike-Yusa H, De Braekeleer E, Li Y, Metzakopian E, Dovey OM, Mupo A, Grinkevich V, Li M, Mazan M et. al. 2016. A CRISPR Dropout Screen Identifies Genetic Vulnerabilities and Therapeutic Targets in Acute Myeloid Leukemia. *Cell Rep* **17**: 1193-1205.
3. Boutros M, Bras LP, Huber W. 2006. Analysis of cell-based RNAi screens. *Genome Biol* **7**: R66.
4. Laemmli UK. 1970. Cleavage of structural proteins during the assembly of the head of bacteriophage T4. *Nature* 227: 680-685.
5. Roschke AV, Tonon G, Gehlhaus KS, McTyre N, Bussey KJ, Lababidi S, Scudiero DA, Weinstein JN, Kirsch IR. 2003. Karyotypic complexity of the NCI-60 drug-screening panel. *Cancer Res* **63**: 8634-8647.
6. Lee HS, Lee NC, Grimes BR, Samoshkin A, Kononenko AV, Masumoto H, Earnshaw WC, Kouprina N, Larionov V. 2013. A new assay for measuring chromosome instability (CIN) and dentification of drugs that elevate CIN in cancer cells. *BMC Cancer* **13**:252.
7. Lee HS, Lee NC, Kouprina N, Kim JH, Kagansky A, Bates S, Trepel JB, Pommier Y, Sackett D, Larionov V. 2016. Effects of Anticancer Drugs on Chromosome Instability and New Clinical Implications for Tumor-Suppressing Therapies. *Cancer Res* **76**: 902-911.
8. Reinhold WC, Sunshine M, Varma S, Doroshow JH, Pommier Y. 2015. Using CellMiner 1.6 for Systems Pharmacology and Genomic Analysis of the NCI-60. *Clinical Cancer Research* **21**: 3841-3852.
